# Supplementary material for: Nɛ-acetyl lysine derivatives with zinc binding groups as novel HDAC inhibitors
Source: R Soc Open Sci. 2019 Jun 5;6(6):190338. doi: 10.1098/rsos.190338 (PMC6599804; doi:10.1098/rsos.190338)
Supplement: Synthetic and biological experiments [file rsos190338supp1.pdf]

Supplementary Information

N^{ε}-acetyl-lysine derivatives with Zinc binding groups as novel HDAC inhibitors

Fang Wang,[a,c] Chun Wang,[a,c] Jie Wang,[a,c] Yefang Zou, [a,c] Xiaoxue Chen,[a,c] Ting Liu,[b,c] Yan Li,[a,d] Yonglong Zhao,[a,c] Yongjun Li,[a,c] Bin He*[a,c]

[a] *State Key Laboratory of Functions and Applications of Medicinal Plants, Engineering Research Center for the Development and Application of Ethnic Medicine and TCM (Ministry of Education), Guizhou Medical University, Guiyang 550004, China.*

[b] *Guizhou Provincial Key Laboratory of Pharmaceutics, Guizhou Medical University, Guiyang 550004, China.*

[c] *School of Pharmacy, Guizhou Medical University, Guiyang 550004, China.*

[d] *School of Basic Medicine, Guizhou Medical University, Guiyang 550004, China.*

** To whom correspondence should be addressed to: binhe@gmc.edu.cn.*

Contents:

General information and materials S1
Synthesis and characterization of compounds **8-11** S2
Synthesis and characterization of compounds **14a-q** S4
Synthesis and characterization of compounds **15a-c** and **16a-c** S9
Synthesis and characterization of compounds **17a-c** and **18b-c** S11
HDACs inhibition assay *in vitro* S13
Cell Proliferation Assay S13
Western Blot Methods S14
Supplementary references S15
Supplementary references S15
^{1}H and ^{13}C NMR spectra of compounds **8-11** S16
^{1}H and ^{13}C NMR spectra of compounds **14a-q** S20
^{1}H and ^{13}C NMR spectra of compounds **15a-c** and **16a-c** S35
^{1}H and ^{13}C NMR spectra of compounds **17a-c** and **18b-c** S41

General information and materials

General: The following materials were obtained from commercial sources for the compound preparation, and were used as received without further treatment, and all reagents and solvents were commercial high purity quality. The purity of all tested compounds was over 95% by HPLC.

Materials: Energy Chemical ShangHai: N-alpha-Cbz-L-lysine, N-methylmorpholine (NMM), Isobutyl chloroformate (IBCF), Trifluoroacetic acid (TFA), Triethylamine (TEA), Tetrahydrofuran (DMF), 4-dimethylamino pyridine (DMAP), tert-butyl alcohol, 2-(1H-benzotriazole-1-yl)-1,1,3,3-tetramethy laminium hexafluorophosphate (HBTU), N,N-Diisopropylethylamine (DIEA), Hydroxylamine hydrochloride, o-Phenylenediamine, Succinic anhydride, Maleic anhydride, Nicotinic acid, 3-Indoleformic acid, 6-Methylpyridine-3-carboxylic acid, 3-(3-pyridyl) acrylic acid), Pyrrole-2-carboxylic acid, 2-Furoic acid, 2-Thiophen-ecarboxylic acid, Mercaptoacetic acid, 3-Quinolinecarboxylic acid, 6-Quinolinecarboxylic acid, 3-Indoleformic acid, 1H-Indene-3-carboxylic acid, Indole-3-carbox -aldehyde, 1-Benzothiophene-3-carboxylic acid, Benzofuran-3-carboxylic acid, (2,4-Dioxo-1,3-thiazolidin-5-yl)acetic acid, N-[(tert-Butoxy)carbonyl]-L-tryptophan, Suberic acid, Monomethyl ester, Monomethyl Malonate, Monomethyl adipate. Aladdin ShangHai: LiOH. Boc-Lys(Ac)-AMC .

^{1}H and ^{13}C-NMR were performed on JEOL ESC-400 spectrometers.

Synthesis and characterization of new compounds

(8): 2-(benzyloxycarbonyl)-6-(tert-butoxycarbonyl)hexanoic acid (5.3g, 13.9mmol), DIEA (4.8mL, 27.8mmol) and HBTU (7.9g, 20.85mmol) was added in anhydrous THF (100mL), and then the aniline (1.9mL, 20.85mmol) was added, after that the reaction was stirred 3h. The THF was removed under reduced pressure, the reaction mixture was diluted with CH_2Cl_2, and saturated NaCl solution, the organic layer was washed with saturated NaCl, and dried over Na_2SO_4 and concentrated. The residue was purified by silica gel column chromatography (5.95g, 93%). The compound **7** purified above (5.95g, 13.07mmol) was added in anhydrous CH_2Cl_2 (60mL) and cooled (0°C), and then the TFA (30mL) was added slowly, stirred for 2h. Then, the TFA was removed under reduced pressure until there was no TFA, the residue was purified by silica gel column chromatography (4.4g, 95%). ^{1}H NMR (400 MHz, CD_3OD) δ 7.51 (d, *J* = 7.2 Hz, 2H), 7.27 (dt, *J* = 33.0 Hz, 18.4, 7H), 7.07 (t, *J* = 7.2 Hz, 1H), 5.13 – 5.01 (m, 2H), 4.21 (d, *J* = 17.2 Hz, 1H), 2.87 (t, *J* = 7.3 Hz, 2H), 1.92 – 1.58 (m, 4H), 1.56 – 1.33 (m, 2H). ^{13}C NMR (100 MHz, CD_3OD) δ 171.76, 157.43, 137.96, 136.86, 128.51, 127.90, 124.39, 120.35, 66.71, 55.31, 39.63, 32.02, 27.40, 22.25. HRMS (ESI) *m/z* calculated for $C_{20}H_{25}N_3O_3^+$ $(M+H)^+$ 356.1974, found 356.1982.

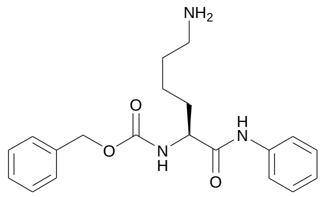

8

(9): Succinic anhydride (56mg, 0.84mmol) was dissolved in anhydrous THF (2mL) and TEA (233ul, 1.68mmol), and the compound **8** (200mg, 0.56mmol) was added slowly, the mixture was stirred for 3 hours at RT. Then, the THF was removed under reduced pressure, the residue was purified by silica gel column chromatography (230mg, 90%). ^{1}H NMR (400 MHz, DMSO-*d6*) δ 12.03 (s, 1H, -COOH), 9.95 (d, *J* = 22.5 Hz, 1H, -CONH-), 7.81 – 7.71 (m, 1H, -CONH-), 7.56 – 7.42 (m, 3H), 7.35 – 7.14 (m, 7H), 6.99 (dd, *J* = 19.7Hz, 12.3, 1H), 5.01 – 4.88 (m, 2H), 4.11 – 3.98 (m, 1H), 2.97 (d, *J* = 5.5 Hz, 2H), 2.38 – 2.29 (m, 2H), 2.25 – 2.17 (m, 2H), 1.57 (dd, *J* = 11.2 Hz, 6.8, 2H), 1.38 – 1.22 (m, 4H). ^{13}C NMR (100 MHz, DMSO-*d6*) δ 174.64, 171.76, 171.25, 156.63, 139.40, 137.30, 129.22, 128.94, 128.27, 123.75, 119.66, 66.12, 55.71, 31.95, 30.45, 29.70, 29.47, 23.64. HRMS (ESI) *m/z* calculated for $C_{24}H_{30}N_3O_6^+$ $(M+H)^+$ 456.2135, found 456.2193.

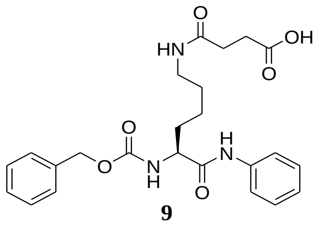

9

(10): The Maleic anhydride (50mg, 0.5mmol) was dissolved in anhydrous THF (2mL) and TEA (250ul, 1.5mmol), and the compound **8** (180mg, 0.5mmol) was added slowly, the mixture was stirred for 3 hours at RT. Then, the THF was removed under reduced pressure, the residue was purified by silica gel column chromatography (205mg, 90%). ^{1}H NMR (400 MHz, DMSO-d_6) δ 9.98 (d, *J* = 11.7 Hz, 1H, -CONH-), 9.10 (d, *J* = 5.8 Hz, 1H, -CONH-), 7.60 – 7.47 (m, 3H), 7.36 – 7.12 (m, 8H), 7.03 – 6.95 (m, 1H), 6.38 – 6.30 (m, 1H), 6.19 (dd, *J* = 15.1 Hz, 9.6, 1H), 4.98 (d, *J* = 12.2 Hz, 2H), 4.07 (s, 1H), 3.10 (dd, *J* = 12.0, 6.0 Hz, 2H), 1.68 – 1.51 (m, 2H), 1.50 – 1.25 (m, 4H). ^{13}C NMR (100 MHz, DMSO-d_6) δ 171.59, 165.83, 156.58, 139.68, 137.52, 133.68, 132.33, 129.28, 128.87, 128.32, 128.25, 123.49, 120.18, 66.59, 55.64, 31.68, 28.37, 23.62. HRMS (ESI) *m/z* calculated for $C_{24}H_{28}N_3O_6^{3+}$ $(M+H)^{3+}$ 456.2135, found 456.2192.

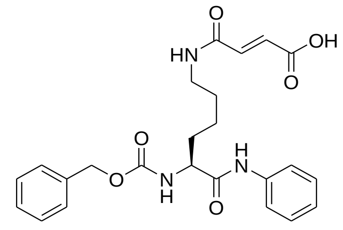

10

(11): The compound **9** (70mg, 0.154mmol) was added in anhydrous THF (3mL), and cooled to 0°C and then the TEA(43μL, 0.308mmol), IBCF (30μL, 0.2316mmol) was added slowly, the mixture was stirred for 1 hours at 0°C to rt, and added Hydroxylamine hydrochloride (53mg, 0.77mmol) in Methanol, after that the reaction was stirred 3h. The THF was removed under reduced pressure, the reaction mixture was diluted with CH_2Cl_2, and saturated NaCl solution, the organic layer was washed with saturated NaCl, and dried over Na_2SO_4 and concentrated. The residue was purified by silica gel column chromatography (30mg, 41%). ^{1}H NMR (400 MHz, CD_3OD) δ 7.56 – 7.41 (m, 2H), 7.37 – 7.11 (m, 7H), 7.05 (dd, *J* = 20.2, 12.8 Hz, 1H), 5.19 – 4.96 (m, 2H), 4.26 – 4.03 (m, 1H), 3.20 – 3.07 (m, 2H), 2.43 (dd, *J* = 13.0, 6.1 Hz, 2H), 2.31 (dd, *J* = 15.9 Hz, 8.7, 2H), 1.91 – 1.61 (m, 2H), 1.62 (s, 4H). ^{13}C NMR (100 MHz, CD_3OD) δ 173.10, 172.03, 170.07, 157.45, 138.25, 136.59, 128.14, 124.27, 120.12, 66.61, 55.64, 38.62, 32.05, 30.66, 29.04, 28.50, 27.66, 24.93, 22.16. HRMS (ESI) *m/z* calculated for $C_{24}H_{31}N_4O_6^{+}$ $(M+H)^{+}$ 471.2244 , found 471.2232.

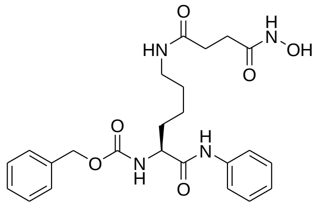

11

(14a): The Nicotinic acid (52mg, 0.42mmol) was added in anhydrous THF (3.5mL), and then the DIEA (98μL, 0.56mmol), HBTU(160mg, 0.42mmol), and The compound **8** (100mg, 0.28mmol), which was dissolved in THF was added, after that the reaction was stirred 4h. The THF was removed under reduced pressure, the reaction mixture was diluted with CH_2Cl_2 , and saturated NaCl solution, the organic layer was washed with saturated NaCl, and dried over Na_2SO_4 and concentrated. The residue was purified by silica gel column chromatography (81mg, 62%). ^{1}H NMR (400 MHz, DMSO-d_6) δ 9.96 (s, 1H, -CONH-), 8.97 – 8.93 (m, 1H, -CONH-), 8.66 – 8.63 (m, 1H, -CONH-), 8.60 (t, *J* = 5.0 Hz, 1H), 8.12 – 8.09 (m, 1H), 7.53 (dd, *J* = 16.6, 8.0 Hz, 3H), 7.43 (dd, *J* = 7.6, 5.1 Hz, 1H), 7.33 – 7.21 (m, 7H), 7.00 (dd, *J* = 11.0, 3.7 Hz, 1H), 4.99 (s, 2H), 4.11 (dd, *J* = 13.5, 8.0 Hz, 1H), 3.23 (dd, *J* = 12.3, 6.2 Hz, 2H), 1.69 – 1.58 (m, 2H), 1.54 – 1.30 (m, 4H). ^{13}C NMR (100MHz, DMSO-d_6) δ 171.82, 165.11, 156.80, 152.16, 149.01, 139.44, 137.36, 135.52, 130.61, 129.23, 128.87, 128.33, 128.26, 123.93, 119.72, 66.00, 55.99, 32.06, 29.31, 23.64. HRMS (ESI) *m/z* calculated for $C_{26}H_{29}N_4O_4^+$ $(M+H)^+$ 461.2189 , found 461.2233.

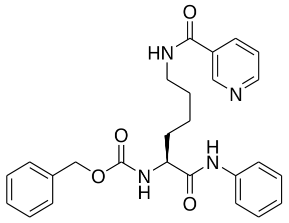

14a

The synthesis of compounds **14b-p, 15a-c and 17a-c** were similar to the synthesis of compound **14a**. The yield of these compounds was about 62%~95%.

(14b): ^{1}H NMR (400 MHz, DMSO-d_6) δ 9.95 (s, 1H, -CONH-), 8.83 (d, *J* = 2.0 Hz, 1H, -CONH-), 8.51 (t, *J* = 5.5 Hz, 1H, -CONH-), 8.00 (dd, *J* = 8.1, 2.3 Hz, 1H), 7.52 (dd, *J* = 17.6, 7.8 Hz, 3H), 7.33 – 7.23 (m, 8H), 7.00 (t, *J* = 7.1 Hz, 1H), 4.99 (s, 2H), 4.10 (dd, *J* = 13.5, 8.2 Hz, 1H), 3.22 (dd, *J* = 12.8, 6.5, 2H), 2.47 (s, 5H), 1.72 – 1.59 (m, 2H), 1.54 – 1.29 (m, 4H). ^{13}C NMR (100 MHz, DMSO-d_6) δ 171.91, 165.26, 161.24, 156.68, 148.67, 139.62, 137.51, 135.63, 129.23, 128.87, 128.33, 128.25, 127.83, 123.14, 119.72, 66.08, 55.83, 31.98, 29.16, 24.48, 23.64. HRMS (ESI) *m/z* calculated for $C_{27}H_{31}N_4O_4^+$ $(M+H)^+$ 475.2345, found 475.2423.

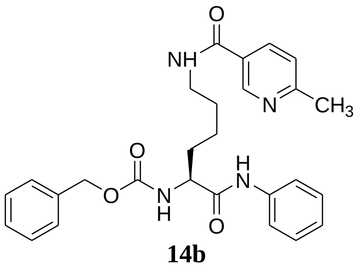

14b

(14c): ^{1}H NMR (400 MHz, DMSO-d_6) δ 9.96 (s, 1H, -CONH-), 8.69 (d, J = 1.5 Hz, 1H, -CONH-),

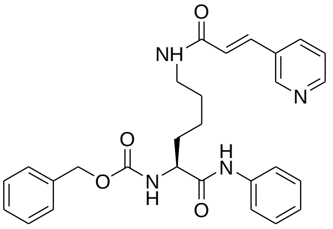

14c

8.50 (dd, J = 4.7, 1.2 Hz, 1H, -CONH-), 8.13 (t, J = 5.5 Hz, 1H), 7.91 (d, J = 8.0 Hz, 1H), 7.55 (d, J = 7.9 Hz, 2H), 7.50 (d, J = 7.8 Hz, 1H), 7.42-7.36 (m, 2H), 7.31 (d, J = 4.3 Hz, 4H), 7.27-7.21 (m, 3H), 6.99 (t, J = 7.4 Hz, 1H), 6.64 (s, 1H), 4.99 (s, 2H), 4.09 (dd, J = 13.3, 8.1 Hz, 1H), 3.13 (dd, J = 12.6, 6.5 Hz, 2H), 1.67 – 1.57 (m, 2H), 1.40 (dd, J = 19.0, 4.6 Hz, 4H). ^{13}C NMR (100 MHz, DMSO-d_6) δ 171.59, 164.99, 156.50, 150.79, 149.22, 141.67, 139.16, 137.26, 131.61, 129.44, 128.76, 128.50, 124.68, 123.73, 119.99, 65.54, 31.49, 29.66, 23.93. HRMS (ESI) m/z calculated for $C_{28}H_{31}N_4O_4^+$ $(M+H)^+$ 487.2345, found 487.2393.

(14d): ^{1}H NMR (400 MHz, CD_3OD) δ 11.32 (s, 1H, -CONH-), 9.99 (d, J = 26.6 Hz, 1H,

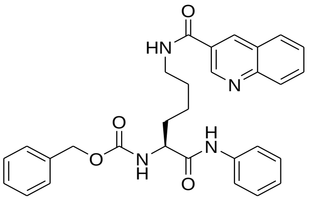

14d

-CONH-), 8.03 – 7.81 (m, 1H, -CONH-), 7.54 (t, J = 9.8 Hz, 3H), 7.38 – 7.09 (m, 7H), 6.97 (dt, J = 10.7, 4.9 Hz, 1H), 6.80 – 6.61 (m, 2H), 6.03 – 5.98 (m, 1H), 5.08 – 4.86 (m, 2H), 4.14 – 3.99 (m, 1H), 3.21 – 3.06 (m, 2H), 1.71 – 1.53 (m, 2H), 1.53 – 1.28 (m, 4H). ^{13}C NMR (100 MHz, CD_3OD) δ 171.54, 161.06, 156.43, 153.37, 149.00, 139.57, 138.20, 129.10, 128.57, 128.25, 126.65, 123.32, 121.42, 119.74, 109.81, 108.74, 65.70, 32.06, 29.31, 23.56. HRMS (ESI) m/z calculated for $C_{30}H_{31}N_4O_4^+$ $(M+H)^+$ 511.2345, found 511.2377.

(14e): ^{1}H NMR (400 MHz, DMSO-d_6) δ 9.95 (s, 1H, -CONH-), 8.09 (dd, J = 10.9, 3.0 Hz, 2H),

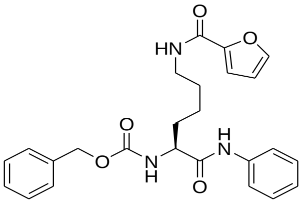

14e

7.64 (t, J = 5.7 Hz, 1H, -CONH-), 7.52 (dd, J = 18.2, 7.8 Hz, 3H), 7.34 – 7.22 (m, 7H), 7.00 (t, J = 7.3, 1H), 6.78 (s, 1H), 4.99 (s, 2H), 4.09 (d, J = 5.2 Hz, 1H), 3.14 (d, J = 5.9 Hz, 2H), 1.69 – 1.58 (m, 2H), 1.40 (ddd, J = 37.4, 19.4, 10.9 Hz, 4H). ^{13}C NMR (100 MHz, DMSO-d_6) δ 171.69, 161.96, 156.57, 145.42, 144.42, 139.56, 137.53, 129.31, 128.87, 128.26, 123.34, 119.72, 109.45, 65.99, 55.94, 38.89, 32.08, 29.63, 23.65. HRMS (ESI) m/z calculated for $C_{25}H_{28}N_3O_5^+$ $(M+H)^+$ 450.2029, found 450.2073.

(14f): ^{1}H NMR (400 MHz, DMSO-d_6) δ 10.01 (s, 1H, -CONH-), 8.28 (t, *J* = 5.4 Hz, 1H, -CONH-), 8.05 (dd, *J* = 12.2, 11.2 Hz, 1H, -CONH-), 7.60 – 7.51 (m, 4H), 7.46 (d, *J* = 5.0 Hz, 1H), 7.31 (dt, *J* = 12.6, 5.9 Hz, 7H), 7.03 (t, *J* = 7.3 Hz, 1H), 5.01 (s, 2H), 4.12 (dd, *J* = 13.3, 8.0 Hz, 1H), 3.20 (dd, *J* = 12.4, 6.3 Hz, 2H), 1.75 – 1.58 (m, 2H), 1.56 – 1.31 (m, 4H). ^{13}C NMR (100 MHz, DMSO-d_6) δ 171.75, 162.54, 156.62, 139.45, 138.49, 137.52, 129.24, 128.88, 128.85, 128.34, 128.27, 127.30, 127.10, 123.77, 119.72, 65.87, 55.92, 32.20, 29.50, 23.82.

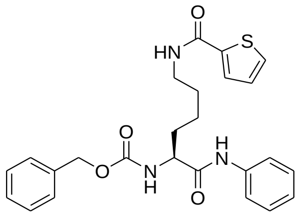

14f

(14h): ^{1}H NMR (400 MHz, DMSO-*d6*) δ 9.99 (d, *J* = 10.0 Hz, 1H, -CONH-), 9.23 (t, *J* = 3.0 Hz, 1H, -CONH-), 8.84 – 8.70 (m, 2H, -CONH-), 8.10 – 7.97 (m, 2H), 7.82 (ddd, *J* = 8.4, 6.9, 1.4 Hz, 1H), 7.71 – 7.63 (m, 1H), 7.63 – 7.44 (m, 3H), 7.41 – 7.10 (m, 7H), 7.06 – 6.92 (m, 1H), 5.13 – 4.85 (m, 2H), 4.18 – 4.06 (m, 1H), 3.40 – 3.20 (m, 6H), 1.82 – 1.52 (m, 4H), 1.50 – 1.33 (m, 2H). ^{13}C NMR (100MHz, DMSO-*d6*) δ 171.42, 165.02, 156.49, 149.51, 148.70, 139.64, 137.51, 135.61, 131.61, 129.72, 129.22, 128.69, 128.39, 127.59, 123.61, 119.86, 65.94, 56.01, 32.26, 29.32, 25.05, 23.72, 22.68. HRMS (ESI) m/z calculated for $C_{25}H_{29}N_4O_4Na^+$ $(M+H)^+$ 471.2008, found 471.2006.

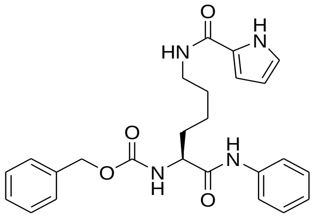

14h

(14i): ^{1}H NMR (400 MHz, DMSO-*d6*) δ 9.96 (s, 1H, -CONH-), 8.93 (s, 1H, -CONH-), 8.66 (s, 1H, -CONH-), 8.45 – 8.37 (m, 2H), 8.11 (d, *J* = 8.8 Hz, 1H), 8.01 (d, *J* = 8.7 Hz, 1H), 7.55 (d, *J* = 8.2 Hz, 4H), 7.25 (dd, *J* = 19.3, 12.1 Hz, 7H), 6.98 (d, *J* = 6.9 Hz, 1H), 4.98 (s, 2H), 4.12 (d, *J* = 5.1 Hz, 1H), 3.30 – 3.26 (m, 2H), 1.65 (d, *J* = 17.3 Hz, 2H), 1.59 – 1.31 (m, 4H). ^{13}C NMR (100 MHz, DMSO-*d6*) δ 171.91, 166.26, 156.85, 152.53, 149.28, 139.56, 137.70, 132.88, 129.44, 129.22, 128.81, 128.32, 128.25, 127.64, 123.77, 122.66, 119.71, 65.95, 55.96, 32.02, 29.36, 23.78. HRMS (ESI) m/z calculated for $C_{30}H_{31}N_4O_4^+$ $(M+H)^+$ 511.2345, found 511.2404.

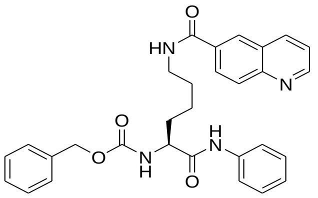

14i

(14j): ^{1}H NMR (400 MHz, DMSO-*d$_6$*) δ 11.50 (s, 1H, -CONH-), 10.02 (d, *J* = 6.2 Hz, 1H, -CONH-), 8.13 (t, *J* = 7.1 Hz, 1H, -CONH-), 8.00 – 7.95 (m, 1H, -CONH-), 7.87 (d, *J* = 5.8 Hz, 1H), 7.58 (d, *J* = 5.9 Hz, 3H), 7.43 – 7.24 (m, 8H), 7.07 (dtd, *J* = 21.8, 14.5, 7.1 Hz, 3H), 5.02 (d, *J* = 6.7 Hz, 2H), 4.18 – 4.09 (m, 1H), 3.24 (d, *J* = 3.9 Hz, 2H), 1.68 (s, 2H), 1.56 – 1.32 (m, 4H). ^{13}C NMR (100 MHz, DMSO-*d$_6$*) δ 171.80, 165.20, 156.79, 139.50, 137.47, 136.52, 129.23, 128.87, 128.32, 128.24, 127.97, 126.65, 123.77, 122.27, 121.58, 120.72, 119.73, 112.19, 111.32, 65.92, 55.94, 32.15, 30.00, 23.64.

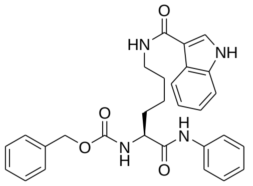

14j

(14k): ^{1}H NMR (400 MHz, DMSO-*d6*) δ 9.99 (d, *J* = 8.0 Hz, 1H, -CONH-), 8.28 (t, *J* = 5.7 Hz, 1H, -CONH-), 7.93 – 7.84 (m, 1H, -CONH-), 7.65 – 7.49 (m, 3H), 7.43 (t, *J* = 7.5 Hz, 1H), 7.41 – 7.06 (m, 10H), 7.04 – 6.95 (m, 1H), 5.13 – 4.86 (m, 2H), 4.20 – 4.02 (m, 1H), 3.66 – 3.51 (m, 1H), 3.46 (s, 2H), 3.37 – 3.27 (m, 4H), 3.27 – 2.99 (m, 3H), 1.79 – 1.56 (m, 2H), 1.56 – 1.32 (m, 4H). ^{13}C NMR (100 MHz, DMSO-*d6*) δ 171.82, 164.66, 156.90, 143.93, 142.26, 139.46, 137.54, 129.23, 128.71, 128.18, 126.49, 125.36, 124.27, 123.78, 122.32, 119.86, 65.94, 55.96, 53.76, 42.94, 31.89, 30.52, 29.15, 23.60. HRMS (ESI) m/z calculated for $C_{30}H_{33}N_3O_4Na^+$ $(M+Na)^+$ 520.2212, found 520.2208.

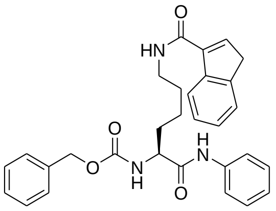

14k

(14m): ^{1}H NMR (400 MHz, DMSO-*d6*) δ 9.97 (s, 1H, -CONH-), 8.50 – 8.38 (m, 1H, -CONH-), 8.34 – 8.19 (m, 1H, -CONH-), 8.02 (dd, *J* = 7.2, 1.0 Hz, 1H), 7.63 – 7.44 (m, 4H), 7.39 – 7.05 (m, 9H), 7.00 (t, *J* = 7.3 Hz, 1H), 5.08 – 4.85 (m, 2H), 4.17 – 4.00 (m, 1H), 3.22 (d, *J* = 5.9 Hz, 2H), 1.77 – 1.26 (m, 6H). ^{13}C NMR (100 MHz, DMSO-*d6*) δ 171.49, 162.69, 156.67, 155.01, 147.87, 139.64, 137.60, 129.06, 128.20, 125.67, 123.96, 122.55, 120.26, 117.13, 112.27, 100.03, 65.84, 56.19, 29.70, 23.73. HRMS (ESI) *m/z* calculated for $C_{29}H_{30}N_3O_5^+$ $(M+H)^+$ 500.2185, found 500.2174 .

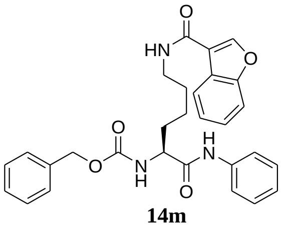

14m

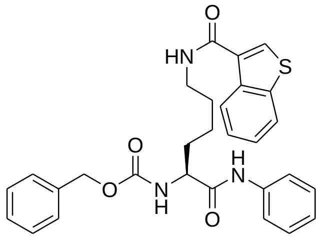

14n

(14n)： ^{1}H NMR (400 MHz, DMSO-*d6*) δ 9.97 (s, 1H, -CONH-), 8.51 – 8.34 (m, 2H), 8.26 – 8.19 (m, 1H, -CONH-), 8.03 – 7.93 (m, 1H), 7.54 (t, *J* = 9.0 Hz, 3H), 7.43 – 7.35 (m, 2H), 7.35 – 7.17 (m, 7H), 7.00 (t, *J* = 7.4 Hz, 1H), 5.12 – 4.89 (m, 2H), 4.19 – 4.03 (m, 1H), 3.22 (ddt, *J* = 20.4, 13.5, 6.9 Hz, 2H), 1.77 – 1.27 (m, 6H). ^{13}C NMR (100 MHz, DMSO-*d6*) δ 171.40, 163.73, 156.05, 140.14, 139.28, 137.56, 132.15, 130.73, 129.33, 128.79, 128.24, 125.34, 123.39, 119.69, 65.65, 55.96, 32.06, 29.22, 23.29. HRMS (ESI) *m/z* calculated for $C_{29}H_{30}N_3O_4S^+$ $(M+H)^+$ 516.1957, found 516.1941.

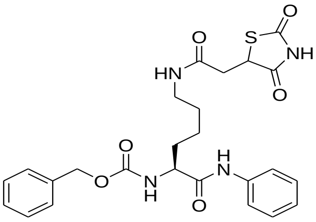

14o

(14o)： ^{1}H NMR (400 MHz, DMSO-*d₆*) δ 11.90 (s, 1H, -CONH-), 9.95 (s, 1H, -CONH-), 8.00 (t, *J* = 4.9 Hz, 1H, -CONH-), 7.51 (dd, *J* = 29.6, 7.8 Hz, 3H), 7.35 – 7.21 (m, 7H), 7.00 (t, *J* = 7.3 Hz, 1H), 4.99 (s, 2H), 4.53 (dt, *J* = 9.2, 3.7 Hz, 1H), 4.08 (dd, *J* = 13.5, 8.1 Hz, 1H), 2.97 (t, *J* = 11.8 Hz, 2H), 2.94 (s, 1H), 2.75 – 2.66 (m, 1H), 1.70 – 1.53 (m, 2H), 1.31 (d, *J* = 38.8 Hz, 4H). ^{13}C NMR (100 MHz, DMSO-d_6) δ 176.53, 173.47, 171.60, 168.77, 156.74, 139.41, 137.51, 129.23, 128.87, 128.34, 128.27, 123.80, 119.80, 65.93, 55.78, 47.55, 37.54, 32.00, 29.21, 23.42. HRMS (ESI) *m/z* calculated for $C_{25}H_{29}N_4O_6S^+$ $(M+H)^+$ 513.1808, found 513.1883.

(14p): ^{1}H NMR (400 MHz, DMSO-*d6*) δ 10.76 (d, *J* = 17.1 Hz, 1H, -CONH-), 10.00 (d, *J* = 18.7 Hz, 1H, -CONH-), 7.94 – 7.81 (m, 1H, -CONH-), 7.63 – 7.48 (m, 4H), 7.28 (ddd, *J* = 21.2, 14.8, 7.1 Hz, 8H), 6.99 (ddd, *J* = 28.8, 27.1, 11.8 Hz, 4H), 6.67 (dd, J = 18.5, 8.1 Hz, 1H), 4.98 (t, *J* = 15.7 Hz, 2H), 4.11 (s, 2H), 3.00 (d, *J* = 14.0 Hz, 3H), 2.91 – 2.79 (m, 1H), 1.62 (s, 2H), 1.46 – 1.21 (m, 13H). ^{13}C NMR (100 MHz, DMSO-*d6*) δ 172.29, 171.69, 156.69, 155.58, 139.59, 137.63, 136.56, 129.24, 129.20, 128.86, 128.82, 128.27, 128.23, 127.85, 124.20, 123.79, 121.37, 119.69, 119.06, 118.59, 111.69, 110.71, 78.45, 65.85, 56.00, 55.67, 31.97, 29.19, 28.67, 28.62, 28.52, 28.47, 28.20, 23.34. HRMS (ESI) *m/z* calculated for $C_{36}H_{44}N_5O_6^+$ $(M+H)^+$ 642.3292, found 642.3389.

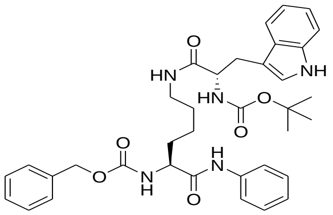

14p

(14q): The compound **14p** (100mg, 0.155mmol) was added in anhydrous CH_2Cl_2 (4mL) and cooled (0°C), and then the TFA (2mL) was added slowly, stirred for 2h. Then, the TFA was removed under reduced pressure until there was no TFA, the residue was purified by silica gel column chromatography (50mg, 60%). ^1H NMR(400MHz, DMSO-*d6*) δ 10.79 (s, 1H, -CONH-), 9.98 (s, 1H, -CONH-), 7.83 (t, *J* = 5.5 Hz, 1H, -CONH-), 7.58 (d, *J* = 7.9 Hz, 2H), 7.52 (t, *J* = 6.8 Hz, 2H), 7.34 – 7.24 (m, 8H), 7.11 (s, 1H), 7.02 (t, *J* = 6.9 Hz, 2H), 6.94 (t, *J* = 7.1 Hz, 1H), 5.01 (s, 2H), 4.10 (d, *J* = 5.3 Hz, 1H), 3.40 (dd, *J* = 8.1, 4.7 Hz, 1H), 3.06 – 2.98 (m, 3H), 2.70 (dd, *J* = 14.1, 8.3 Hz, 1H), 1.62 (d, *J* = 5.4 Hz, 2H), 1.32 (dd, *J* = 18.2, 9.9 Hz, 4H). ^{13}C NMR (100 MHz, DMSO-*d6*) δ 174.89, 171.58, 156.87, 139.38, 137.76, 136.37, 128.62, 124.18, 123.63, 121.12, 119.44, 118.90, 111.95, 111.12, 65.90, 55.96, 33.22, 31.54, 29.87, 23.23, 22.67, 20.76. HRMS (ESI) *m/z* calculated for $C_{31}H_{36}N_5O_4^+$ $(M+H)^+$ 542.2767, found 542.2854.

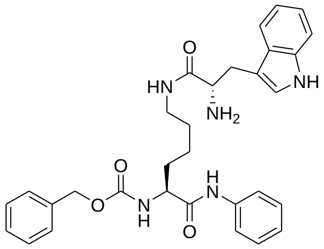

14q

(15a): ^1H NMR (400 MHz, CD_3OD) δ 7.49 (dd, *J* = 24.8, 10.2 Hz, 2H), 7.36 – 7.14 (m, 7H), 7.07 (t, *J* = 7.3 Hz, 1H), 5.11 – 5.02 (m, 2H), 4.23 – 4.11 (m, 1H), 3.70 – 3.62 (m, 4H), 3.28 (dd, *J* = 3.1, 1.5 Hz,1H), 3.19 (dq, *J* = 12.2, 6.3 Hz, 2H), 1.86 – 1.61 (m, 2H), 1.57 – 1.34 (m,4H). ^{13}C NMR (100 MHz, CD_3OD) δ 173.56, 172.20, 168.66, 167.30, 157.45, 138.33, 136.68, 128.47, 128.19, 127.12, 123.82, 120.27, 66.18, 55.79, 51.43, 38.87, 31.77, 30.95, 27.94, 23.06. HRMS (ESI) *m/z* calculated for $C_{24}H_{30}N_3O_6Na^+$ $(M+Na)^+$ 478.1954, found 478.1978.

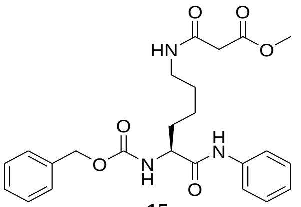

15a

(15b): ^1H NMR (400 MHz, DMSO-*d6*) δ 9.95 (s, 1H, -CONH-), 7.99 – 7.86 (m, 1H, -CONH-), 7.69 (d, *J* = 16.8 Hz, 1H, -CONH-), 7.52 (dd, *J* = 28.1, 6.3 Hz, 2H), 7.38 – 7.14 (m, 7H), 7.00 (t, *J* = 6.7 Hz, 1H), 4.97 (d, *J* = 14.1 Hz, 2H), 4.03 (dd, *J* = 24.4, 20.6 Hz, 2H), 3.62 – 3.49 (m, 3H), 2.97 (s, 2H), 2.23 (d, *J* = 3.2 Hz, 2H), 2.03 – 1.95 (m, 2H), 1.71 – 1.52 (m, 2H), 1.47 – 1.26 (m, 8H). HRMS (ESI) *m/z* calculated for $C_{27}H_{36}N_3O_6^+$ $(M+H)^+$ 498.2604, found 498.2583.

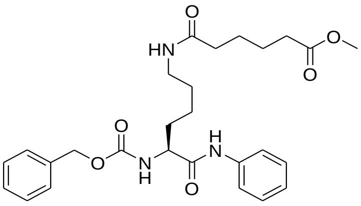

15b

(15c): ^{1}H NMR (400 MHz, CD_3OD) δ 7.49 (t, *J* = 20.2 Hz, 2H), 7.42 – 7.13 (m, 7H), 7.05 (dd, *J* = 17.3, 10.3 Hz, 1H), 5.19 – 4.94 (m, 2H), 4.30 – 4.08 (m, 1H), 3.61 (s, 3H), 3.13 (t, *J* = 6.0 Hz,2H), 2.26 (t, *J* = 7.3 Hz, 2H), 2.18 – 2.03 (m, 2H), 1.92 – 1.60 (m, 2H), 1.60 (s, 8H), 1.28 (d, *J* = 19.5 Hz, 4H). ^{13}C NMR (100 MHz, CD_3OD) δ 174.67, 172.11, 157.10, 138.00, 136.98, 128.68, 128.19, 127.71, 123.90, 119.68, 66.39, 55.63, 50.86, 38.27, 35.51, 33.50, 28.73, 25.74, 24.51, 22.98. HRMS (ESI) *m/z* calculated for $C_{29}H_{39}N_3O_6Na^+$ $(M+Na)^+$ 548.2737, found 548.2731.

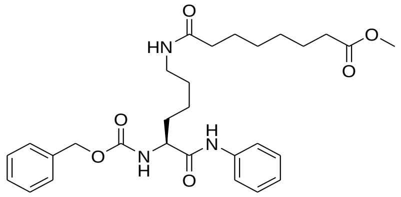

15c

(16a): The compound **15a** (150mg, 0.32mmol) was added in THF:H_2O=3:1 (4mL) and cooled (0°C), and then the LiOH（THF:H_2O=3:1, 0.5mL) was added slowly, stirred for 30min. Then, added in 10% Citric acid solution , the reaction mixture was diluted with CH_2Cl_2 and saturated NaCl solution, the organic layer was washed with saturated NaCl, and dried over Na_2SO_4 and concentrated. The residue was purified by silica gel column chromatography (110mg, 78%). ^{1}H NMR (400 MHz, CD_3OD) δ 7.50 (t, *J* = 11.5 Hz, 2H), 7.39 – 7.12 (m, 7H), 7.07 (t, *J* = 6.9 Hz, 1H), 5.14 – 5.01 (m,2H), 4.16 (ddd, *J* = 30.7, 8.3, 4.5 Hz, 1H), 3.34 – 3.27 (m, 1H), 3.19 (d, *J* = 6.8 Hz, 3H), 1.87 – 1.63 (m, 2H), 1.60 – 1.36 (m, 4H). ^{13}C NMR (100 MHz, CD_3OD) δ 171.98, 170.28, 167.75, 162.43, 157.41, 138.14, 136.68, 128.58, 128.28, 127.50, 124.09, 120.16, 66.45, 55.52, 38.77, 31.46, 28.14, 23.07. HRMS (ESI) *m/z* calculated for $C_{23}H_{28}N_3O_6^+$ $(M+H)^+$ 442.1978, found 442.1961.

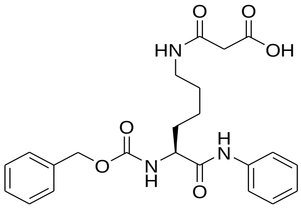

16a

The synthesis of compounds **16b-c** were similar to the synthesis of compound **16a**. The yield of these compounds was about 70%~85%.

(16b): ^{1}H NMR (400 MHz, CD_3OD) δ 7.62 – 7.41 (m, 2H), 7.39 – 7.12 (m, 7H), 7.06 (dd, *J* = 16.2, 8.8 Hz, 1H), 5.19 – 4.96 (m, 2H), 4.25 – 4.06 (m, 1H), 3.21 – 3.08 (m, 2H), 2.33 – 2.18 (m, 2H), 2.18 (s, 2H), 1.88 – 1.63 (m, 2H), 1.63 – 1.33 (m, 8H). ^{13}C NMR (100 MHz, CD_3OD) δ 175.73, 174.31, 138.13, 137.02, 128.66, 124.39,

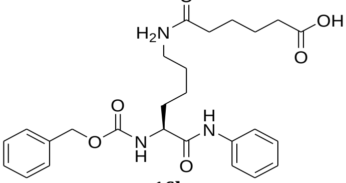

16b

120.36, 66.47, 55.57, 38.36, 35.45, 33.46, 31.49, 29.19, 25.41, 23.99, 22.87. HRMS (ESI) *m/z* calculated for $C_{26}H_{34}N_3O_6^+$ (M+H)$^+$ 484.2448, found 484.2426.

(16c): ^{1}H NMR (400 MHz, CD_3OD) δ 7.62 – 7.41 (m, 2H), 7.40 – 7.12 (m,7H), 7.11 – 7.01 (m, 1H), 5.18 – 4.99 (m, 2H), 4.24 – 4.08 (m, 1H), 3.18 – 3.10 (m, 2H), 2.27 – 2.20 (m, 2H), 2.11 (t, *J* = 7.5 Hz, 2H), 1.87 – 1.65 (m, 2H), 1.60 – 1.36 (m, 8H), 1.34 – 1.18 (m, 6H).

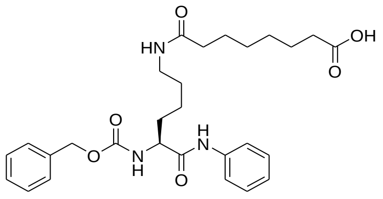

16c

^{13}C NMR (100 MHz, CD_3OD) δ 176.06, 174.91, 172.07, 157.09, 138.36, 136.93, 128.53, 127.66, 123.94, 120.19, 66.65, 55.95, 38.72, 37.85, 35.54, 33.81, 28.58, 25.16, 23.98, 22.85. HRMS (ESI) *m/z* calculated for $C_{28}H_{38}N_3O_6^+$ (M+H)$^+$ 512.2761, found 512.2750.

(17a): ^{1}H NMR (400 MHz, CD_3OD) δ 7.65 – 7.41 (m, 3H), 7.40 – 7.15 (m, 7H), 7.12 – 6.96 (m, 2H), 6.81 (dt, *J* = 8.0, 1.8 Hz, 1H), 6.67 (td, *J* = 7.6, 1.3 Hz, 1H), 5.15 – 4.99 (m, 2H), 4.18 (dd, *J* = 19.3, 13.9 Hz,1H), 3.28 (dt, *J* = 3.2, 1.6 Hz, 2H), 3.24 – 3.11 (m, 2H), 1.91 – 1.63 (m, 2H), 1.61 – 1.36 (m, 4H). ^{13}C NMR (100MHz, CD_3OD) δ 176.03, 168.23, 138.26, 127.93, 127.07, 125.93, 124.29, 123.15, 120.07, 118.12, 116.98, 66.63, 65.17, 33.87, 31.32, 30.48, 29.64, 28.79, 28.55, 24.92, 22.67, 22.08. HRMS (ESI) *m/z* calculated for $C_{29}H_{34}N_5O_5^+$ (M+H)$^+$ 532.2560, found 532.2545.

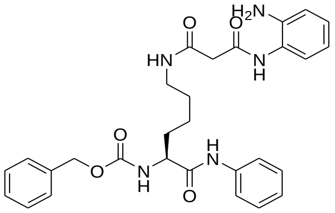

17a

(17b): ^{1}H NMR (400 MHz, CD_3OD) δ 7.58 – 7.42 (m, 2H), 7.40 – 7.11 (m, 7H), 7.11 – 7.01 (m, 2H), 7.01 (s, 1H), 6.80 (dt, J = 10.8, 5.4 Hz, 1H), 6.68 (ddt, *J* = 19.1, 13.0, 6.5 Hz, 1H), 5.17 – 4.96 (m, 2H), 4.19 (dt, *J* = 23.7, 11.8 Hz, 1H), 3.22 – 3.04 (m, 2H), 2.48 – 2.32 (m, 2H), 2.25 – 2.10 (m, 2H), 1.89 – 1.60 (m, 6H), 1.46 (dddd, *J* = 10.6, 10.1, 8.4, 5.3 Hz, 2H). ^{13}C NMR (100 MHz, CD_3OD) δ 174.64, 173.25, 172.11, 157.00, 141.57, 137.96, 136.85, 128.46, 127.07, 126.76, 125.61, 123.98, 119.75, 118.37, 117.22, 66.32, 55.69, 38.33, 35.26, 34.10, 31.32, 30.77, 29.32, 28.54, 25.16, 23.22, 22.62. HRMS (ESI) m/z calculated for $C_{32}H_{40}N_5O_5Na^+$ (M+Na)$^+$ 596.2849, found 596.2839.

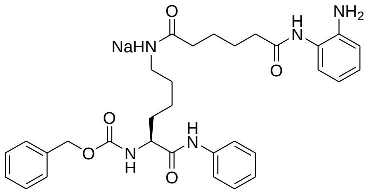

17b

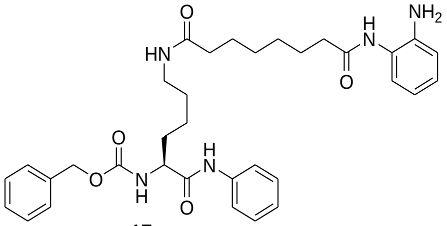

17c

(17c): ^{1}H NMR (400 MHz, CD_3OD) δ 7.61 – 7.41 (m, 2H), 7.42 – 7.11 (m, 7H), 7.10 – 6.94 (m, 3H), 6.80 (dt, *J* = 10.8, 5.4 Hz, 1H), 6.71 – 6.64 (m, 1H), 5.07 (q, *J* = 12.6 Hz, 2H), 4.19 (dd, *J* = 8.4, 5.5 Hz, 1H), 3.20 – 3.04 (m, 2H), 2.39 (dt, *J* = 14.9, 7.5 Hz, 2H), 2.10 (dt, *J* = 21.1, 7.2 Hz, 2H), 1.88 – 1.44 (m, 8H), 1.43 – 1.29 (m, 6H). ^{13}C NMR (100 MHz, CD_3OD) δ 175.12, 173.96, 142.07, 137.86, 127.70, 127.13, 125.67, 124.02, 120.06, 118.04, 117.19, 66.68, 38.45, 35.35, 31.63, 29.14, 28.28, 25.72, 22.92. HRMS (ESI) *m/z* calculated for $C_{34}H_{43}N_5O_5Na^+$ $(M+Na)^+$ 624.3162, found 624.3138 .

The synthesis of compounds **18b-c** were similar to the synthesis of compound **11**. The yield of these compounds was about 42%.

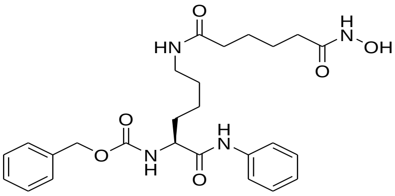

18b

(18b): ^{1}H NMR (400 MHz, CD_3OD) δ 7.58 – 7.44 (m, 2H), 7.39 – 7.12 (m, 7H), 7.06 (dd, *J* = 16.3, 8.9 Hz, 1H), 5.19 – 4.97 (m, 2H), 4.27 – 4.10 (m, 1H), 3.16 (p, *J* = 7.4 Hz, 2H), 2.20 – 1.99 (m, 4H), 1.88 – 1.63 (m, 2H), 1.63 – 1.35 (m, 8H). ^{13}C NMR (100MHz, CD_3OD) δ 174.48, 172.15, 171.28, 157.30, 138.04, 136.92, 128.17, 124.39, 120.30, 66.39, 55.63, 38.45, 35.25, 32.33, 28.83, 24.73, 23.28. HRMS (ESI) m/z calculated for $C_{26}H_{35}N_4O_6^+$ $(M+H)^+$ 499.2557, found 499.2554.

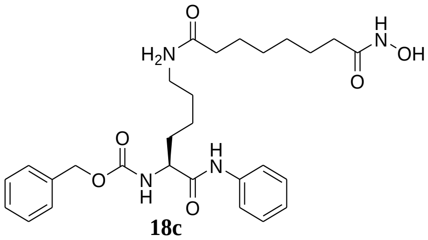

18c

(18c): ^{1}H NMR (400 MHz, CD_3OD) δ 7.62 – 7.43 (m, 2H), 7.37 – 7.13 (m, 7H), 7.12 – 7.01 (m, 1H), 5.15 – 4.96 (m, 2H), 4.27 – 4.10 (m, 1H), 3.21 – 3.08 (m, 6H), 2.18 – 2.00 (m, 4H), 1.87 – 1.63 (m, 2H), 1.61 – 1.34 (m, 8H). ^{13}C NMR (100 MHz, CD_3OD) δ 174.59, 172.08, 157.11, 138.26, 136.85, 127.84, 123.89, 119.63, 66.38, 55.43, 38.75, 35.41, 32.33, 31.75, 30.36, 29.21, 28.10, 25.24, 23.00. HRMS (ESI) *m/z* calculated for $C_{28}H_{40}N_4O_6^+$ $(M+H)^+$ 527.2870, found 527.2854 .

HDACS inhibition assay in vitro

In vitro HDACs inhibition assays were conducted as previously described[1]. In brief, 4 μL of HeLa nuclear extract (9mg/mL) was mixed with various concentrations of tested compounds (1μL), then fluorogenic substrate Boc-Lys(acetyl)-AMC (100 μM) and Buffer A (50 mM Tis-HCl pH=8.0, 150 mM NaCl, 30 mM KCl, 10 mM $MgCl_2$) were added. After incubation at 37℃ for 1h, the mixture was stopped by the addition of 50 μL of developer containing trypsin and 1μM SAHA. And then incubation at 37℃ for 1h, the mixture was stopped by the addition of 50 μL of Buffer B (200mM HCl/300 mM Acetic acid), fluorescence intensity was measured using a microplate reader at excitation and emission wavelengths of 360 nm and 460 nm. The inhibition ratios were calculated from the fluorescence intensity readings of tested wells relative to those of control wells, and the IC_{50} values (Concentrations of tested compounds **SAHA:** 500 nM, 100 nM, 50 nM, 25 nM, 12.5 nM, 6.25 nM; **11:** 100 μM, 75 μM, 50 μM, 25 μM, 12.5 μM; **18b**: 50 μM, 10 μM, 1μM, 500 nM, 100 nM, 50nM; **18c**: 50 μM, 10 μM, 1 μM, 500 nM, 100 nM, 50nM) were calculated using a regression analysis of the concentration/inhibition data. All experiments were done in triplicate.

Table S1. Selective investigation of Nε-acetyl lysine derivatives as HDAC inhibitors.

Inhibitor	HDAC Inhibition (%)		
	Hela nuclear extract		
	HDACI	HDACIIa	HDAC8
**SAHA**	87.05±2.193	15.51±3.084	41.19±0.180
**18c**	61.06±1.064	16.65±2.690	38.27±1.215

*1μM

Cell Proliferation Assay

The rate of cell survival under the action of test substances was evaluated by an improved MTS assay as previously described[2]. In brief, A549 (5000 cells / well) , HepG2 (5000 cells / well), HEK293 (5000 cells / well) and K562 (1×10^4 cells/well) cell lines were seeded into 96-well plates in 100 μL medium. After overnight incubation, each well was added with different concentrations of the compounds **SAHA, 16c** and **18c**, then incubated for another 48 hours. After 48 hours 5ul of MTS reagent solution was added into each well and the cells were incubated for another 4 hours at 37℃ under 5% CO_2 environment. The absorbance was measured at 490 nm in

a Microplate to determine the cell viability. The absorbance was directly proportional to the number of viable cells. All experiments were done in triplicate.

Table S2. The IC_{50}s of **18c** and **SAHA** against both tumor cells and normal cells, then calculate and compare their selective index.

	K562	A549	HepG2	HEK293	selectivity index(SI)
**SAHA**	4.58±0.17	17.81±1.25	4.23±0.63	6.09±0.84	0.34~1.43
**18c**	41.18±1.73	134.10±2.13	158.40±2.20	>500	3.16~12.14
**16c**	>500	>500	>500	>500	/

Western Blot Methods

Respectively, A549 and K562 cells were harvested with three different concentrations of compound **18c** (50 μM, 100 μM, 200 μM) and concentrations of **SAHA** (10 μM) under 8 mL DMEM with 10% FBS (fetal bovine serum) for 24 hours. DMSO was used as the control. Later the cell pellets were collected by centrifuge at 1000 rpm for 5 minutes and then washed by ice-cold PBS (phosphate-buffered saline) twice. The PBS solution was drained and the cell pellets were re-suspended with ice-cold cell lysate buffer (50 mM Tis-HCl PH=7.4, 150 mM NaCl, 1 mM EDTA, 10% glycerin, 1% tirtion 100 and protease inhibitors cocktail) and Vortex blending five times for 30 seconds each over a period of 30 minutes kept on ice. The samples were later centrifuged at 12,000×g for 10 minutes at 4 ˚C and the supernatants in each tube were collected and placed in a fresh tube kept on ice. BCA (bicinchoninic acid) assay was used to determine the total protein amount. SDS polyacrylamide gel electrophoresis (PAGE) was added into each sample and the mixture was boiled at 100°C for 10 minutes for denaturation. Around 60 µg protein was loaded into each well and the gels will be submerged in migration buffer which normally contains 25 mM Tris base, 190 mM glycine and 0.1% SDS. Run the gel for 20 minutes under 80 V/30 minutes under 120 V/25 minutes under 200 V. The proteins were then immobilized on a nitrocellulose membrane following electrophoretic transfer from the gel at 60 minutes under 100 V/350mA at 4 ˚C via wet transfer process. Non-protein binding areas on the membrane were blocked to prevent non-specific binding of antibodies by 5% BSA in TBST (10 mM Tris, PH=7.4, 150 mM NaCl and 0.5% Tween 20) at room temperature for 2 hour and the membranes were incubated with primary antibodies respectively (acetyl-α-Tubulin, sc-23950, Santa; anti-α-Tubulin, cat:M1000130, Solarbio) that specifically bound to the protein of interest. Unbound antibodies

were removed by washing and a secondary antibody conjugated to an enzyme, a fluorophore was used for detection. The detected signal from the protein: antibody: antibody complex was proportional to the amount of protein on the membrane. Later the membranes were striped and re-probed with loading control primary antibodies respectively (anti-α-Tubulin, acetyl-α-Tubulin) and repeated the previous steps to detect the signal.

Supplementary references:

1. Xiaoyang Li, Yuri K. Peterson, Elizabeth S. Inks, Richard A. Himes, Jiaying Li, Yingjie Zhang, Xiujie Kong, and C. James Chou, *Journal of Medicinal Chemistry*, 2018, **61**, 2589-2603.
2. Jinyu Yang, Gaoliang Cheng, Qihao Xu, Shenglin Luan, Shuxiang Wang, Dan Liu, Linxiang Zhao, *Bioorganic & Medicinal Chemistry*, 2018, **26**, 1418-1425.
3. Raffaella, Cincinelli, Vincent Zwick, Loana Musso, Valentina Zuco, Michelandrea De Cesare, Franco Zunino, Claudia Simoes-Pires, Alessandra Nurisso, Giuseppe Giannini, Muriel Cuendet, Sabrina Dallavalle, *European Journal of Medicinal Chemistry*, 2016, **112**, 99-105.
4. Xiaoyang Li, Elizabeth S. Inks, Xiaoguang Li, Jinning Hou, C. James Chou, Jian Zhang, Yuqi Jiang,Yingjie Zhang, and Wenfang Xu, *Journal of Medicinal Chemistry*, 2014, **57**, 3324-3341.

CD$_3$OD

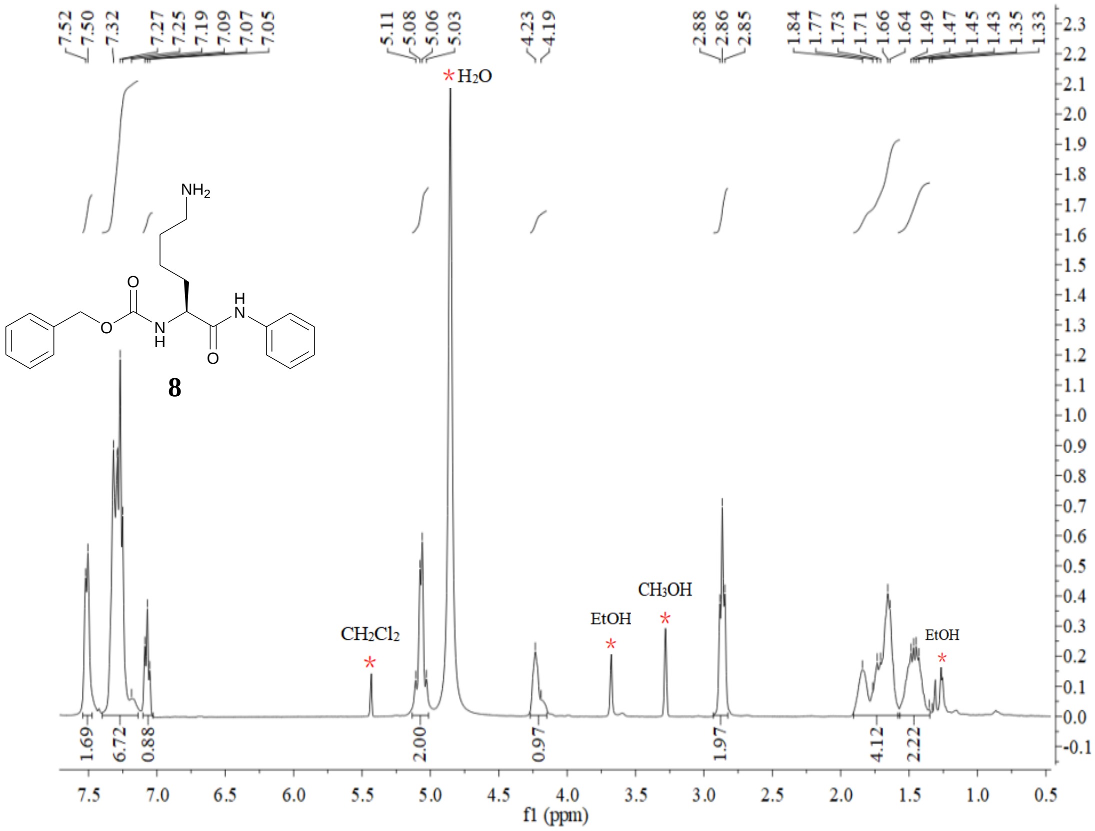

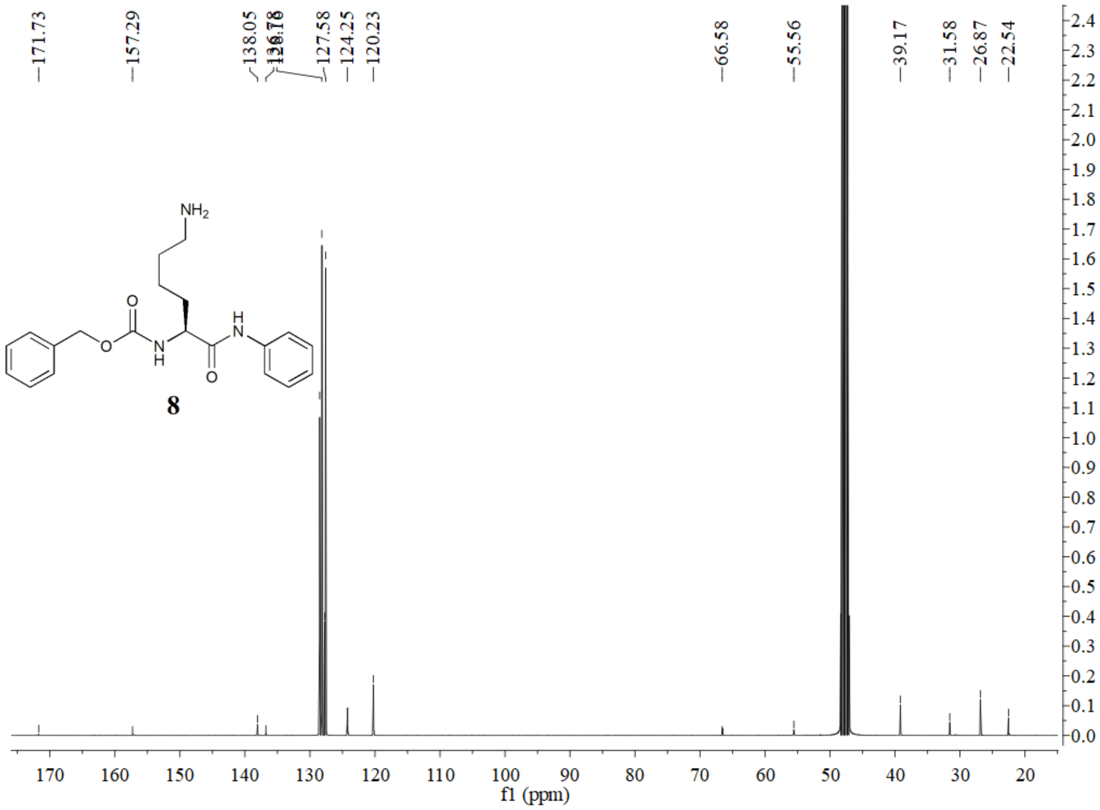

DMSO-d_6

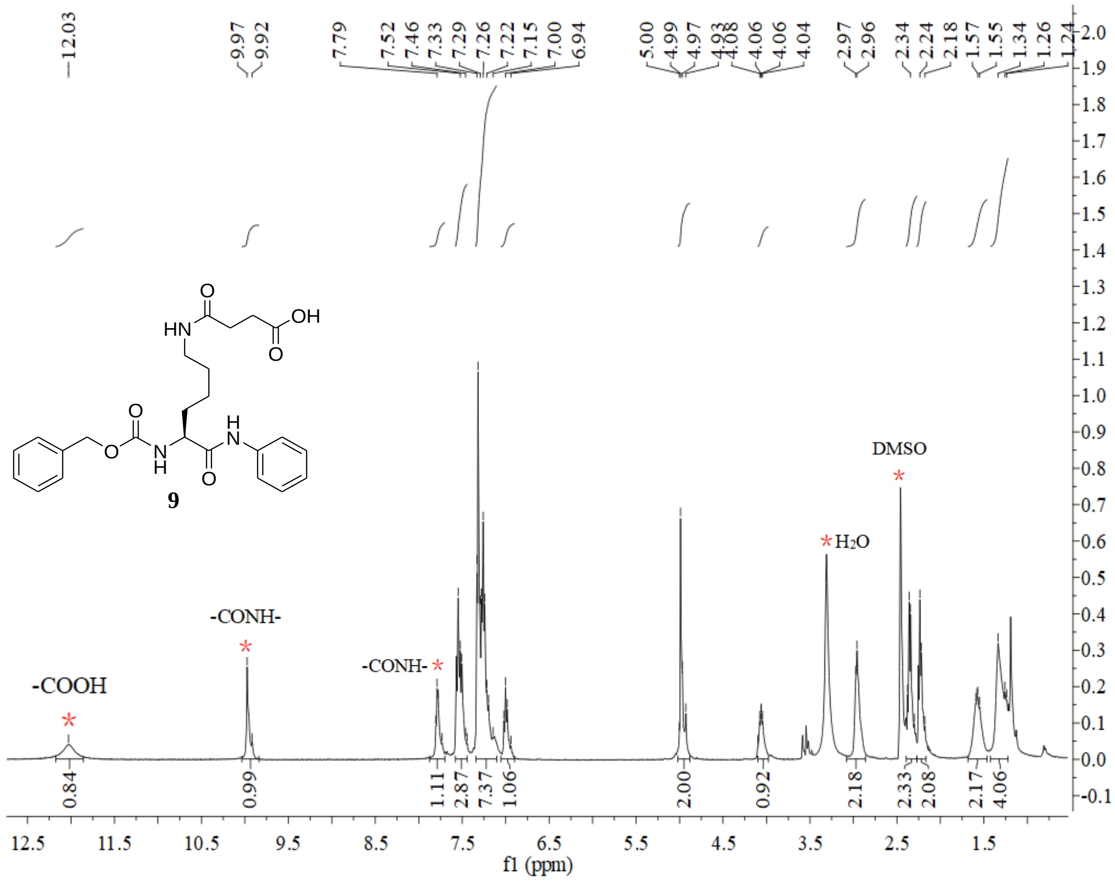

12.03
9.97
9.92
7.79
7.52
7.46
7.33
7.29
7.26
7.22
7.15
7.00
6.94
5.00
4.99
4.97
4.93
4.08
4.06
4.06
4.04
2.97
2.96
2.34
2.24
2.18
1.57
1.55
1.34
1.26
1.24
-COOH
-CONH-
-CONH-
DMSO
H2O
9
0.84
0.99
1.11
2.87
7.37
1.06
2.00
0.92
2.18
2.33
2.08
2.17
4.06
f1 (ppm)

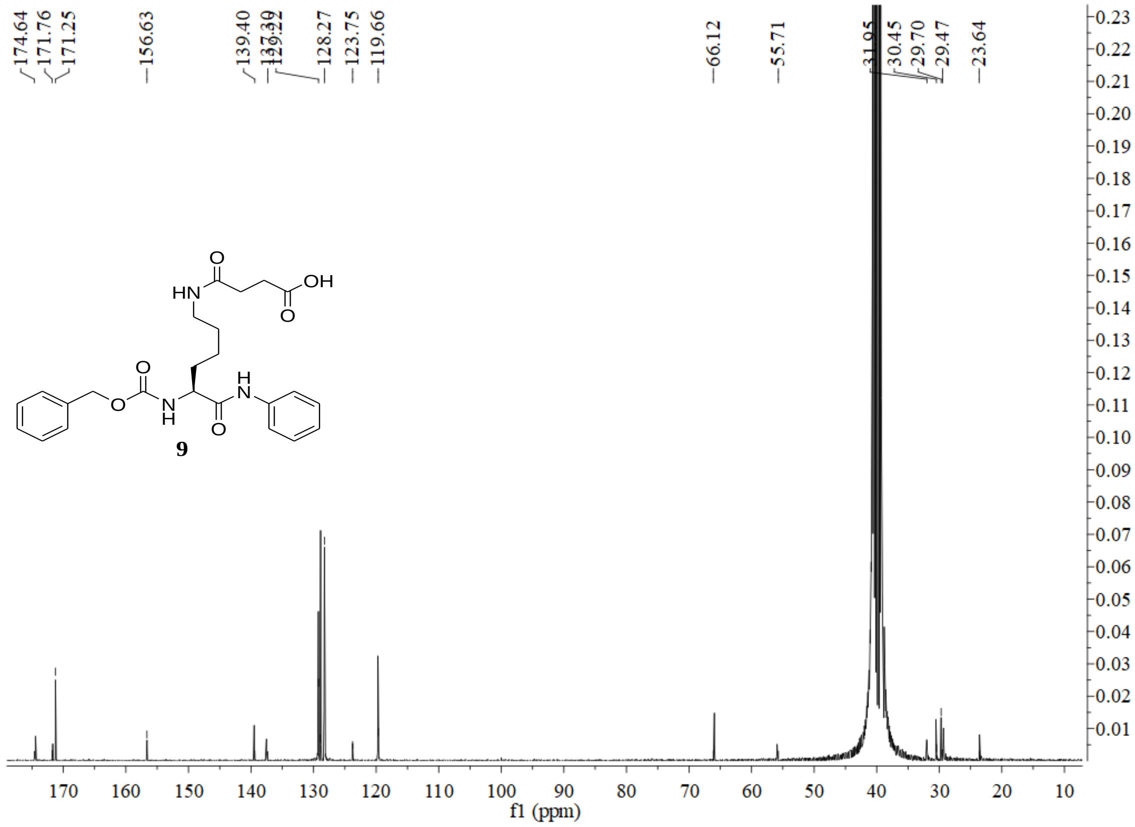

174.64
171.76
171.25
156.63
139.40
137.39
129.22
128.27
123.75
119.66
66.12
55.71
31.95
30.45
29.70
29.47
23.64
9
f1 (ppm)

DMSO-d_6

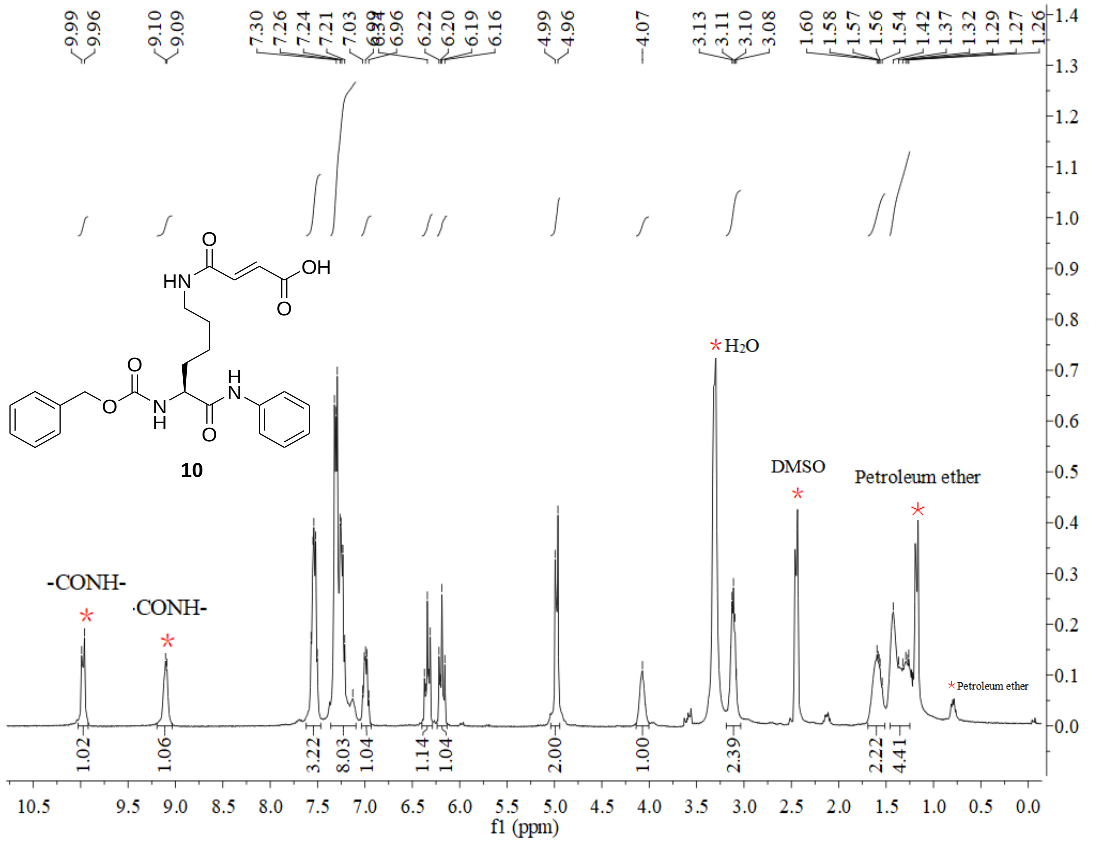

9.99
9.96
9.10
9.09
7.30
7.26
7.24
7.21
7.03
6.96
6.22
6.20
6.19
6.16
4.99
4.96
4.07
3.13
3.11
3.10
3.08
1.60
1.58
1.57
1.56
1.54
1.42
1.37
1.32
1.29
1.27
1.26
10
-CONH-
·CONH-
*H2O
DMSO
Petroleum ether
*Petroleum ether
1.02
1.06
3.22
8.03
1.04
1.14
1.04
2.00
1.00
2.39
2.22
4.41
f1 (ppm)

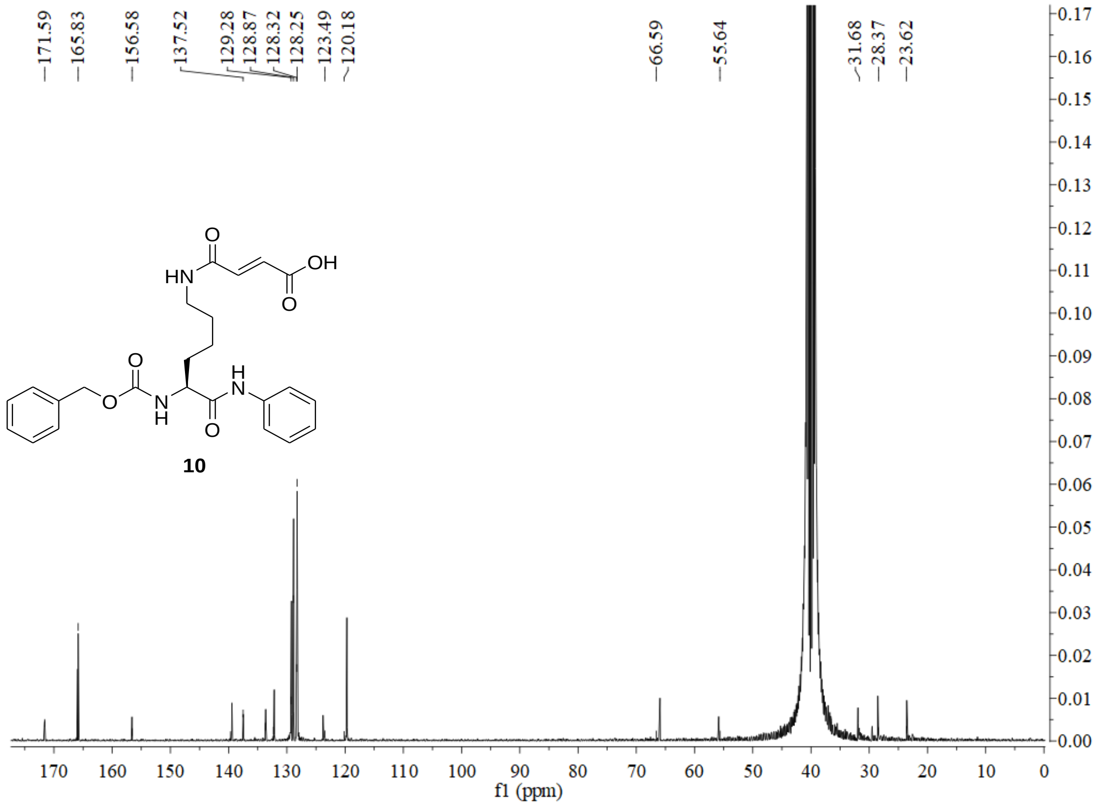

171.59
165.83
156.58
137.52
129.28
128.87
128.32
128.25
123.49
120.18
66.59
55.64
31.68
28.37
23.62
10
f1 (ppm)

CD$_3$OD

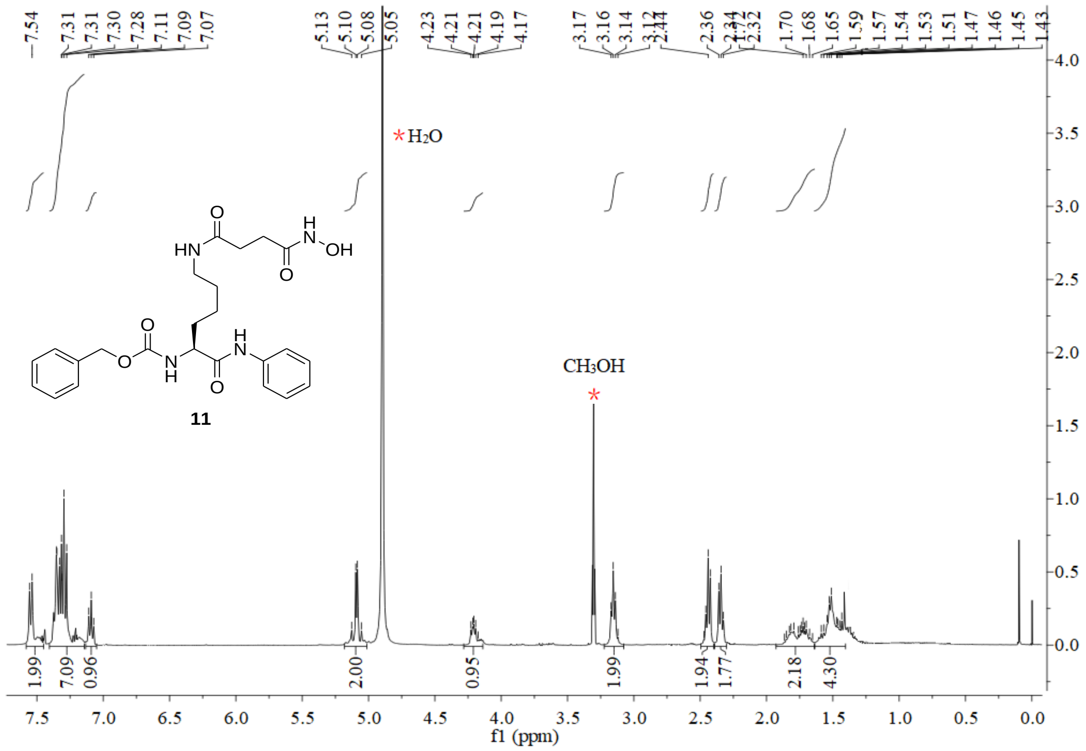

7.54
7.31
7.31
7.30
7.28
7.11
7.09
7.07
5.13
5.10
5.08
5.05
4.23
4.21
4.21
4.19
4.17
3.17
3.16
3.14
2.36
2.32
1.70
1.68
1.65
1.59
1.57
1.54
1.53
1.51
1.47
1.46
1.45
1.43
*H2O
CH3OH
11
1.99
7.09
0.96
2.00
0.95
1.99
1.94
1.77
2.18
4.30
f1 (ppm)

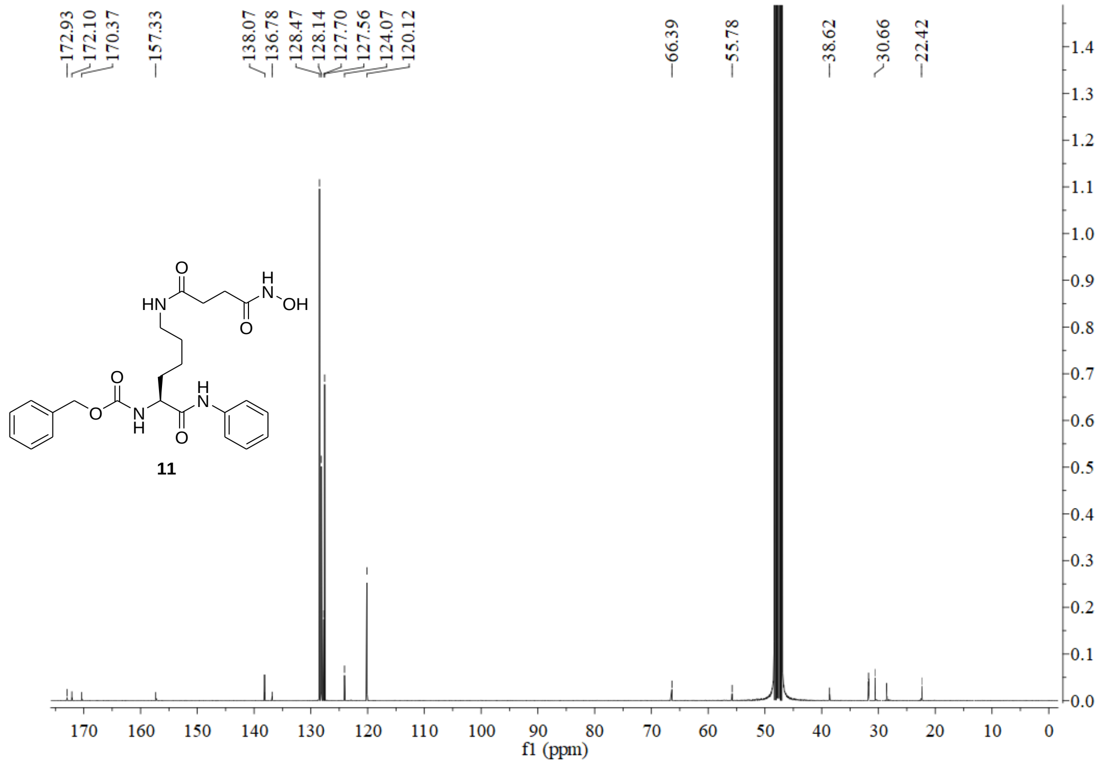

172.93
172.10
170.37
157.33
138.07
136.78
128.47
128.14
127.70
127.56
124.07
120.12
66.39
55.78
38.62
30.66
22.42
11
f1 (ppm)

DMSO-d_6

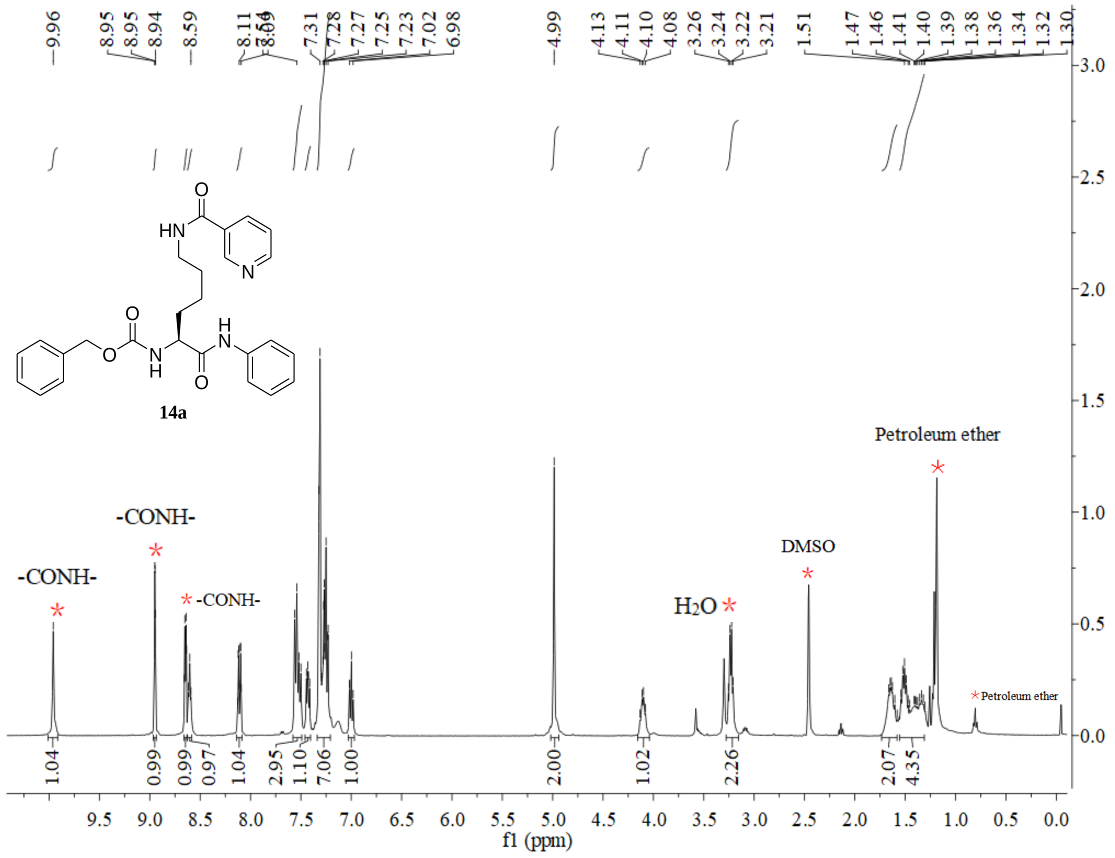

9.96
8.95
8.95
8.94
8.59
8.11
7.54
8.09
7.31
7.28
7.27
7.25
7.23
7.02
6.98
4.99
4.13
4.11
4.10
4.08
3.26
3.24
3.22
3.21
1.51
1.47
1.46
1.41
1.40
1.39
1.38
1.36
1.34
1.32
1.30
14a
-CONH-
-CONH-
-CONH-
H2O
DMSO
Petroleum ether
Petroleum ether
f1 (ppm)

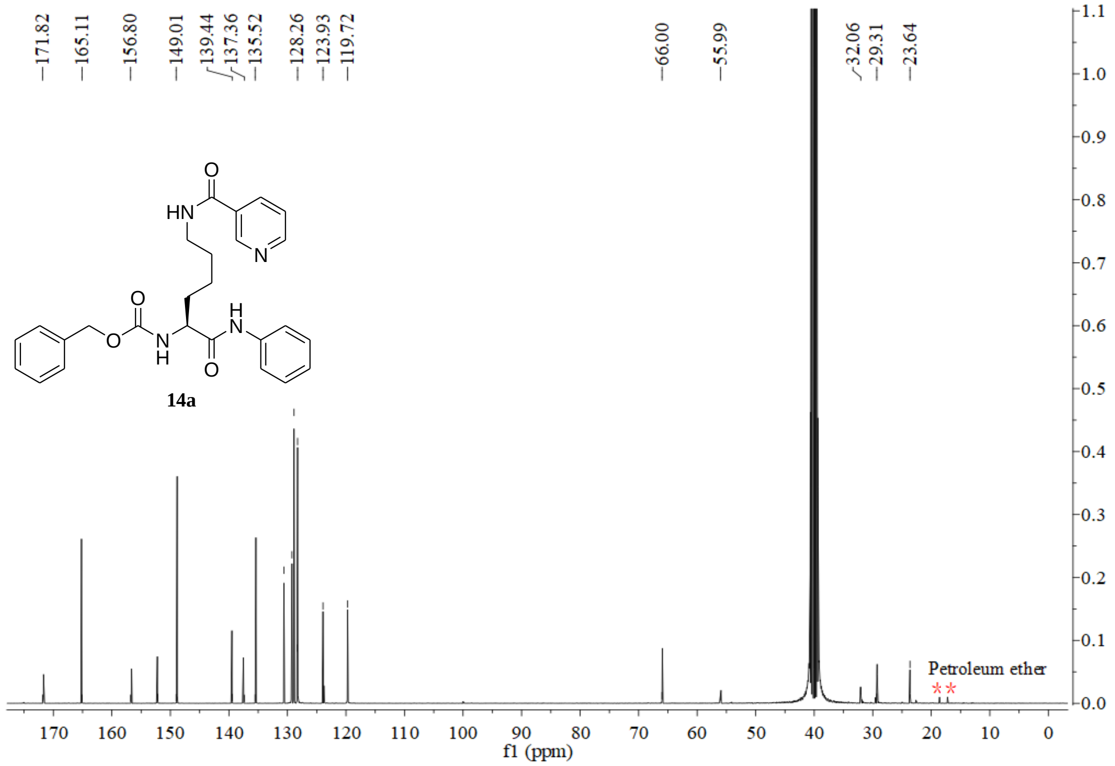

171.82
165.11
156.80
149.01
139.44
137.36
135.52
128.26
123.93
119.72
66.00
55.99
32.06
29.31
23.64
14a
Petroleum ether
f1 (ppm)

DMSO-d_6

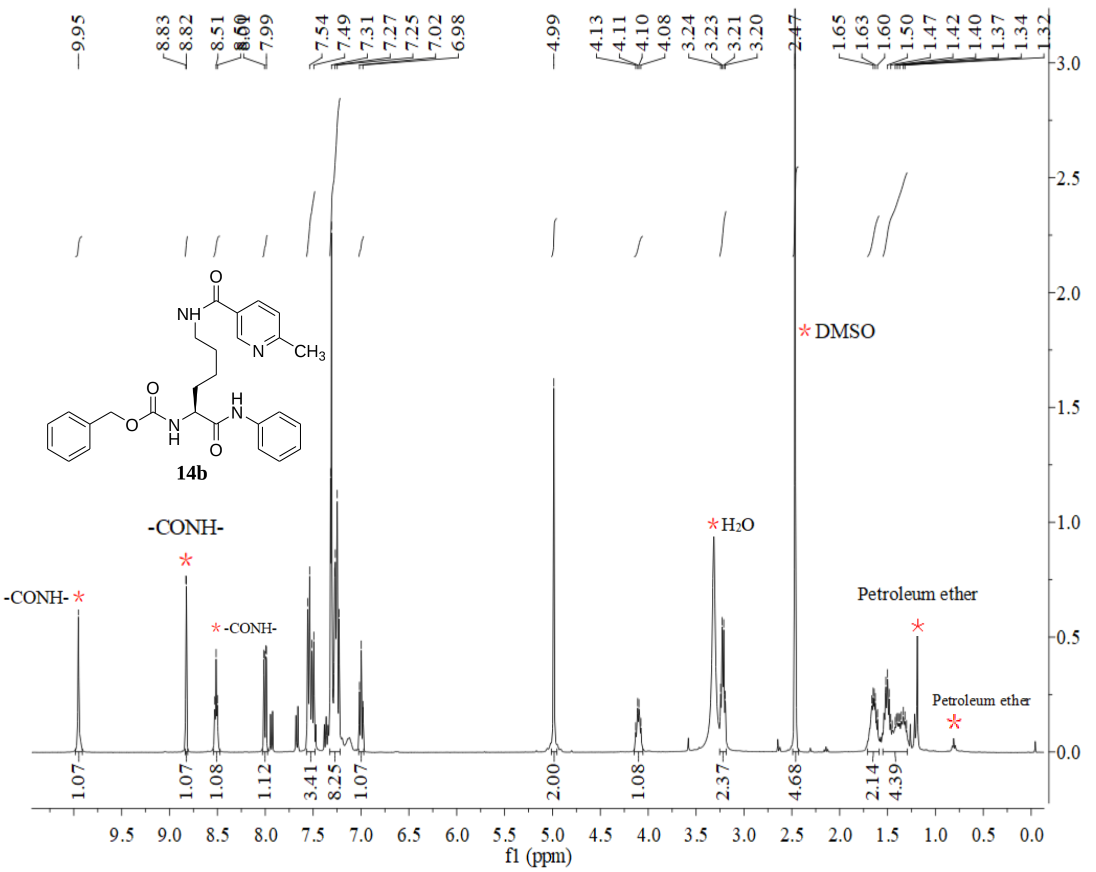

14b
-CONH-
-CONH-
-CONH-
H2O
DMSO
Petroleum ether
Petroleum ether
f1 (ppm)

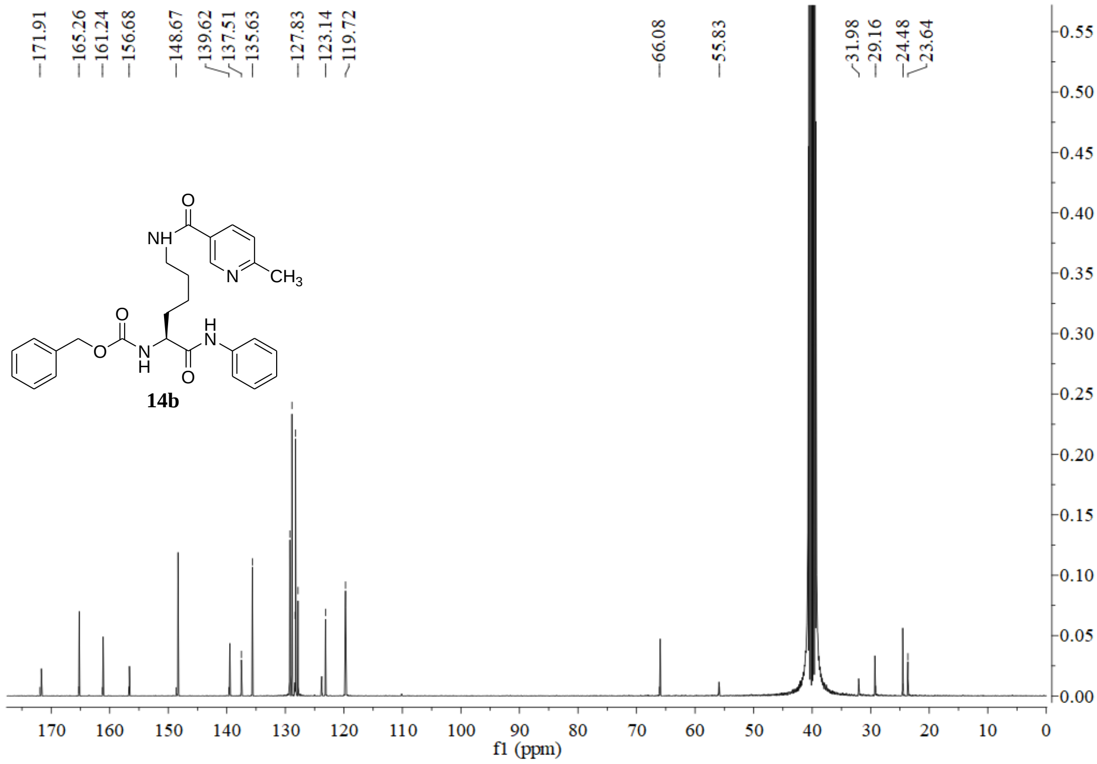

14b
f1 (ppm)

DMSO-d_6

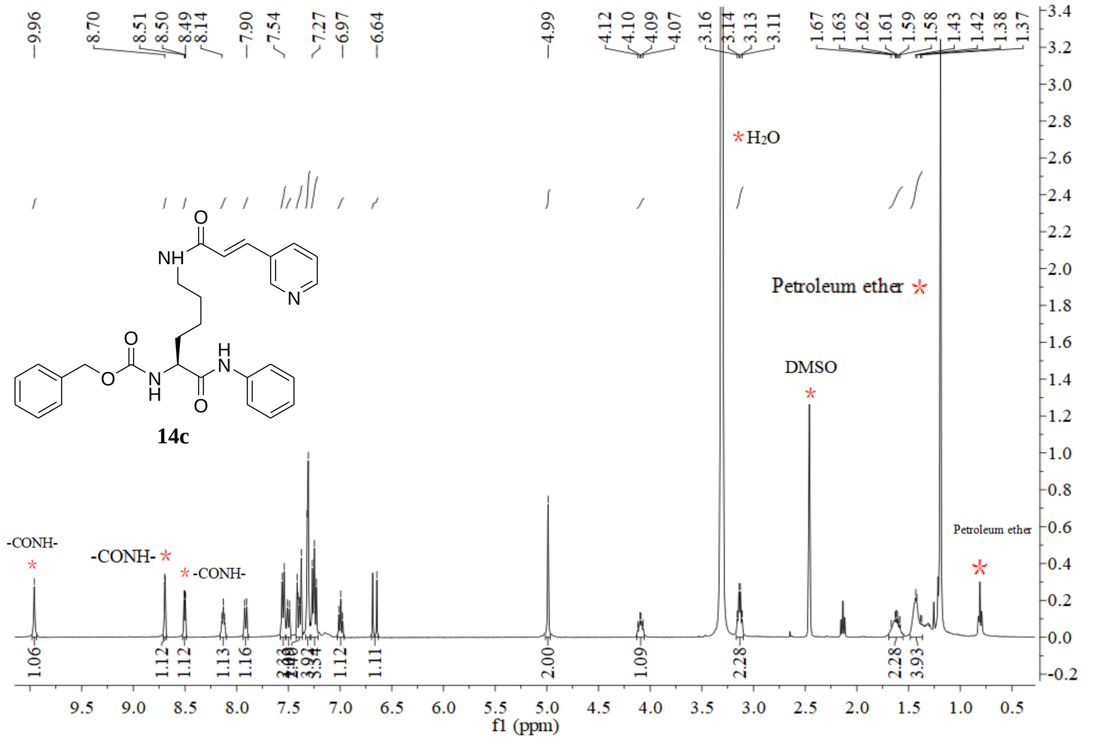

-CONH-
-CONH-
-CONH-
H2O
Petroleum ether
DMSO
Petroleum ether
14c
f1 (ppm)

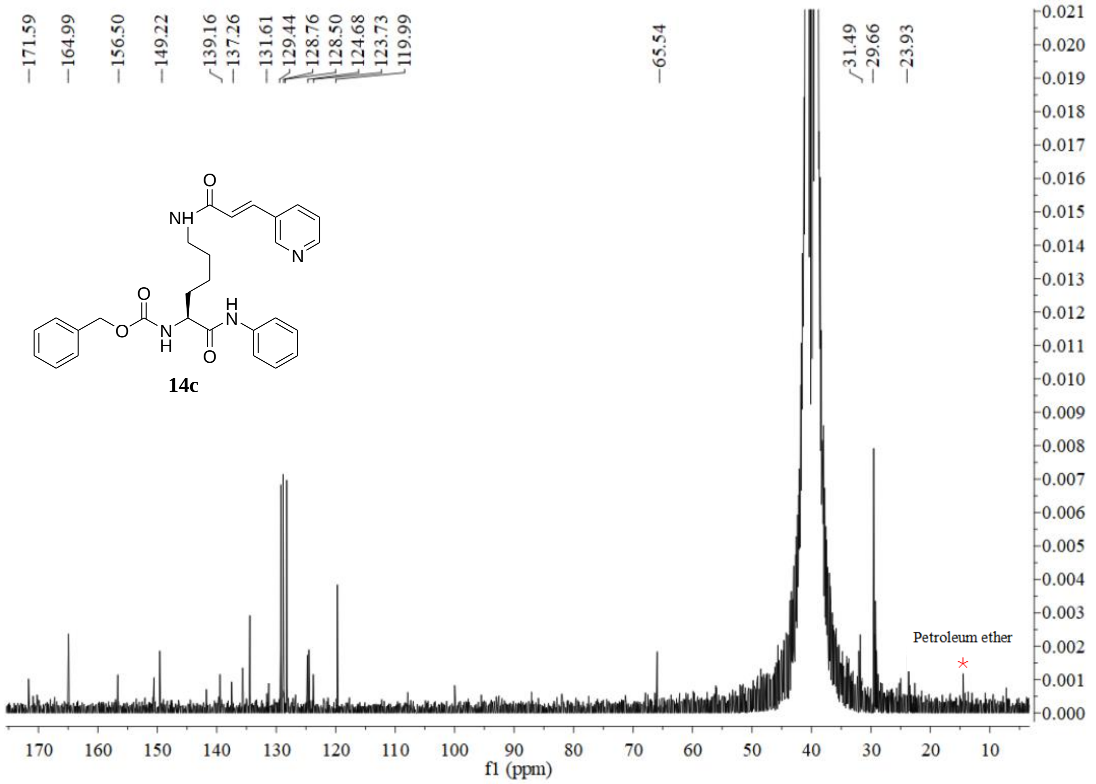

14c
Petroleum ether
f1 (ppm)

DMSO-d_6

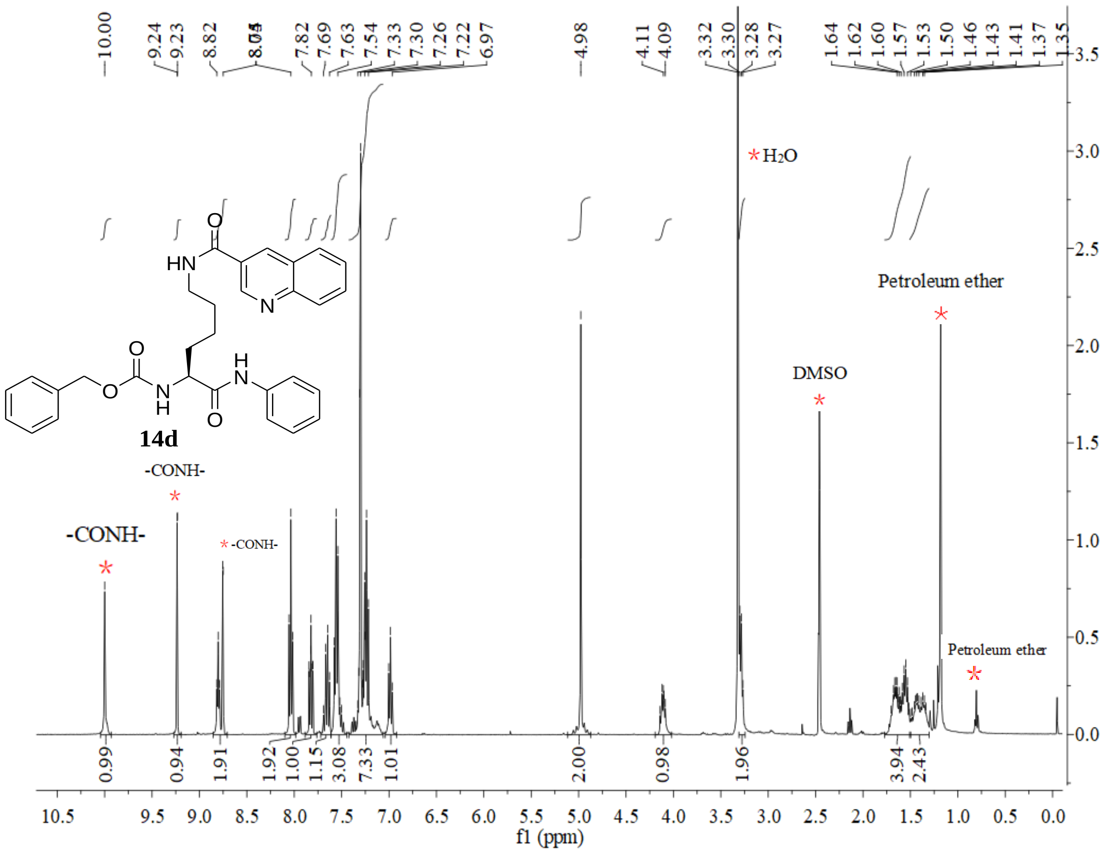
14d
-CONH-
*H2O
Petroleum ether
DMSO
f1 (ppm)

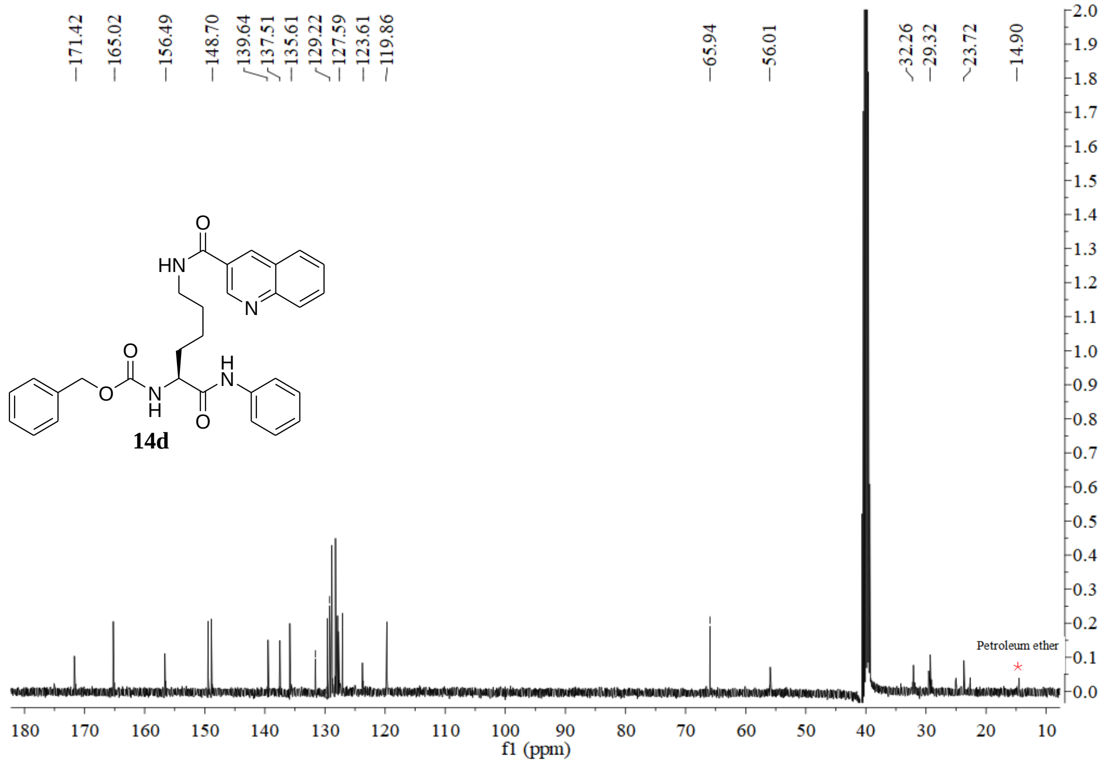
14d
Petroleum ether
f1 (ppm)

DMSO-d_6

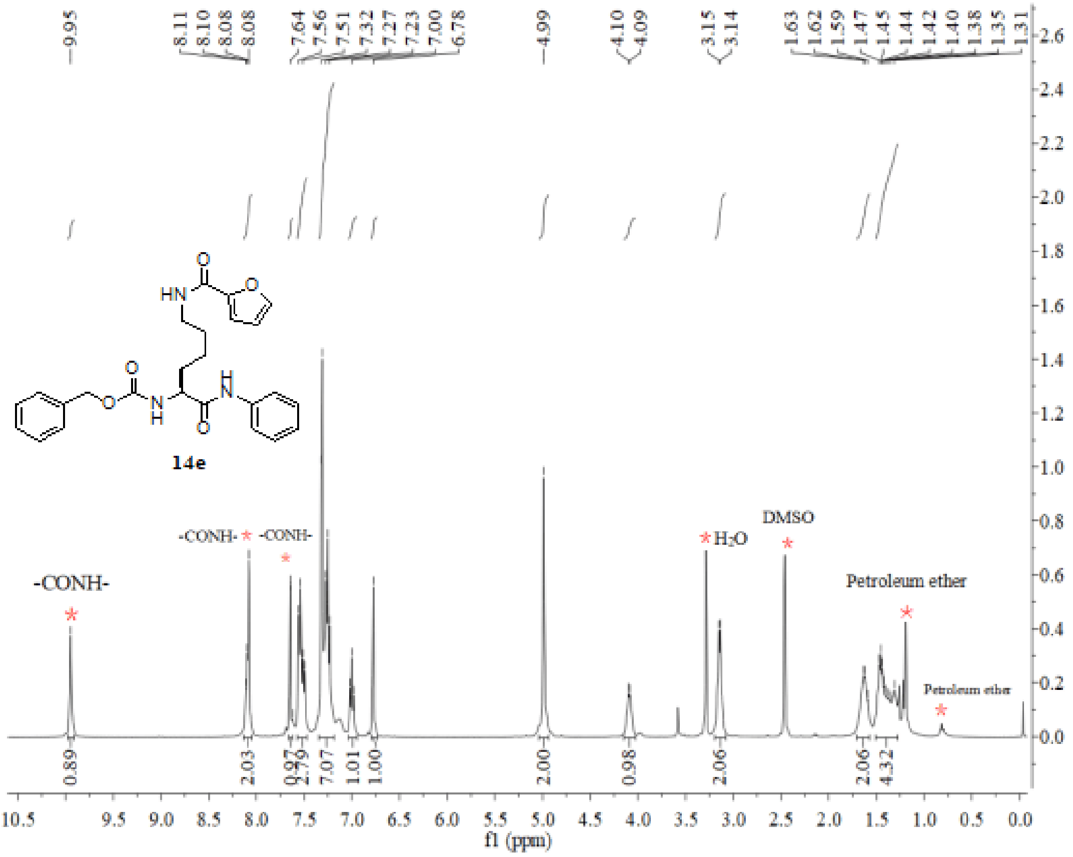

9.95
8.11
8.10
8.08
8.08
7.64
7.56
7.51
7.32
7.27
7.23
7.00
6.78
4.99
4.10
4.09
3.15
3.14
1.63
1.62
1.59
1.47
1.45
1.44
1.42
1.40
1.38
1.35
1.31
14e
-CONH-
-CONH-
-CONH-
H₂O
DMSO
Petroleum ether
Petroleum ether
0.89
2.03
0.97
2.76
7.07
1.01
1.00
2.00
0.98
2.06
2.06
4.32
f1 (ppm)

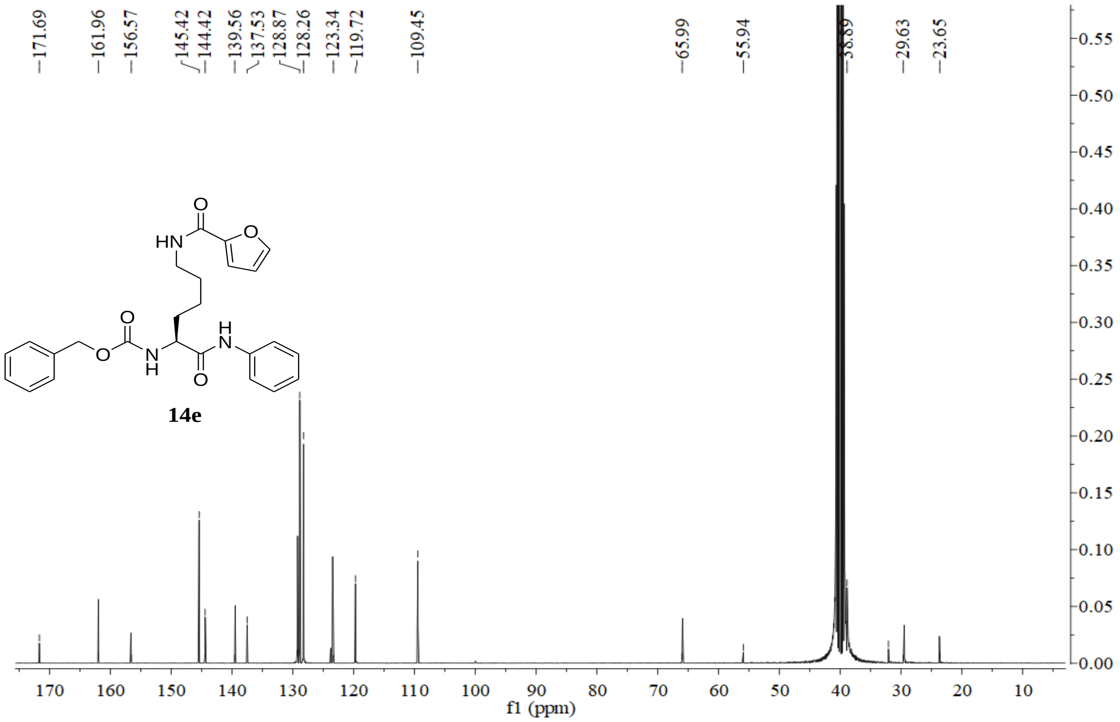

171.69
161.96
156.57
145.42
144.42
139.56
137.53
128.87
128.26
123.34
119.72
109.45
65.99
55.94
38.89
29.63
23.65
14e
f1 (ppm)

DMSO-d_6

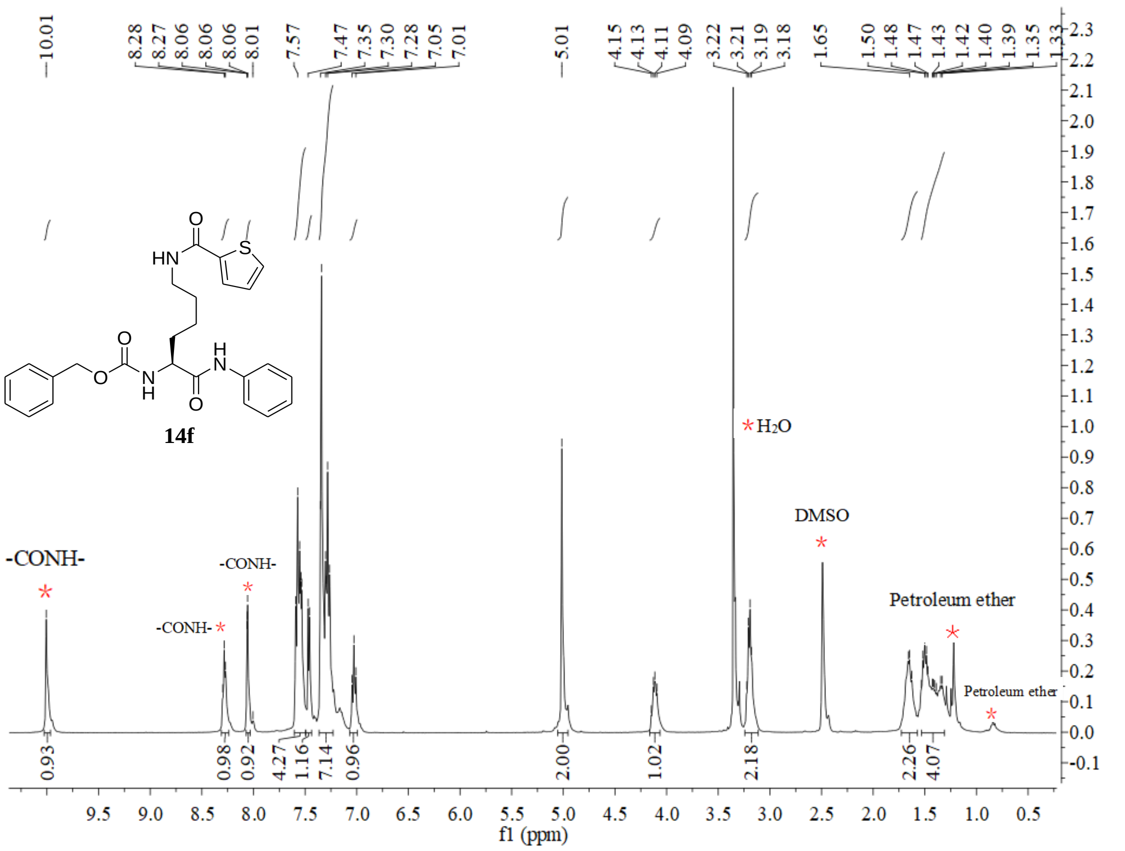

14f
-CONH-
-CONH-
-CONH-
H2O
DMSO
Petroleum ether
Petroleum ether
f1 (ppm)

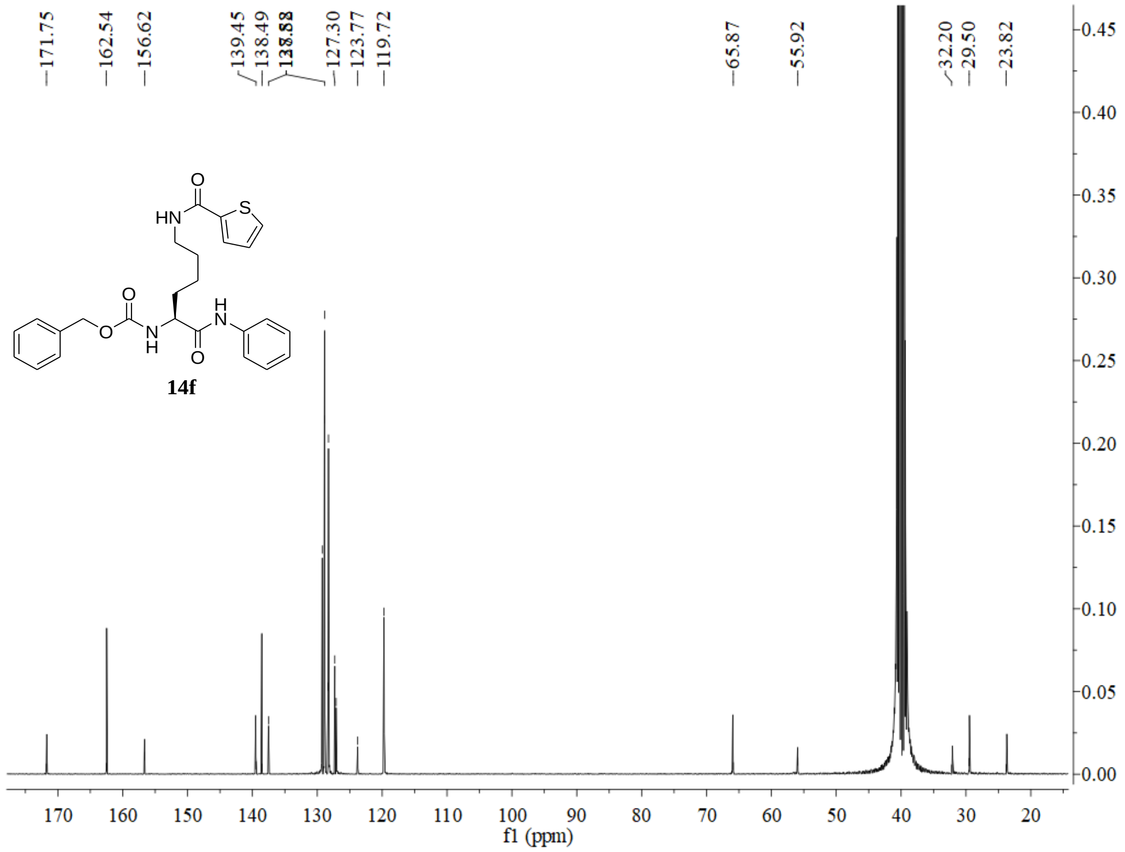

14f
f1 (ppm)

DMSO-d_6

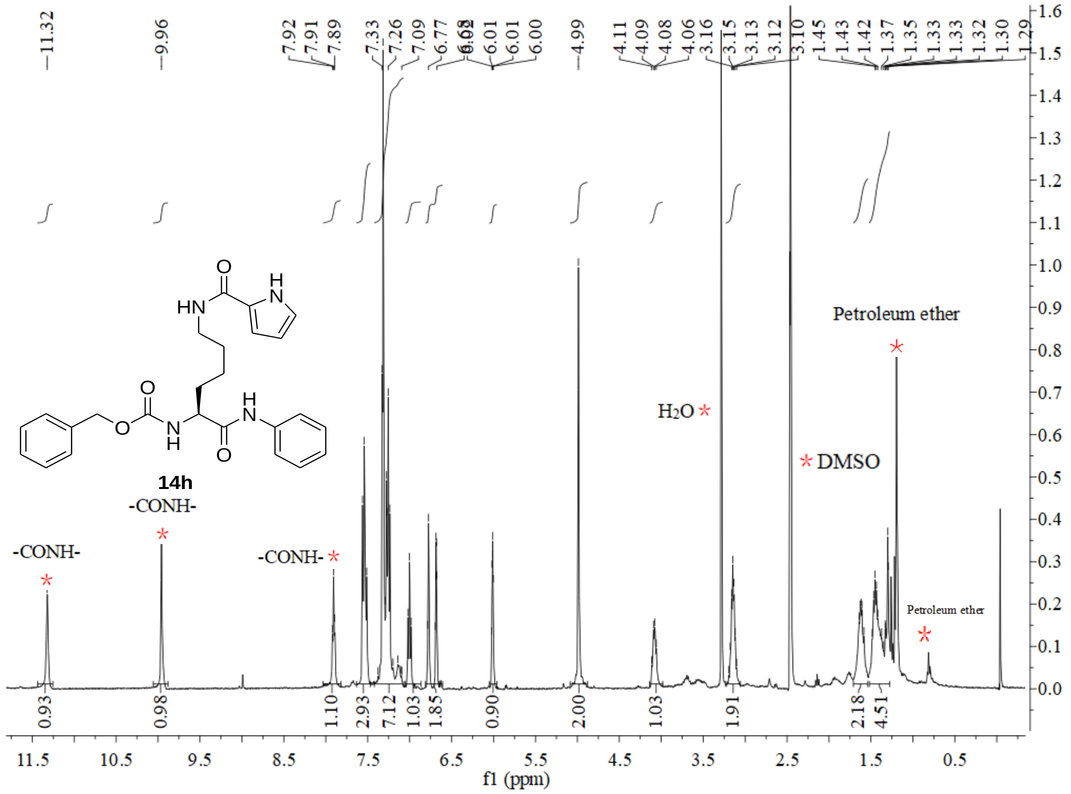

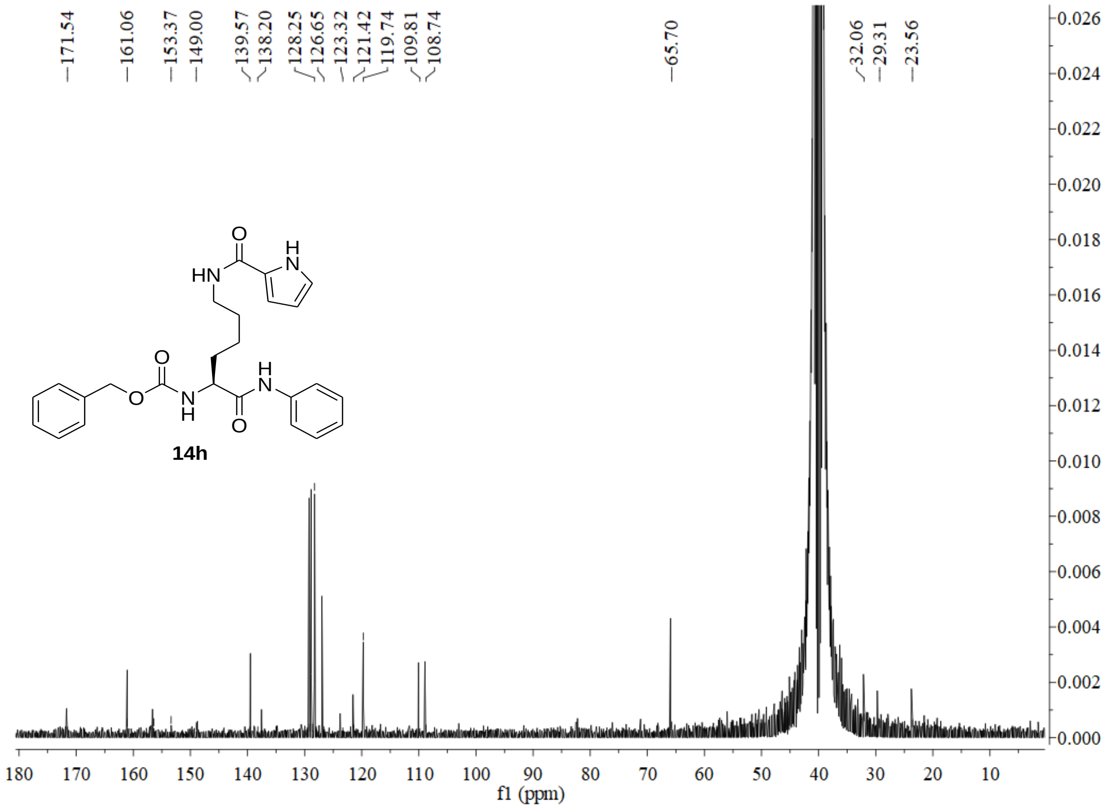

DMSO-d_6

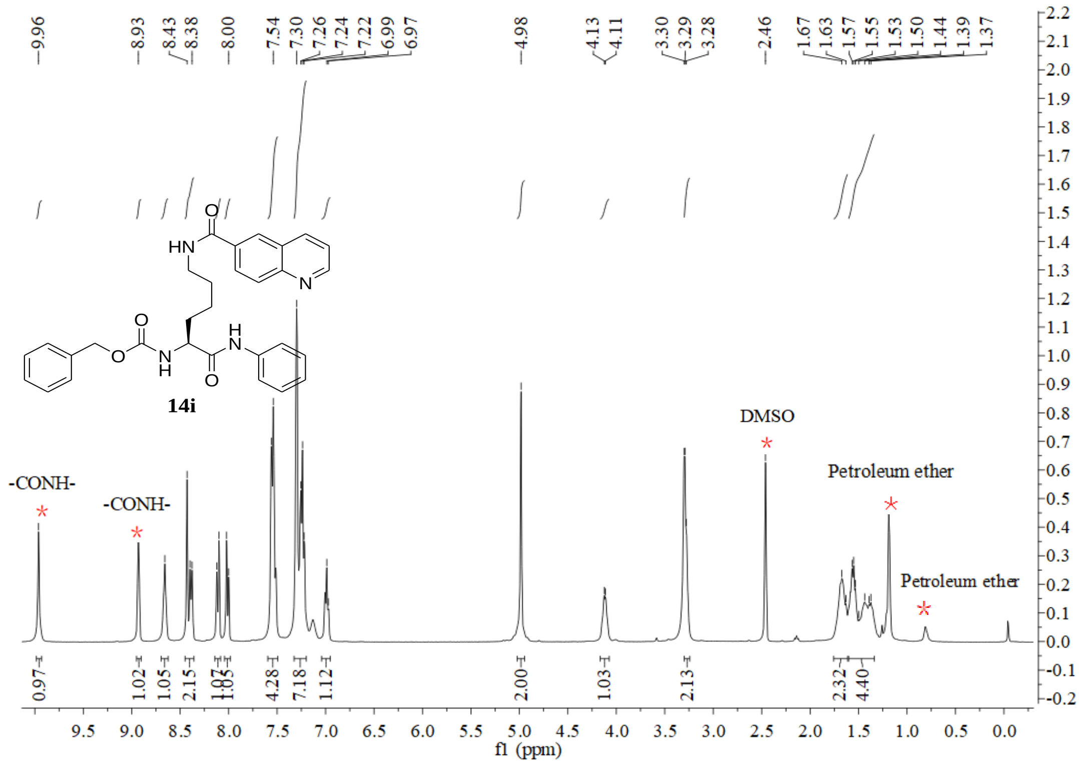

9.96
8.93
8.43
8.38
8.00
7.54
7.30
7.26
7.24
7.22
6.99
6.97
4.98
4.13
4.11
3.30
3.29
3.28
2.46
1.67
1.63
1.57
1.55
1.53
1.50
1.44
1.39
1.37
HN
O
N
O
O
N
H
H
N
O
14i
-CONH-
-CONH-
DMSO
Petroleum ether
Petroleum ether
0.97
1.02
1.05
2.15
1.07
1.05
4.28
7.18
1.12
2.00
1.03
2.13
2.32
4.40
f1 (ppm)

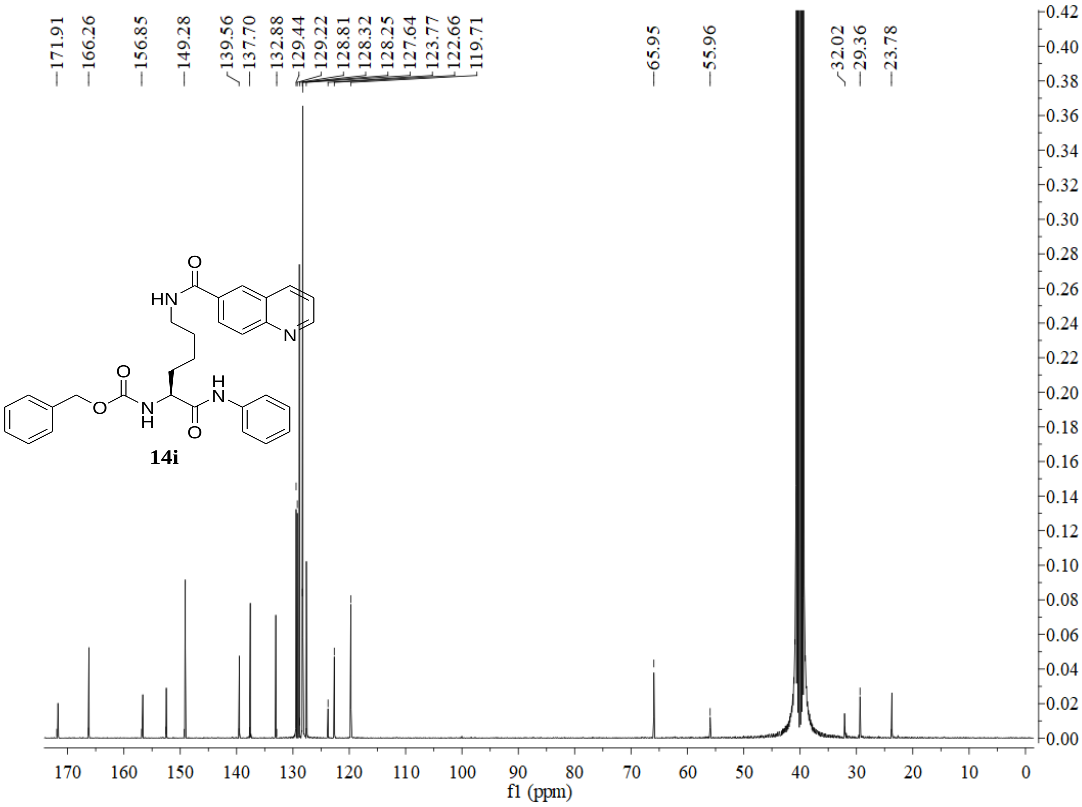

171.91
166.26
156.85
149.28
139.56
137.70
132.88
129.44
129.22
128.81
128.32
128.25
127.64
123.77
122.66
119.71
65.95
55.96
32.02
29.36
23.78
HN
O
N
O
O
N
H
H
N
O
14i
f1 (ppm)

DMSO-d_6

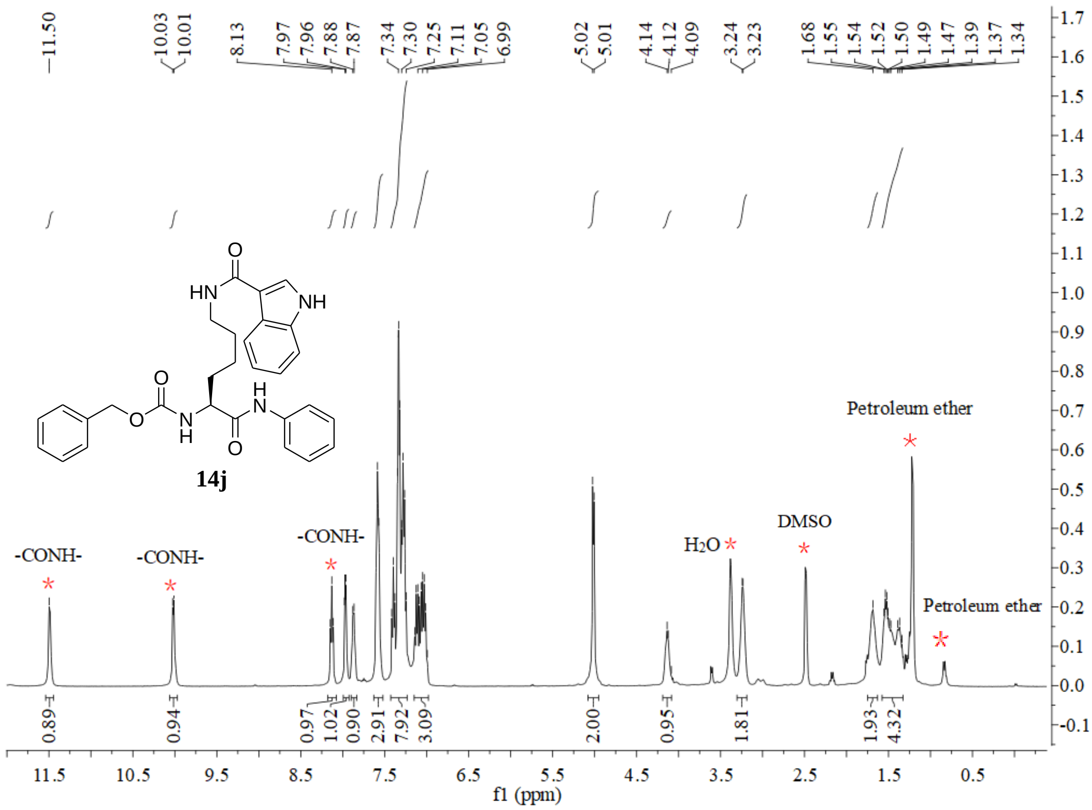

11.50
10.03
10.01
8.13
7.97
7.96
7.88
7.87
7.34
7.30
7.25
7.11
7.05
6.99
5.02
5.01
4.14
4.12
4.09
3.24
3.23
1.68
1.55
1.54
1.52
1.50
1.49
1.47
1.39
1.37
1.34
HN
NH
O
H
N
14j
-CONH-
-CONH-
-CONH-
H2O
DMSO
Petroleum ether
Petroleum ether
0.89
0.94
0.97
1.02
0.90
2.91
7.92
3.09
2.00
0.95
1.81
1.93
4.32
f1 (ppm)

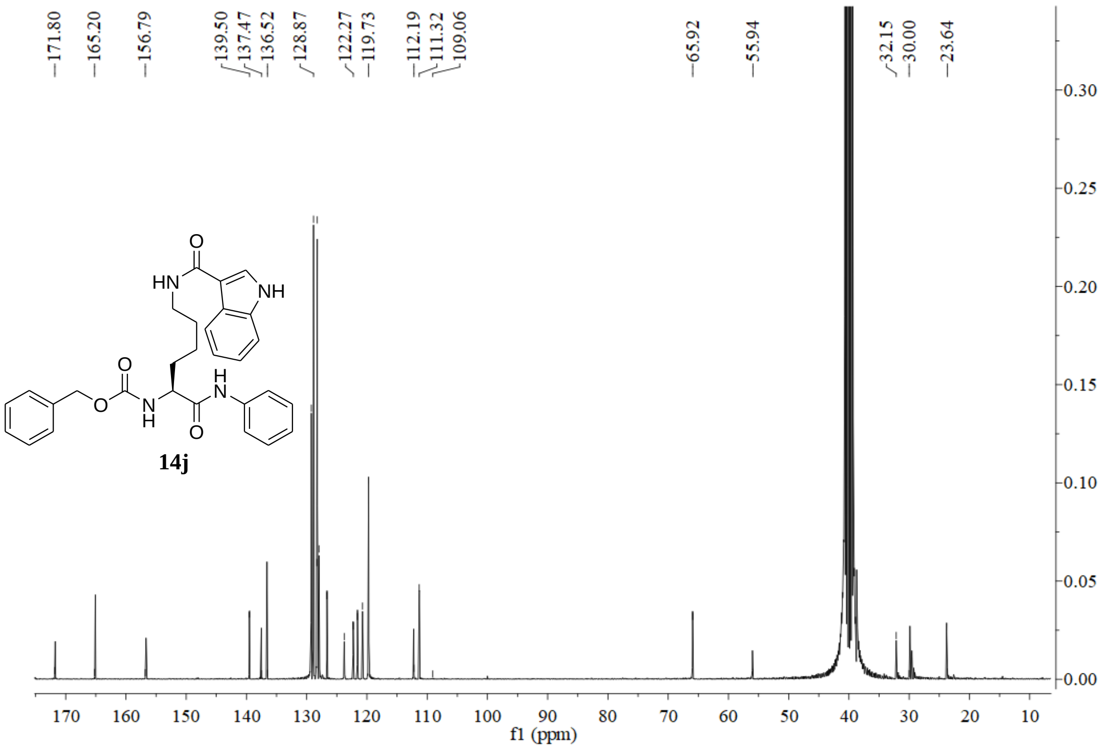

171.80
165.20
156.79
139.50
137.47
136.52
128.87
122.27
119.73
112.19
111.32
109.06
65.92
55.94
32.15
30.00
23.64
HN
NH
O
H
N
14j
f1 (ppm)

DMSO-d_6

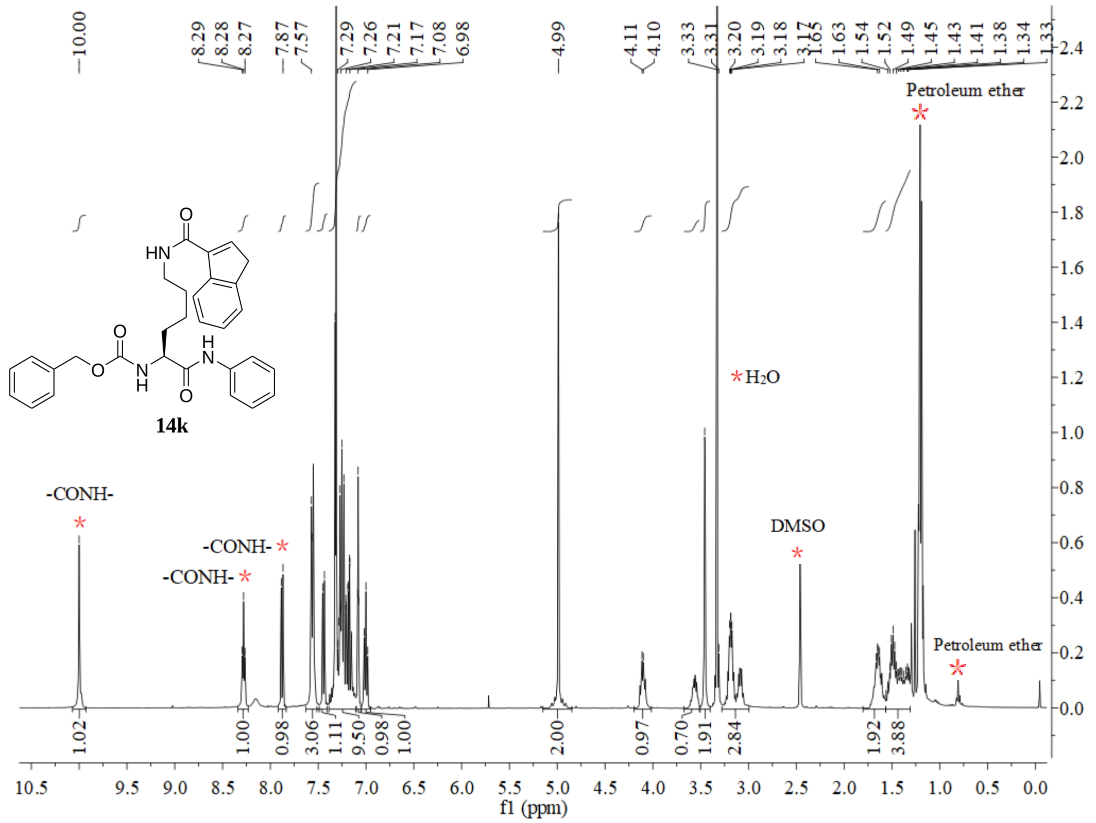

14k
-CONH-
-CONH-
-CONH-
*H2O
DMSO
Petroleum ether
Petroleum ether
f1 (ppm)

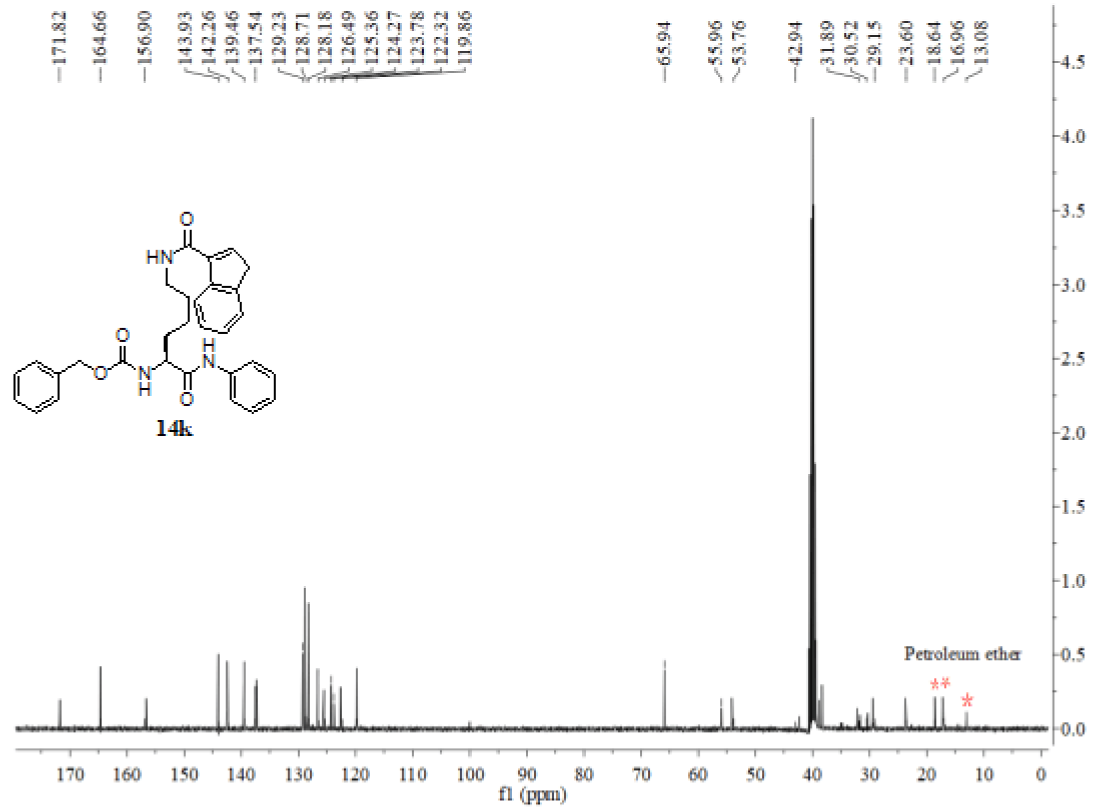

14k
Petroleum ether
f1 (ppm)

DMSO-d_6

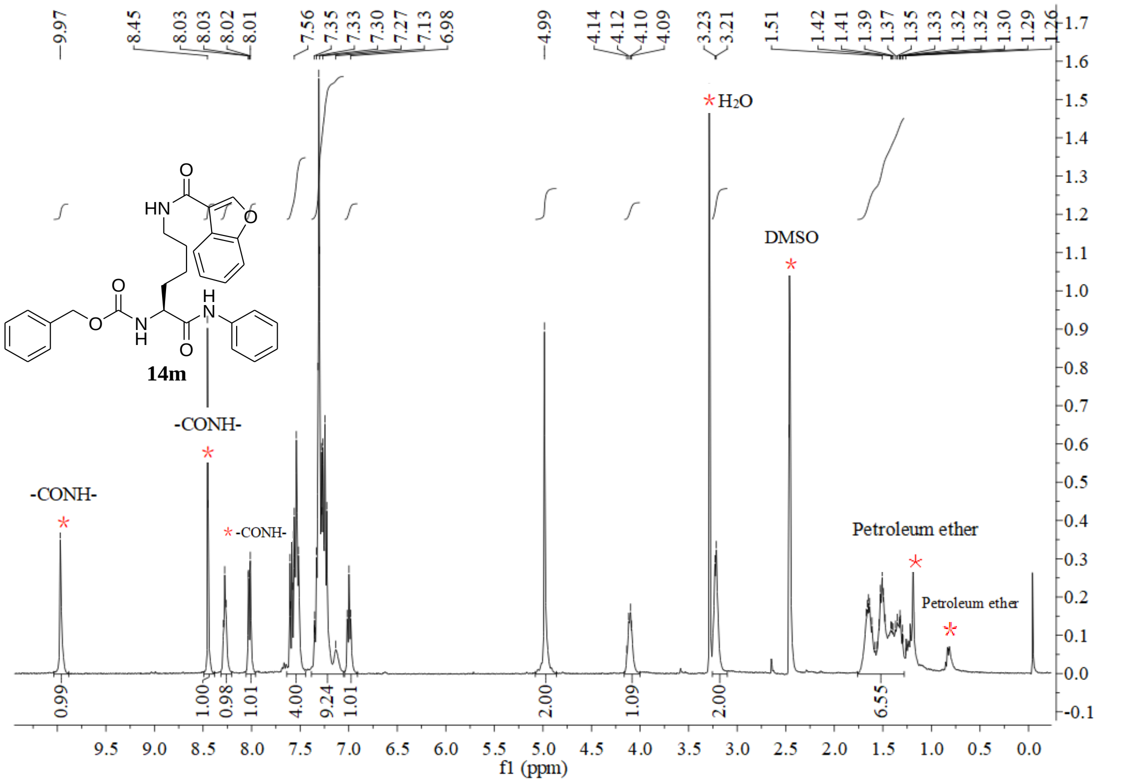

14m
-CONH-
-CONH-
-CONH-
H2O
DMSO
Petroleum ether
Petroleum ether
f1 (ppm)

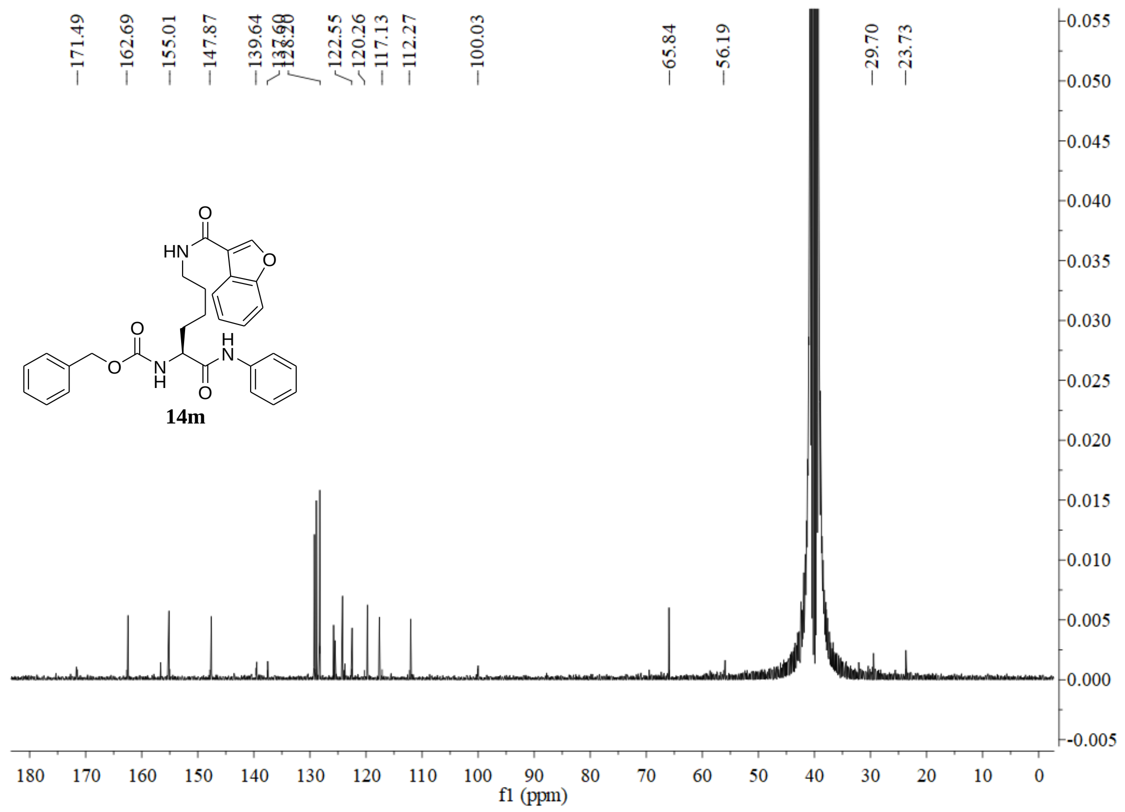

14m
f1 (ppm)

DMSO-d_6

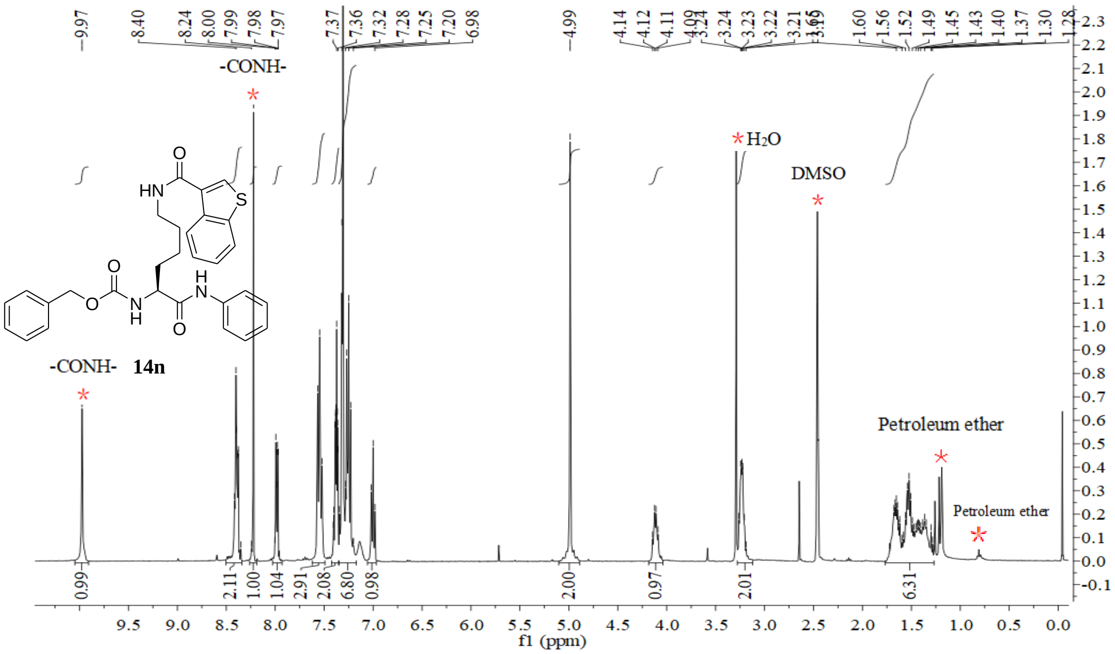

-CONH-
*H2O
DMSO
-CONH-
14n
Petroleum ether
Petroleum ether
f1 (ppm)

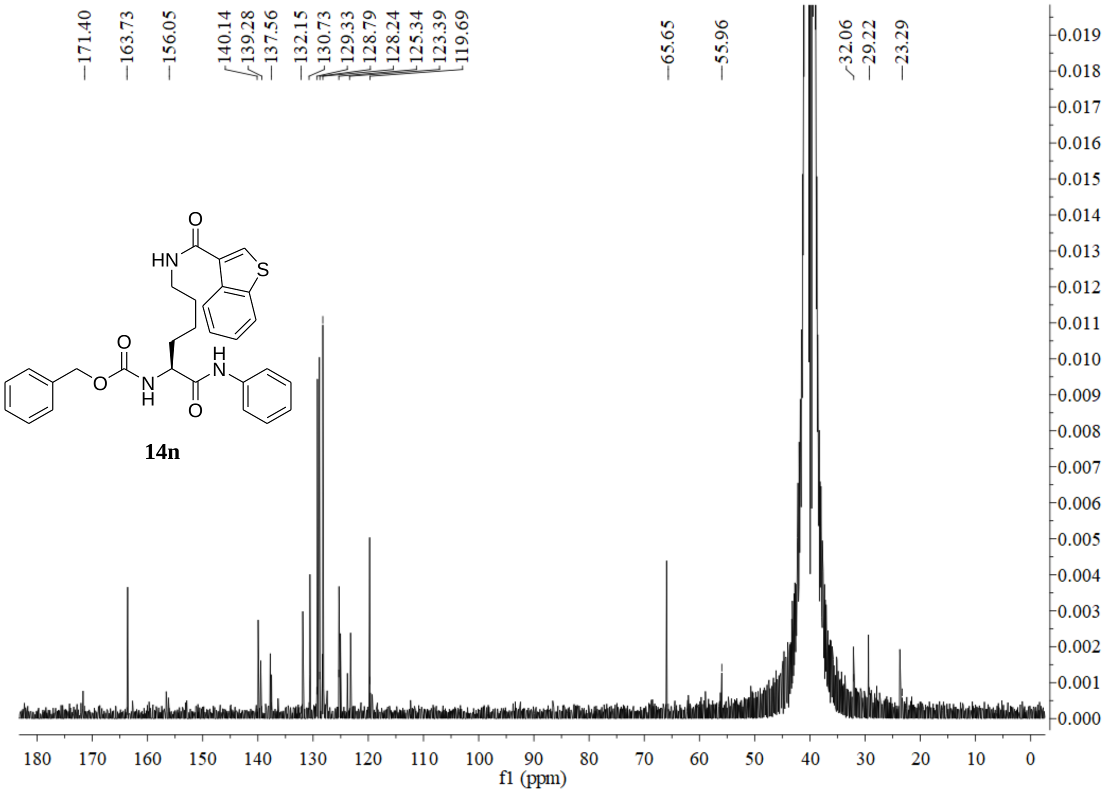

14n
f1 (ppm)

DMSO-d_6

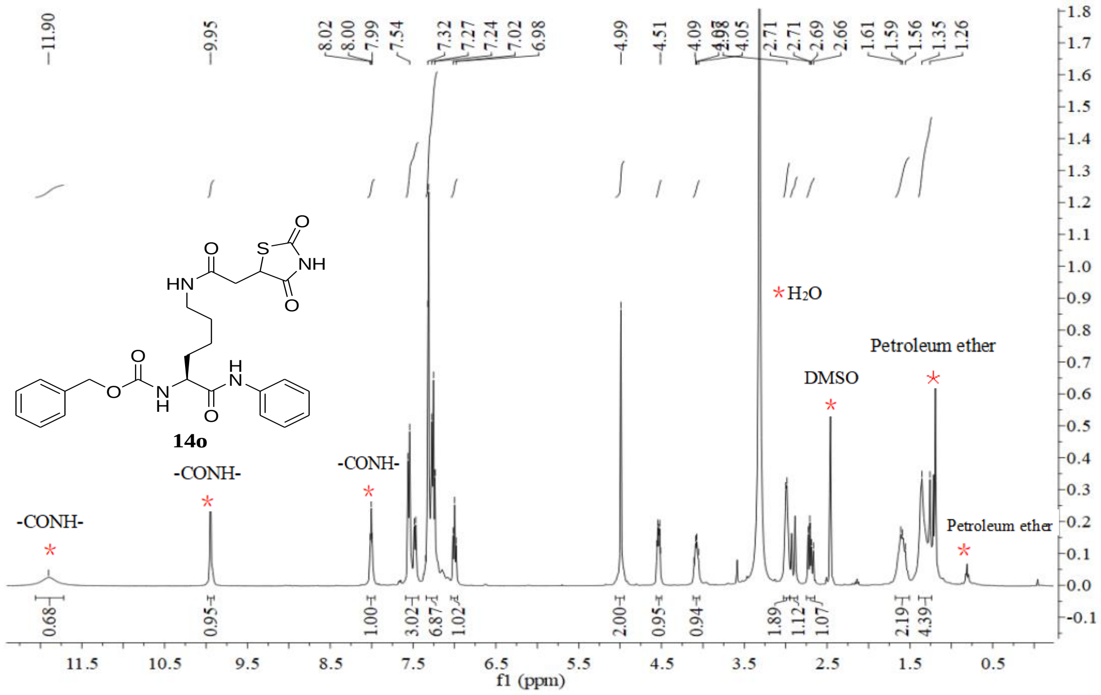
11.90
9.95
8.02
8.00
7.99
7.54
7.32
7.27
7.24
7.02
6.98
4.99
4.51
4.09
4.07
4.05
2.71
2.71
2.69
2.66
1.61
1.59
1.56
1.35
1.26
14o
-CONH-
-CONH-
-CONH-
* H2O
DMSO
Petroleum ether
Petroleum ether
0.68
0.95
1.00
3.02
6.87
1.02
2.00
0.95
0.94
1.89
1.12
1.07
2.19
4.39
f1 (ppm)

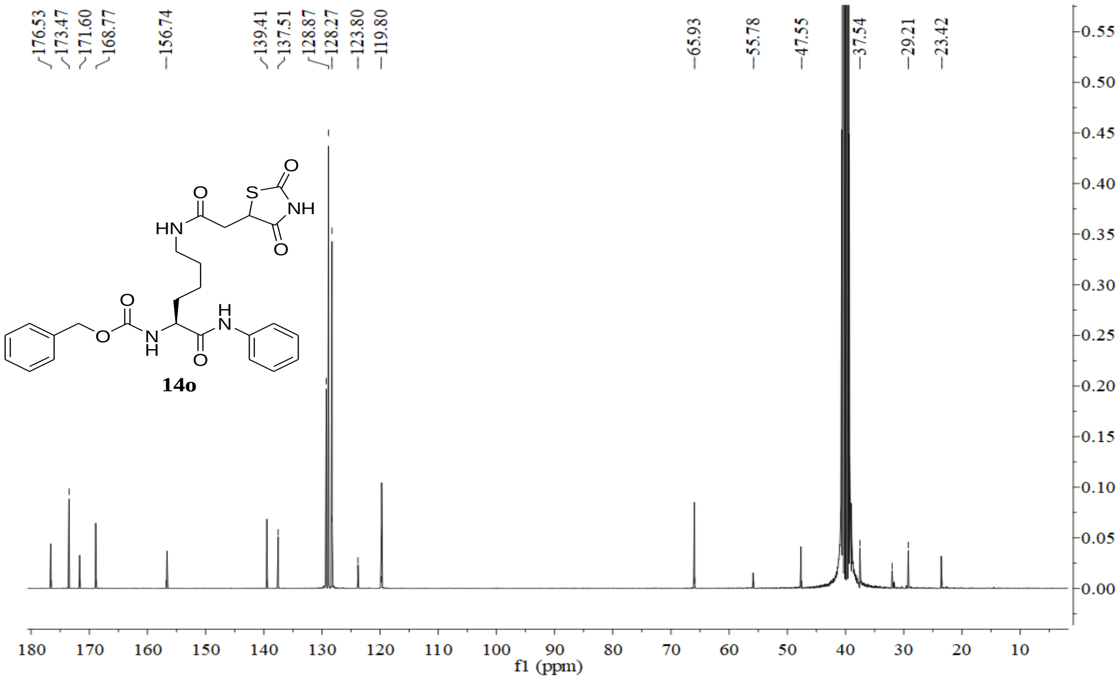
176.53
173.47
171.60
168.77
156.74
139.41
137.51
128.87
128.27
123.80
119.80
65.93
55.78
47.55
37.54
29.21
23.42
14o
f1 (ppm)

DMSO-d_6

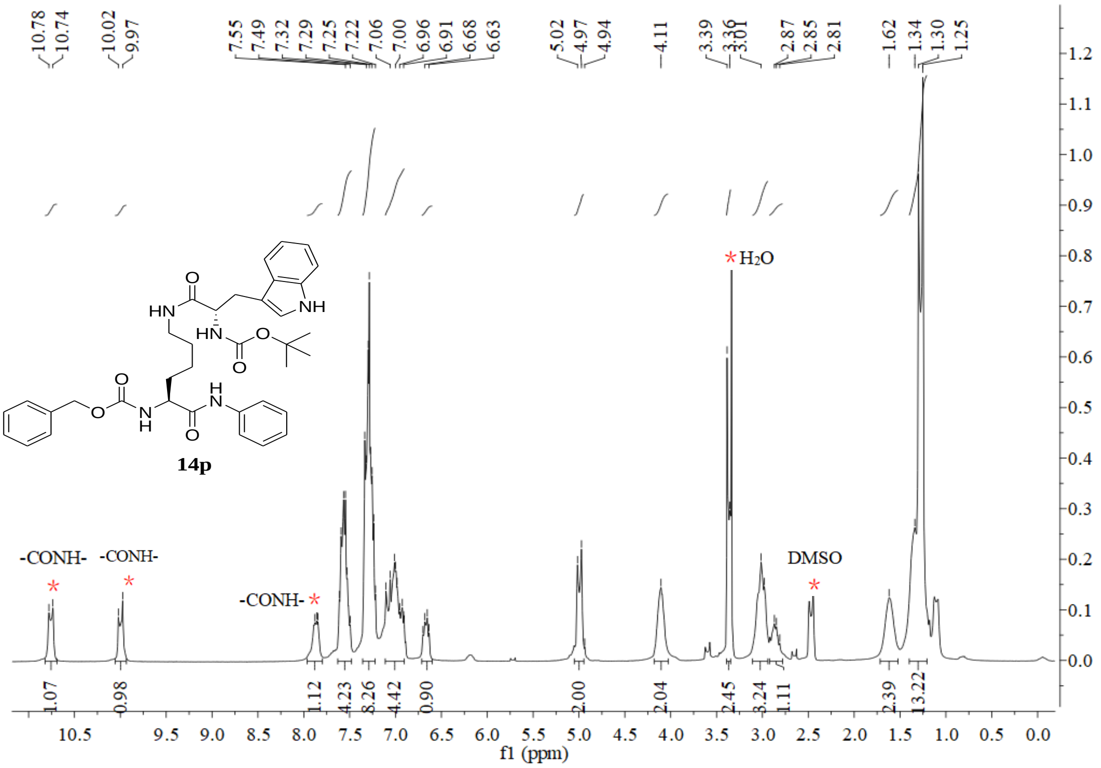

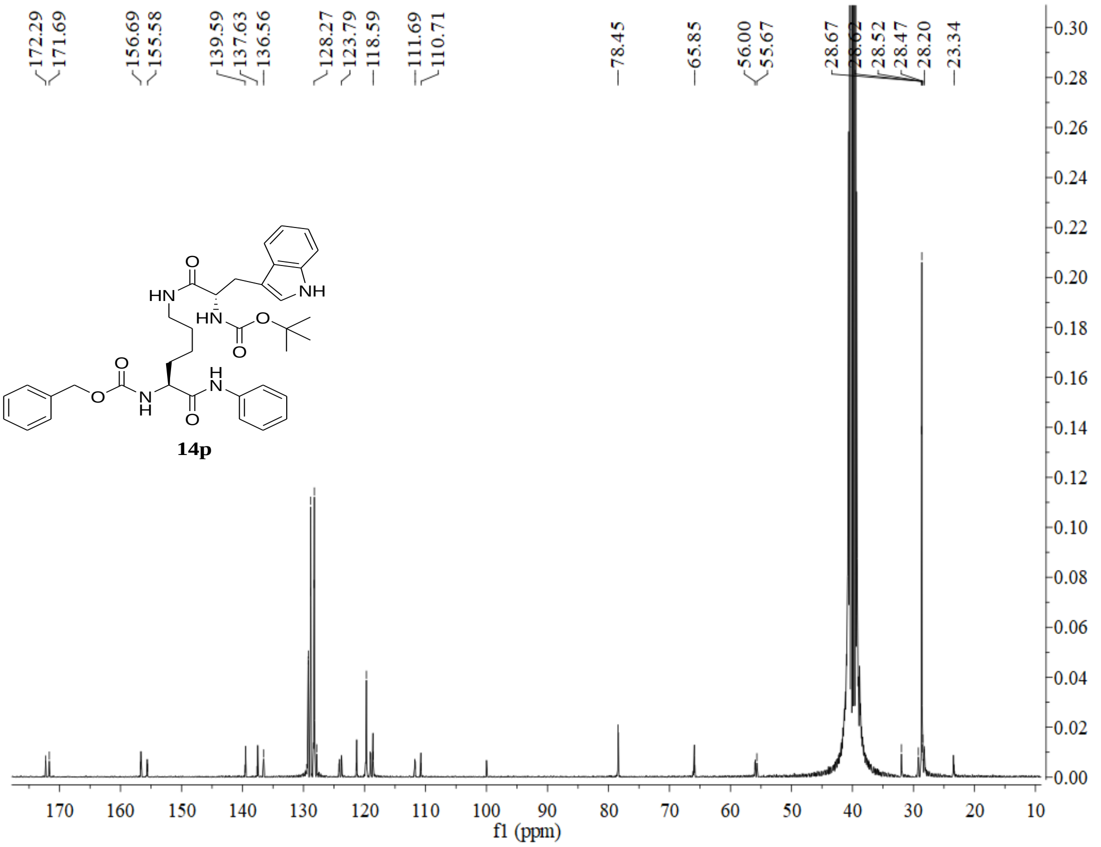

DMSO-d_6

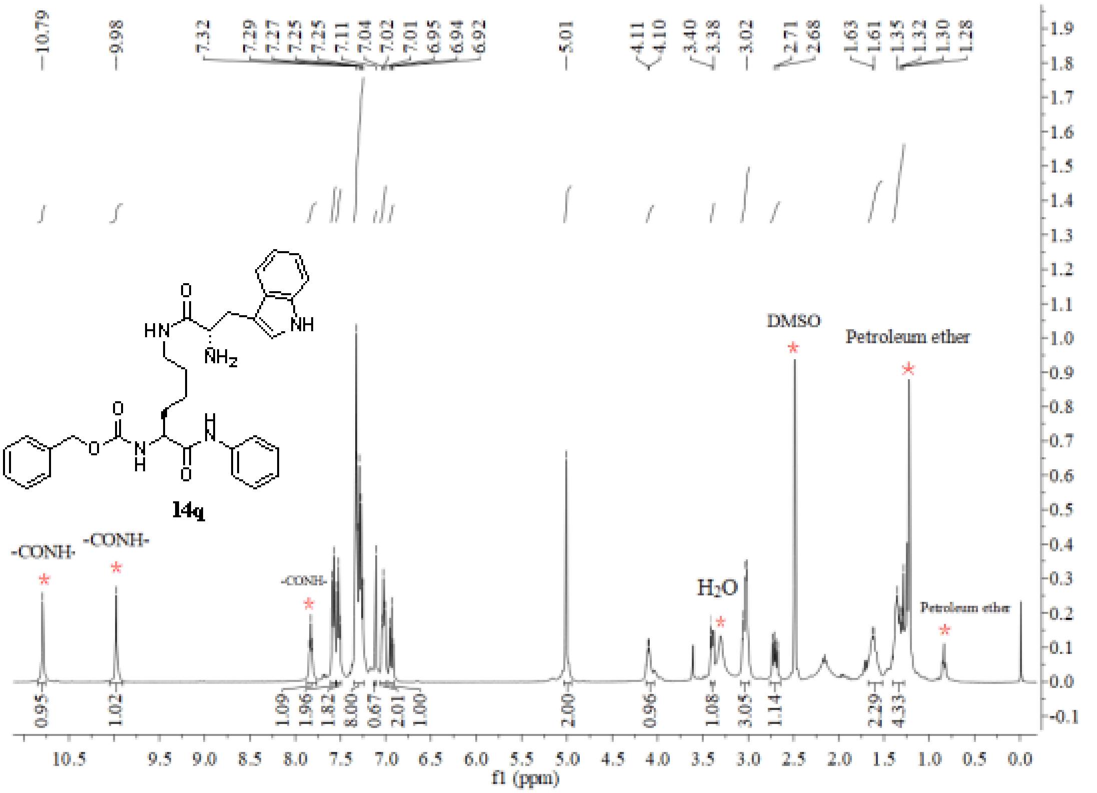

10.79
9.98
7.32
7.29
7.27
7.25
7.25
7.11
7.04
7.02
7.01
6.95
6.94
6.92
5.01
4.11
4.10
3.40
3.38
3.02
2.71
2.68
1.63
1.61
1.35
1.32
1.30
1.28
14q
-CONH-
-CONH-
-CONH-
DMSO
Petroleum ether
H2O
Petroleum ether
0.95
1.02
1.09
1.96
1.82
8.00
0.67
2.01
1.00
2.00
0.96
1.08
3.05
1.14
2.29
4.33
f1 (ppm)

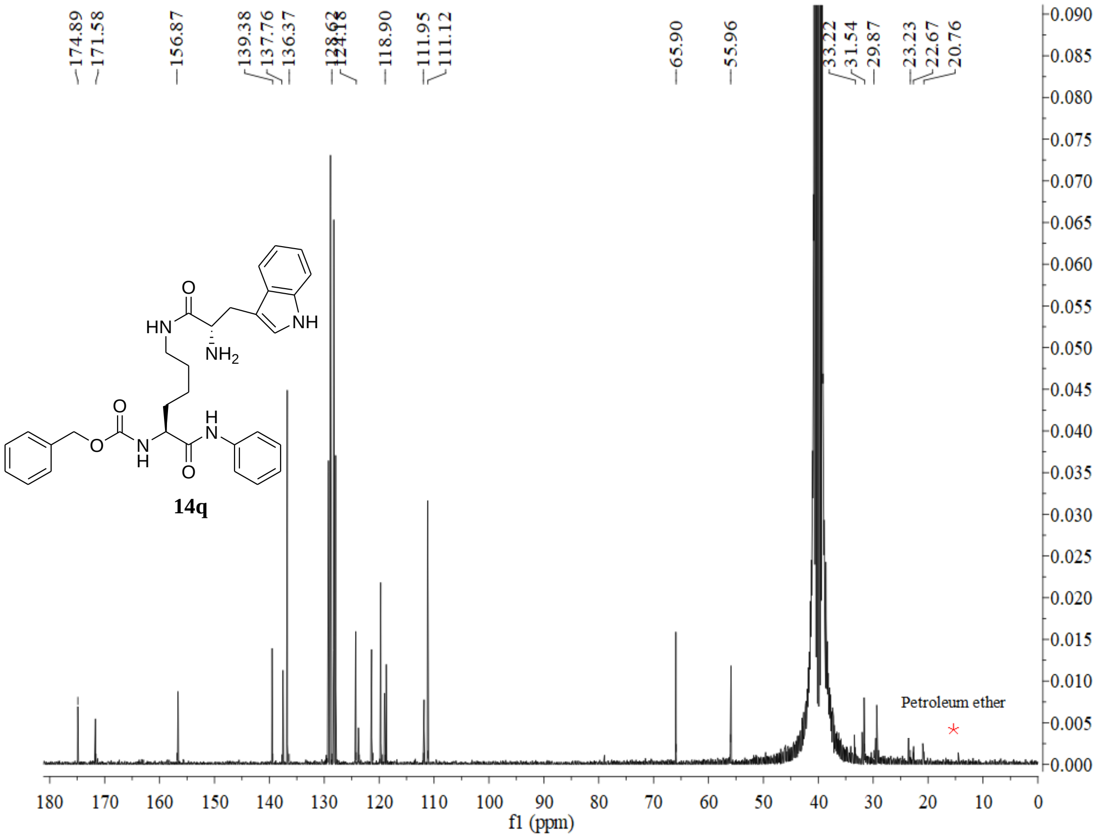

174.89
171.58
156.87
139.38
137.76
136.37
128.62
124.18
118.90
111.95
111.12
65.90
55.96
33.22
31.54
29.87
23.23
22.67
20.76
14q
Petroleum ether
f1 (ppm)

CD$_3$OD

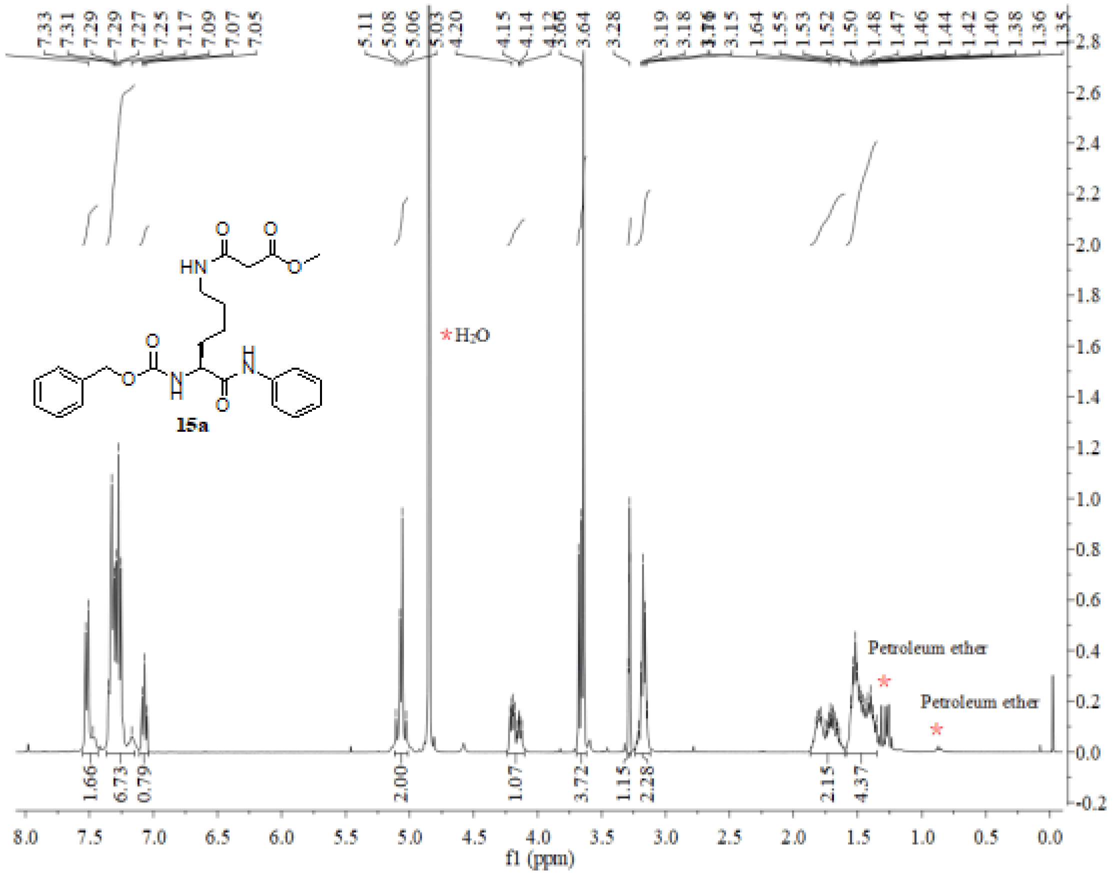

7.33 7.31 7.29 7.29 7.27 7.25 7.17 7.09 7.07 7.05 5.11 5.08 5.06 5.03 4.20 4.15 4.14 4.12 3.65 3.64 3.28 3.19 3.18 3.16 3.15 1.64 1.55 1.53 1.52 1.50 1.48 1.47 1.46 1.44 1.42 1.40 1.38 1.36 1.35
HN
O
O
O
O
N
H
H
N
O
15a
*H2O
Petroleum ether
Petroleum ether
1.66 6.73 0.79 2.00 1.07 3.72 1.15 2.28 2.15 4.37
f1 (ppm)

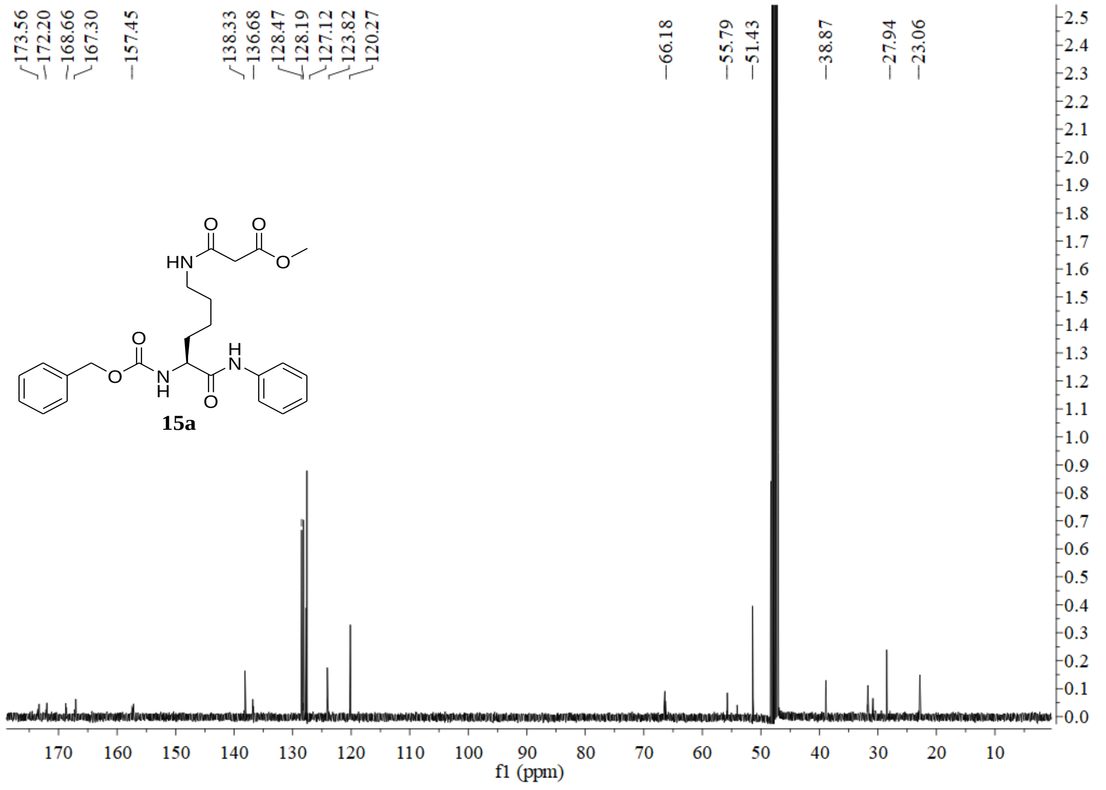

173.56 172.20 168.66 167.30 157.45 138.33 136.68 128.47 128.19 127.12 123.82 120.27 66.18 55.79 51.43 38.87 27.94 23.06
HN
O
O
O
O
N
H
H
N
O
15a
f1 (ppm)

DMSO-d_6

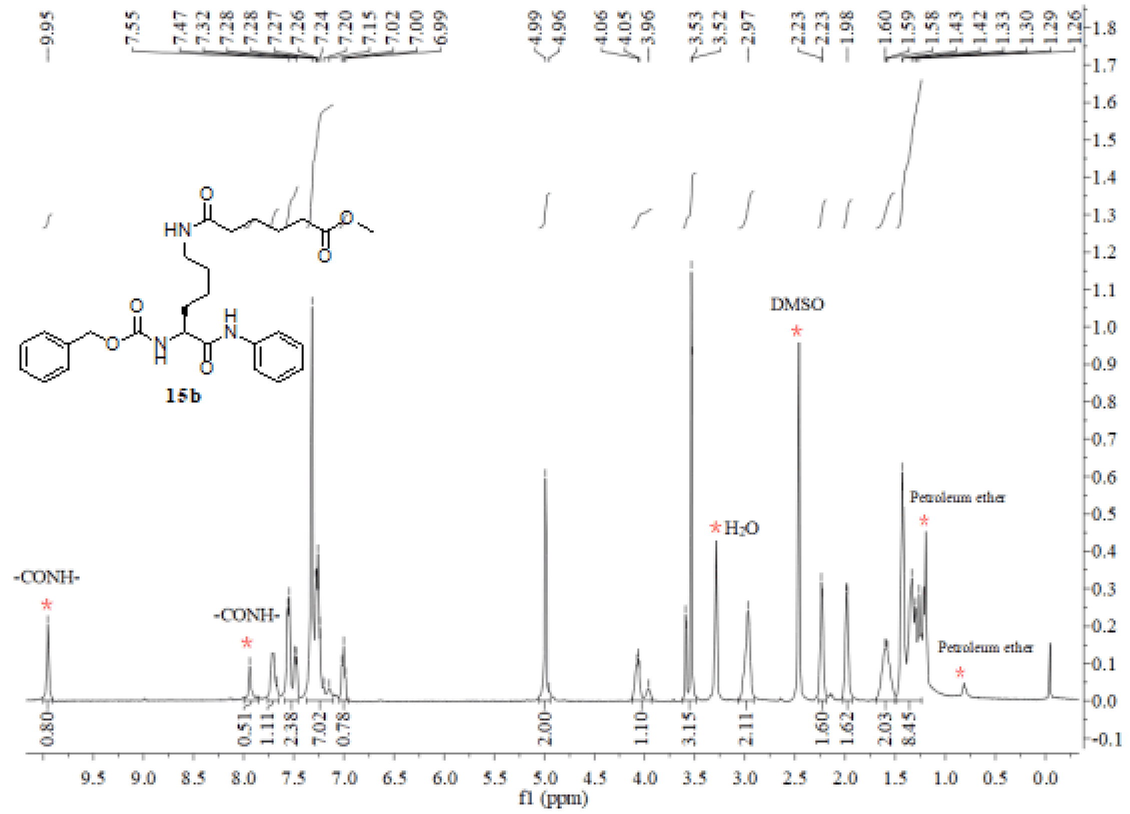

-CONH-
-CONH-
DMSO
H₂O
Petroleum ether
Petroleum ether
15b
f1 (ppm)

CD$_3$OD

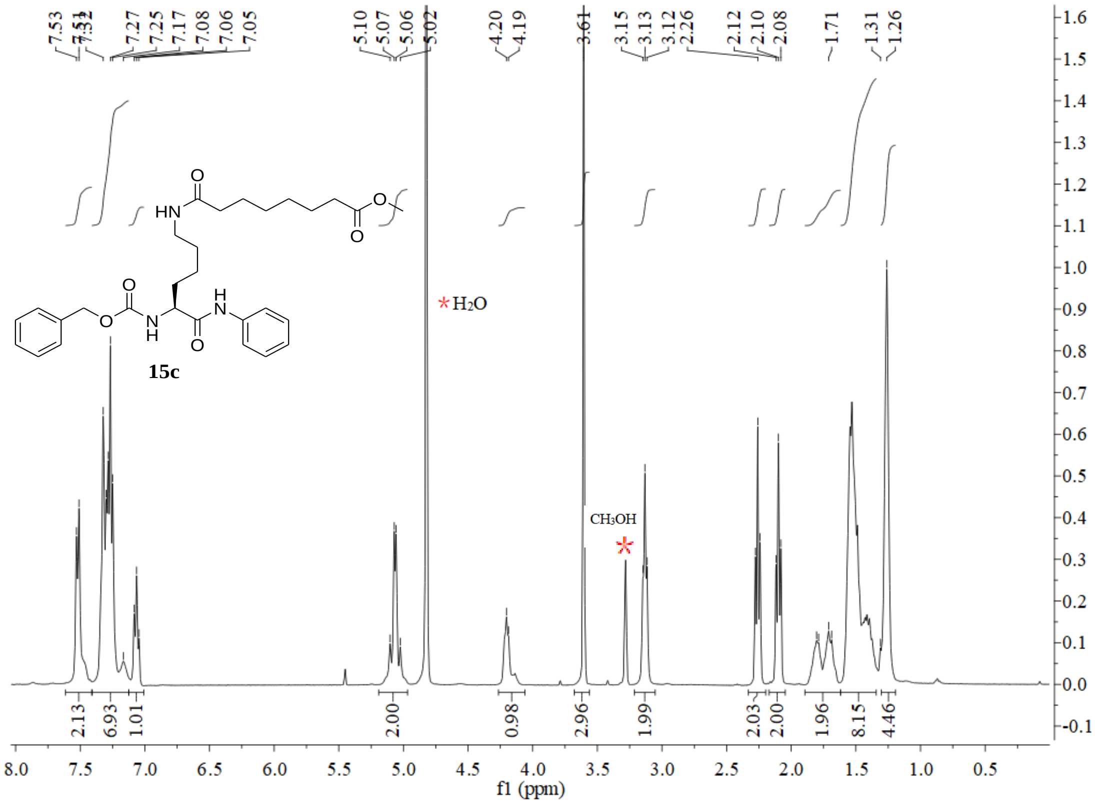

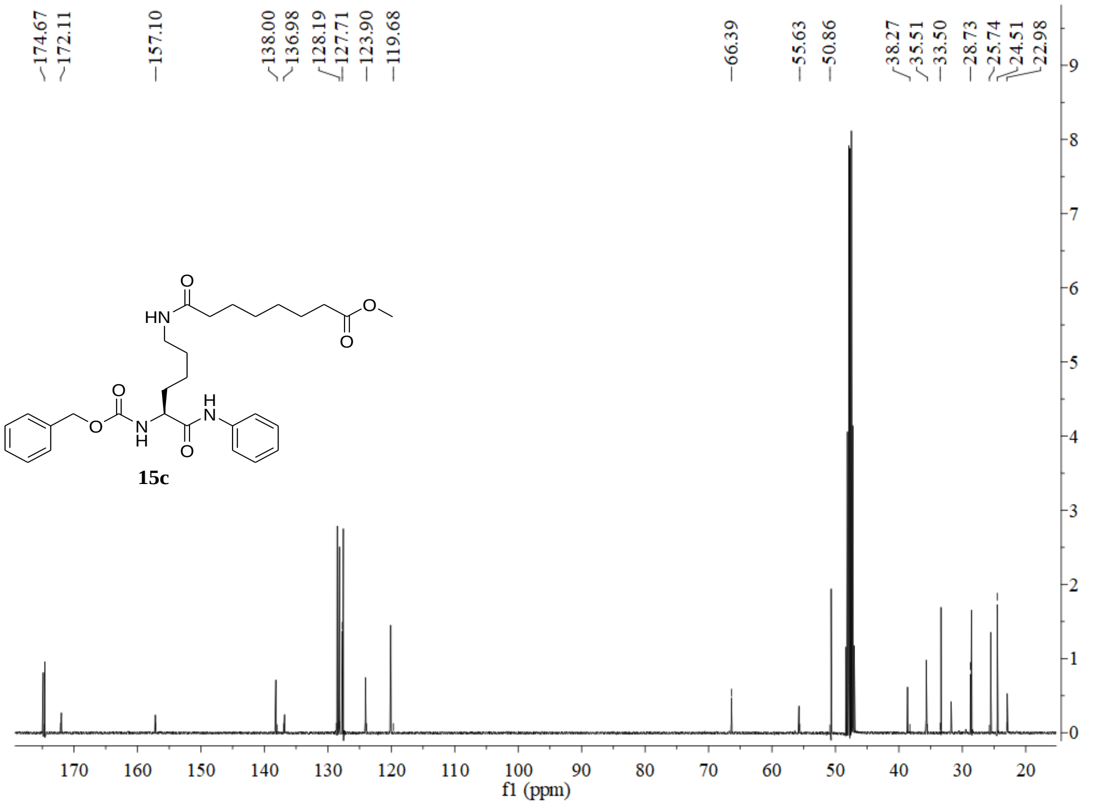

CD$_3$OD

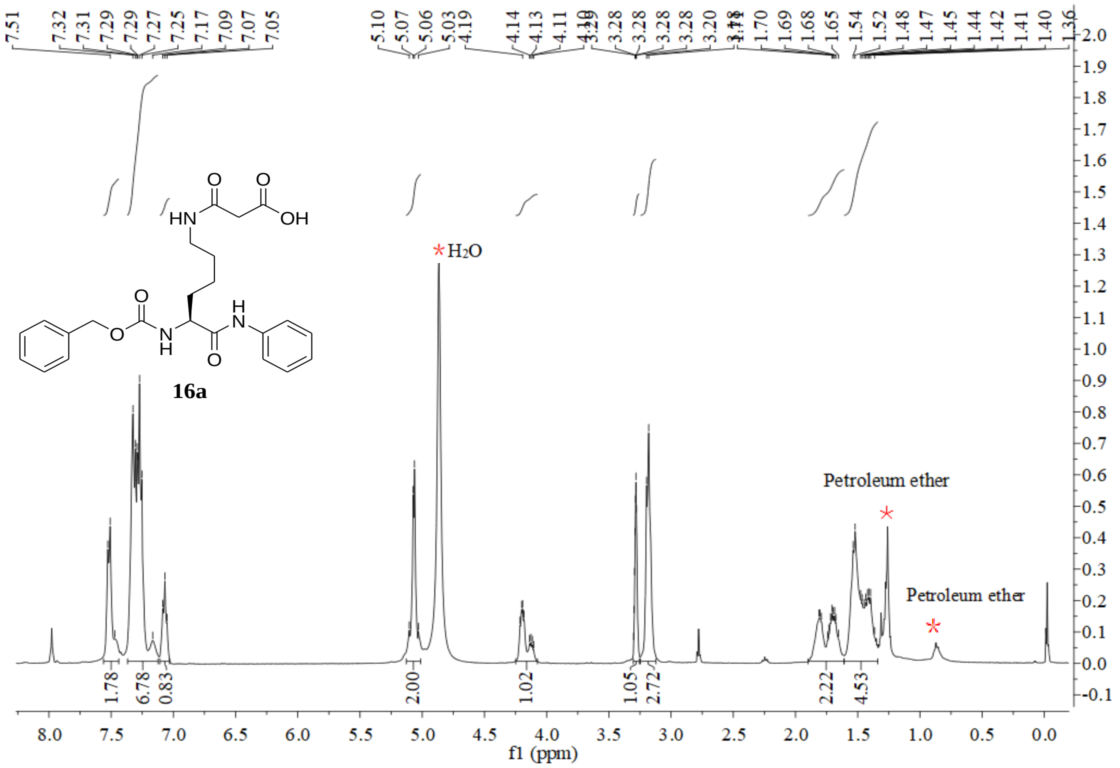
7.51
7.32
7.31
7.29
7.29
7.27
7.25
7.17
7.09
7.07
7.05
5.10
5.07
5.06
5.03
4.19
4.14
4.13
4.11
3.28
3.28
3.28
3.28
3.20
1.70
1.69
1.68
1.65
1.54
1.52
1.48
1.47
1.45
1.44
1.42
1.41
1.40
1.36
*H2O
16a
Petroleum ether
Petroleum ether
1.78
6.78
0.83
2.00
1.02
1.05
2.72
2.22
4.53
f1 (ppm)

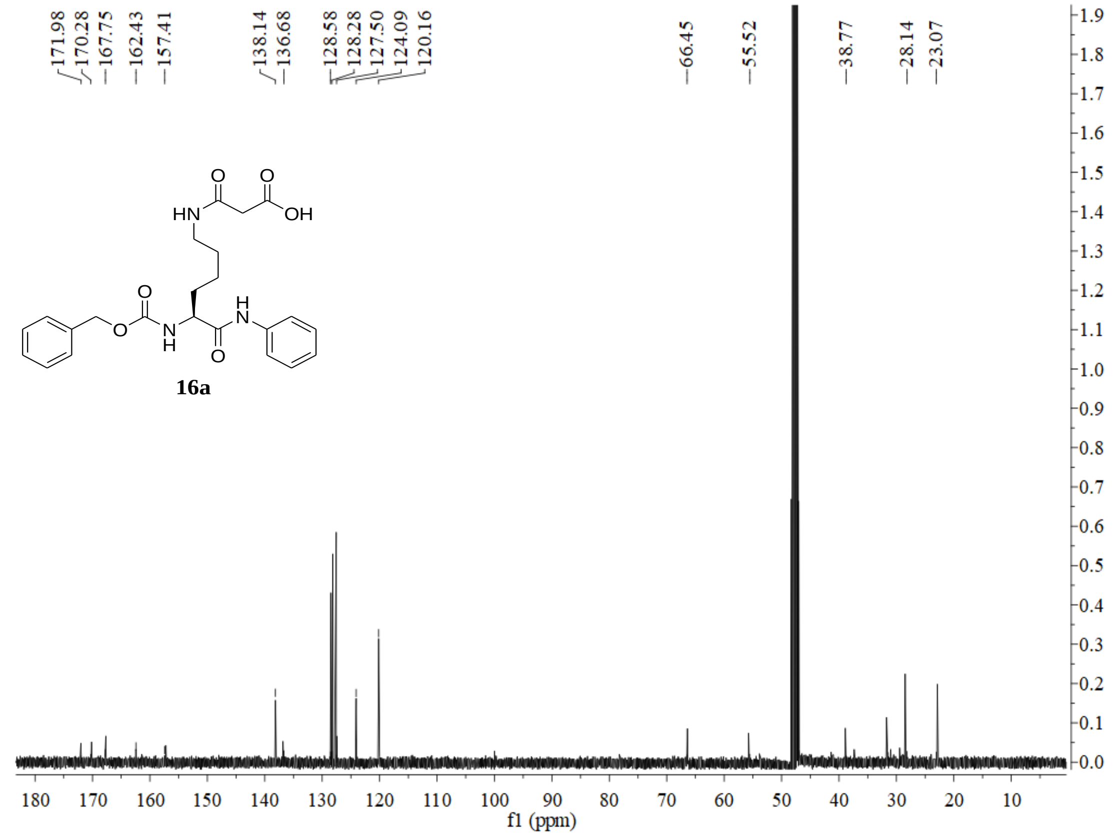
171.98
170.28
167.75
162.43
157.41
138.14
136.68
128.58
128.28
127.50
124.09
120.16
66.45
55.52
38.77
28.14
23.07
16a
f1 (ppm)

CD3OD

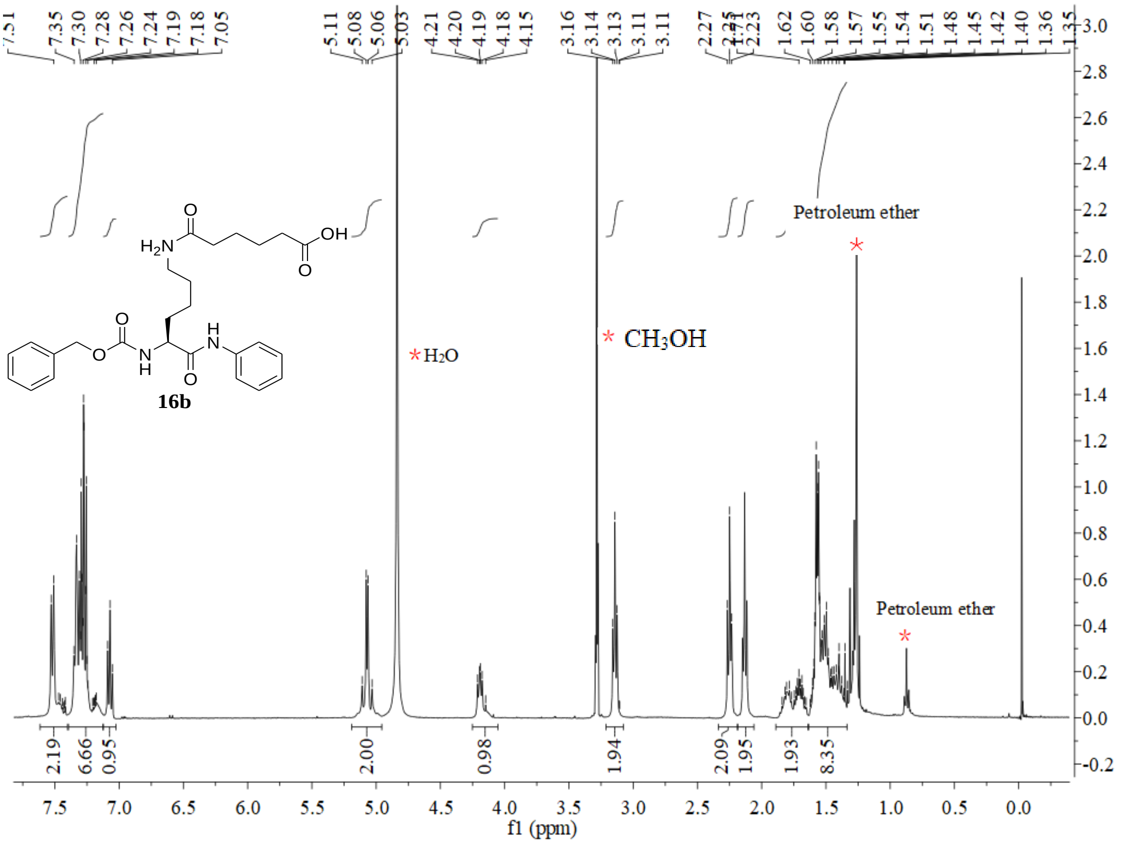

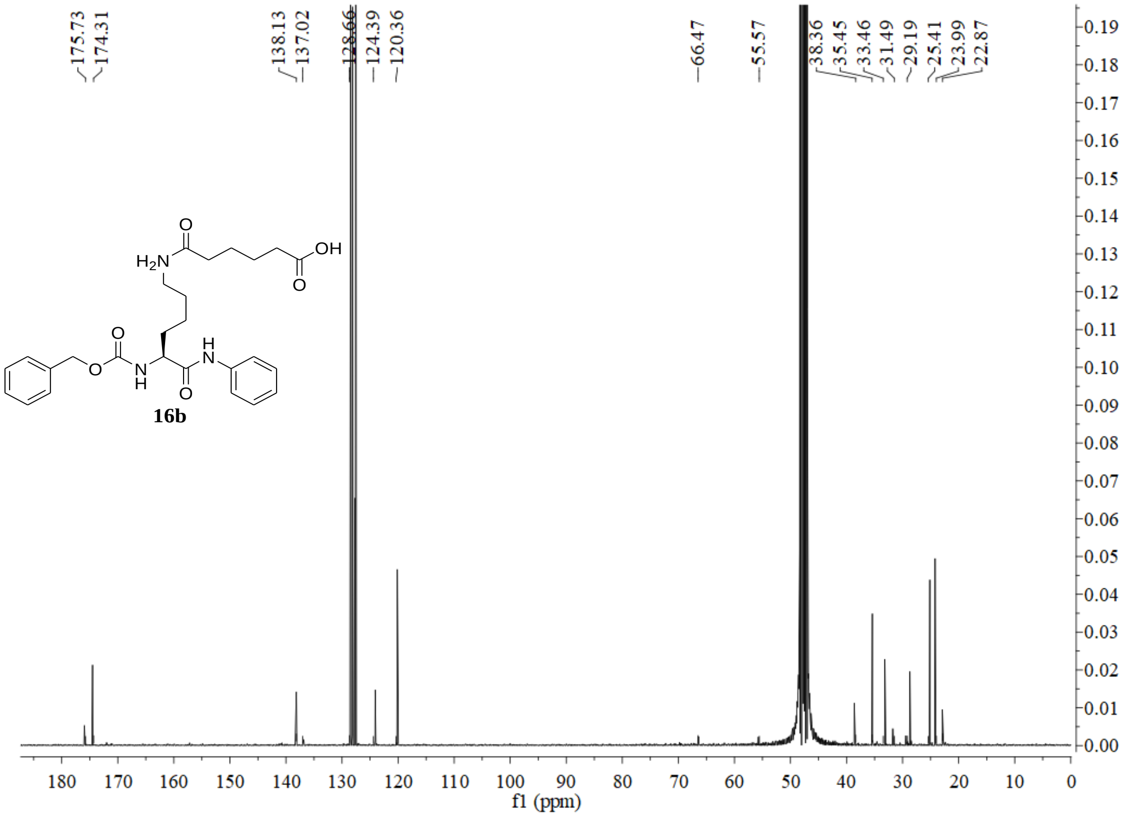

CD$_3$OD

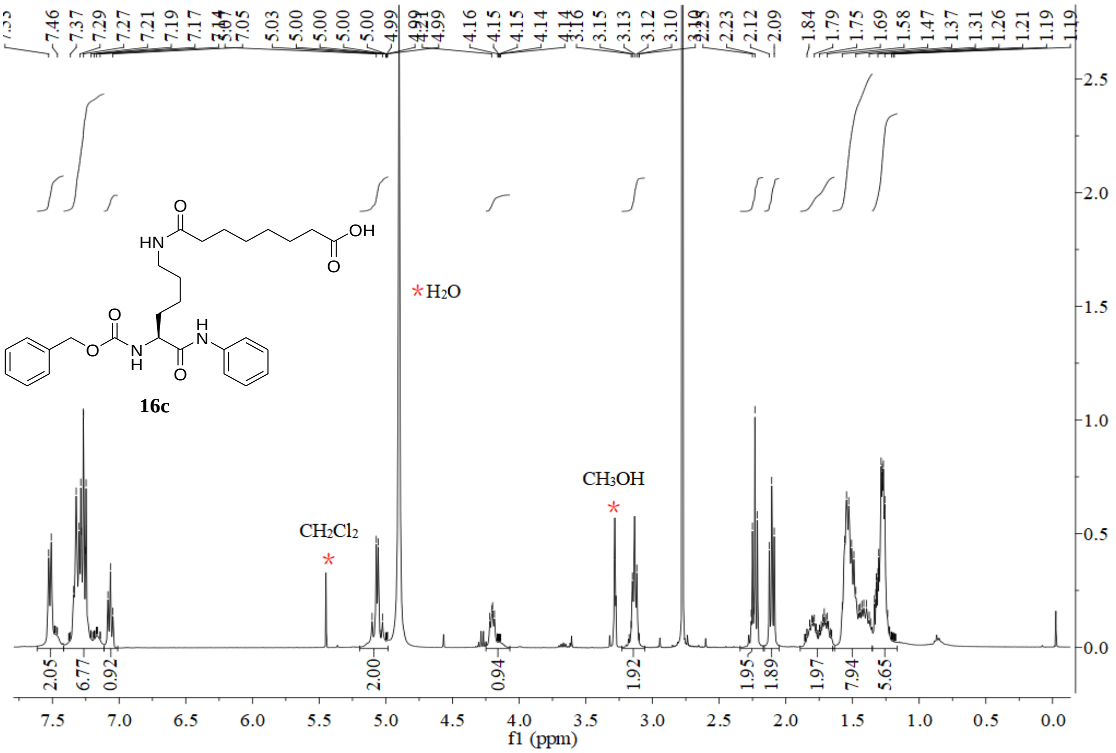
7.46
7.37
7.29
7.27
7.21
7.19
7.17
7.05
5.03
5.00
5.00
5.00
5.00
4.99
4.16
4.15
4.15
4.14
3.15
3.13
3.12
3.10
2.23
2.12
2.09
1.84
1.79
1.75
1.69
1.58
1.47
1.37
1.31
1.26
1.21
1.19
HN
O
OH
O
O
O
N
H
H
N
O
16c
*H2O
CH3OH
CH2Cl2
2.05
6.77
0.92
2.00
0.94
1.92
1.95
1.89
1.97
7.94
5.65
f1 (ppm)

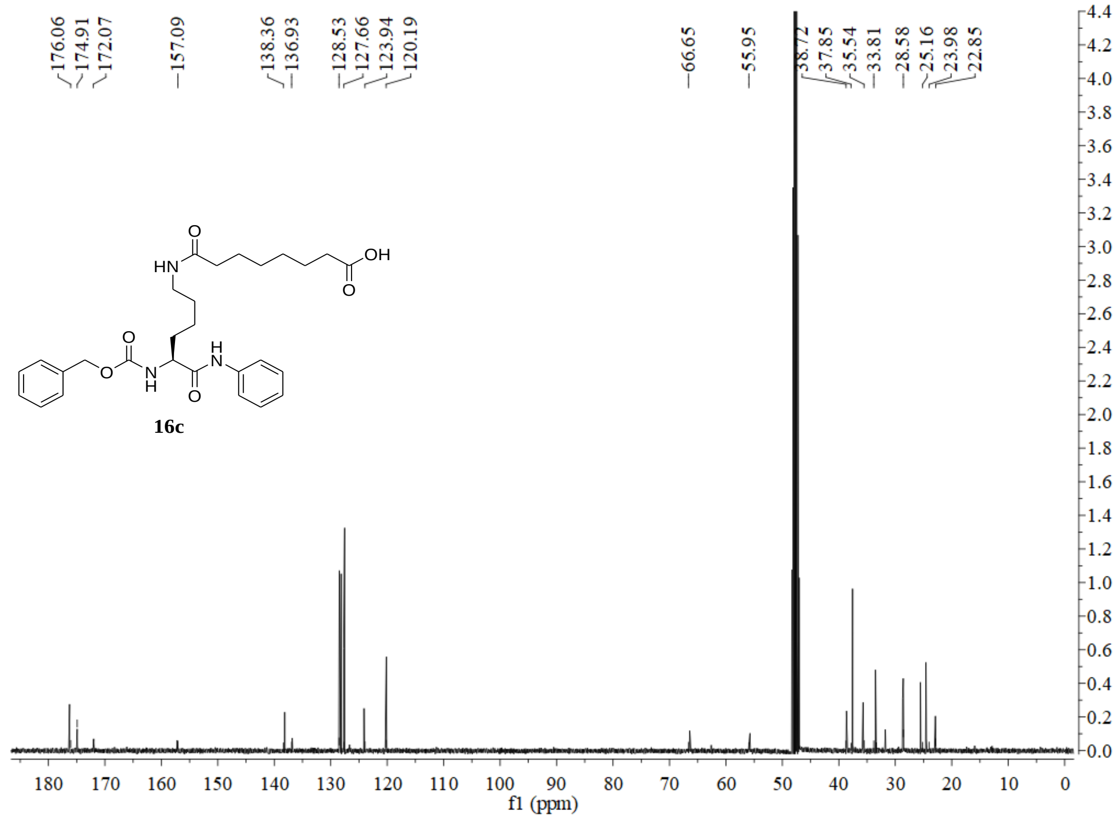
176.06
174.91
172.07
157.09
138.36
136.93
128.53
127.66
123.94
120.19
66.65
55.95
38.72
37.85
35.54
33.81
28.58
25.16
23.98
22.85
HN
O
OH
O
O
O
N
H
H
N
O
16c
f1 (ppm)

CD$_3$OD

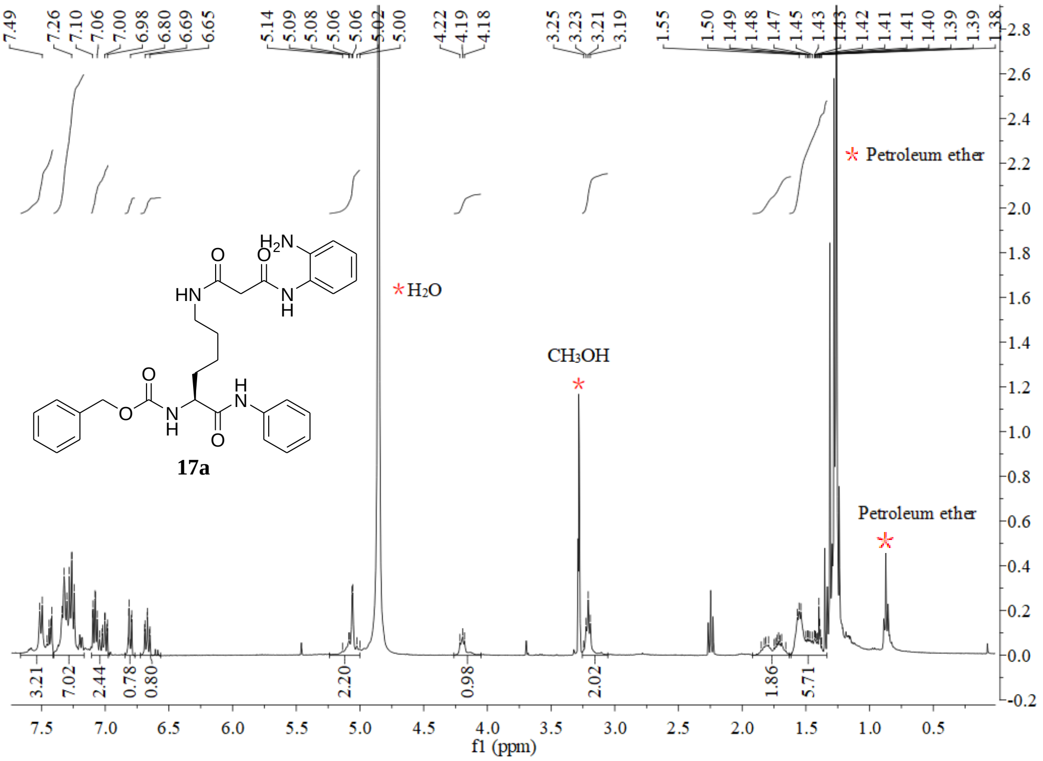
7.49
7.26
7.10
7.06
7.00
6.98
6.80
6.69
6.65
5.14
5.09
5.08
5.06
5.06
5.02
5.00
4.22
4.19
4.18
3.25
3.23
3.21
3.19
1.55
1.50
1.49
1.48
1.47
1.45
1.43
1.43
1.42
1.41
1.41
1.40
1.39
1.39
1.38
* Petroleum ether
* H2O
CH3OH
Petroleum ether
17a
3.21
7.02
2.44
0.78
0.80
2.20
0.98
2.02
1.86
5.71
f1 (ppm)

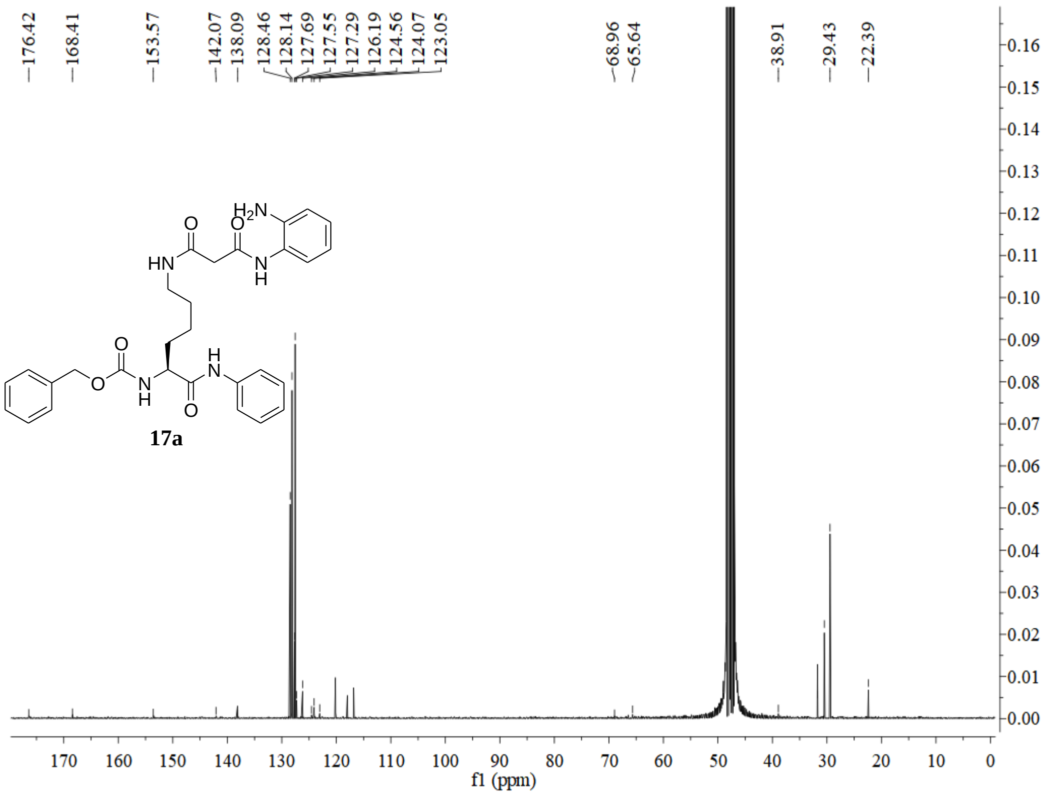
176.42
168.41
153.57
142.07
138.09
128.46
128.14
127.69
127.55
127.29
126.19
124.56
124.07
123.05
68.96
65.64
38.91
29.43
22.39
17a
f1 (ppm)

CD3OD

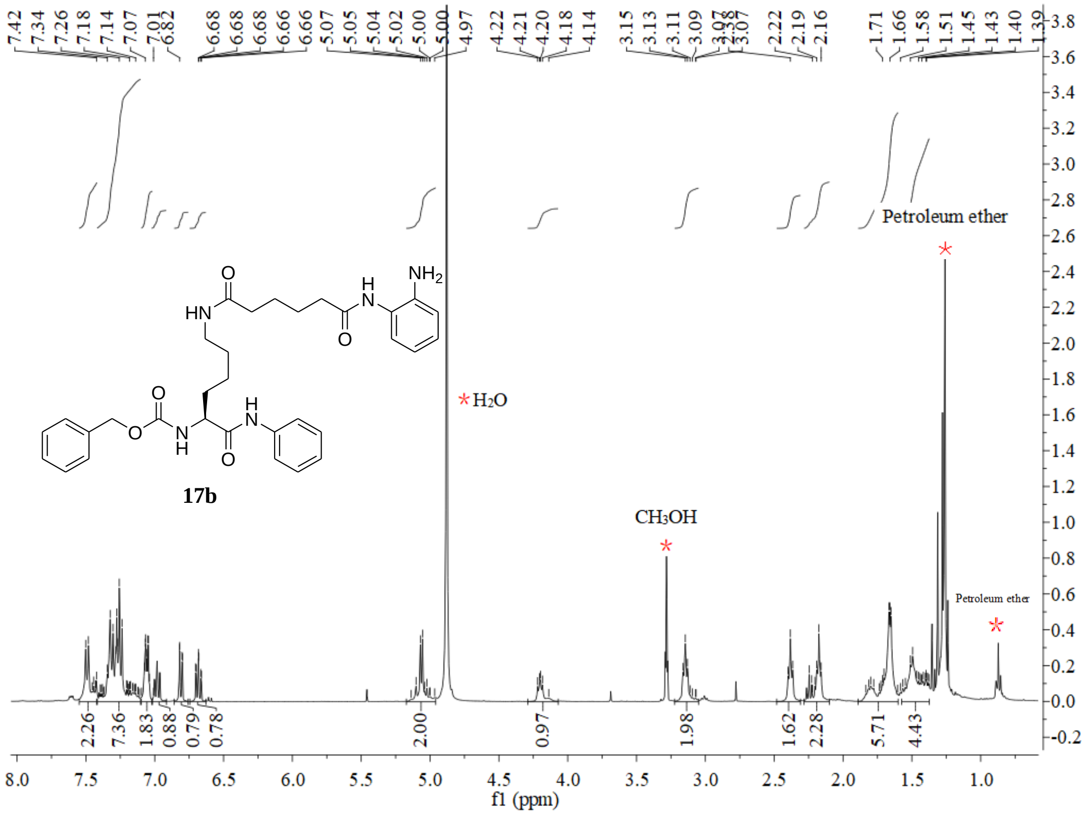

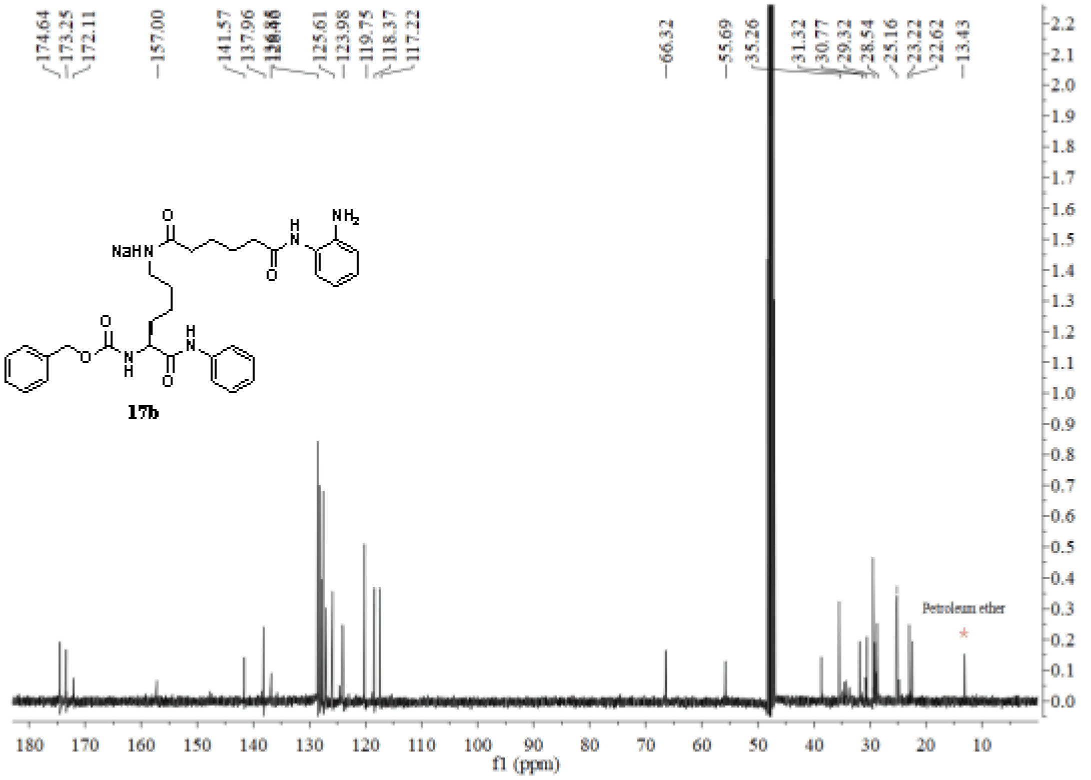

CD3OD

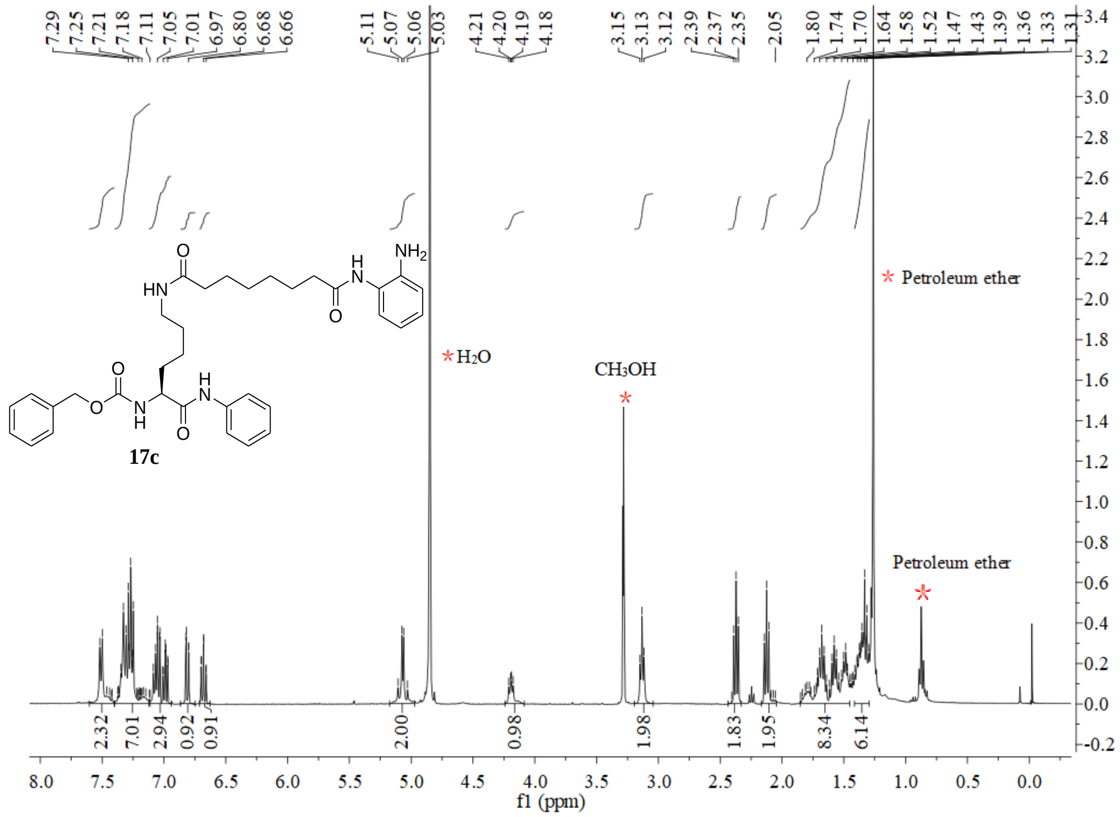

17c
*H2O
CH3OH
* Petroleum ether
Petroleum ether
f1 (ppm)

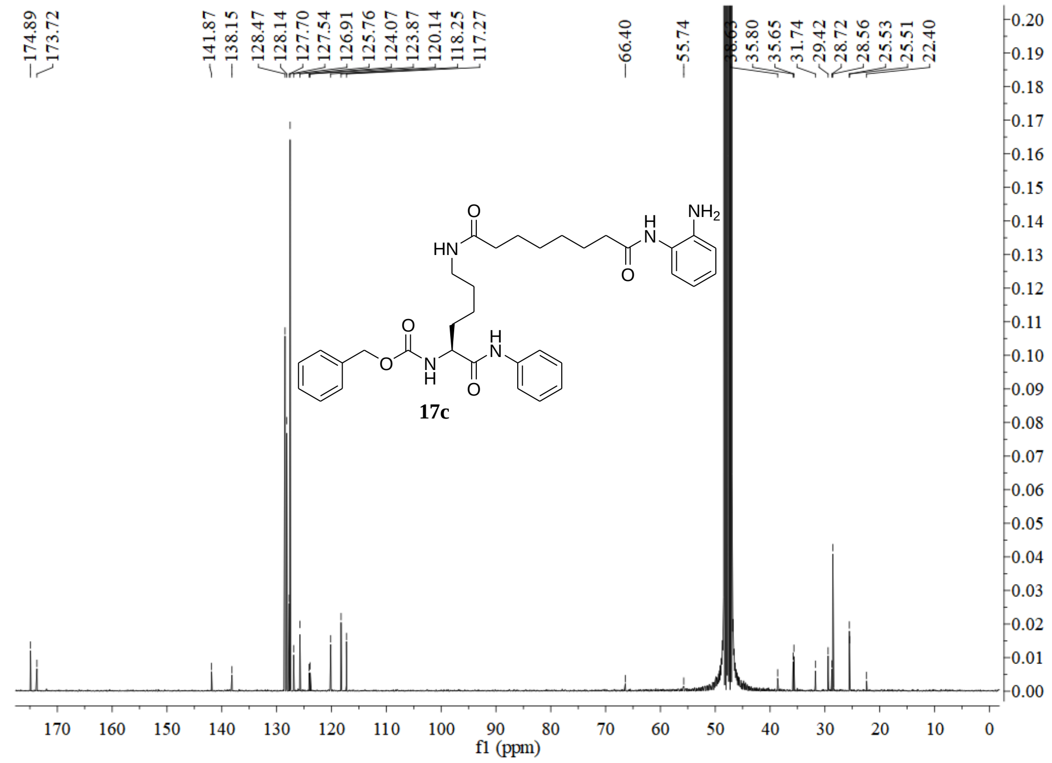

17c
f1 (ppm)

CD$_3$OD

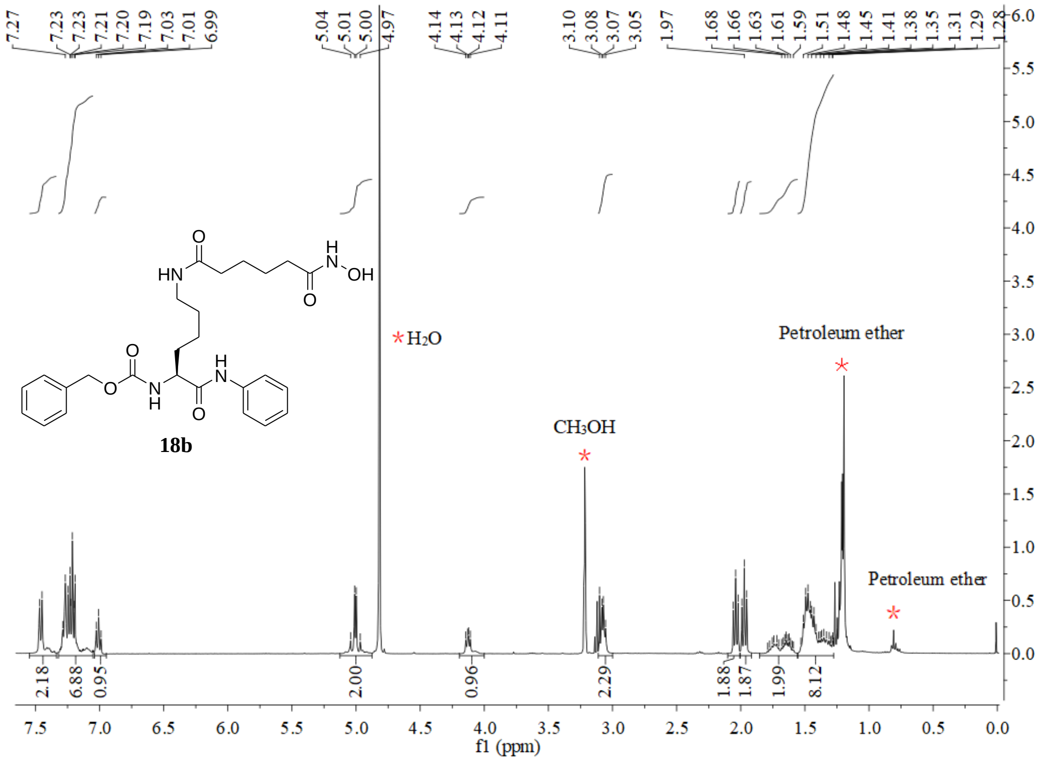
7.27
7.23
7.23
7.21
7.20
7.19
7.03
7.01
6.99
5.04
5.01
5.00
4.97
4.14
4.13
4.12
4.11
3.10
3.08
3.07
3.05
1.97
1.68
1.66
1.63
1.61
1.59
1.51
1.48
1.45
1.41
1.38
1.35
1.31
1.29
1.28
HN
O
H
N
OH
O
O
O
N
H
H
N
O
18b
*H2O
CH3OH
Petroleum ether
Petroleum ether
2.18
6.88
0.95
2.00
0.96
2.29
1.88
1.87
1.99
8.12
f1 (ppm)

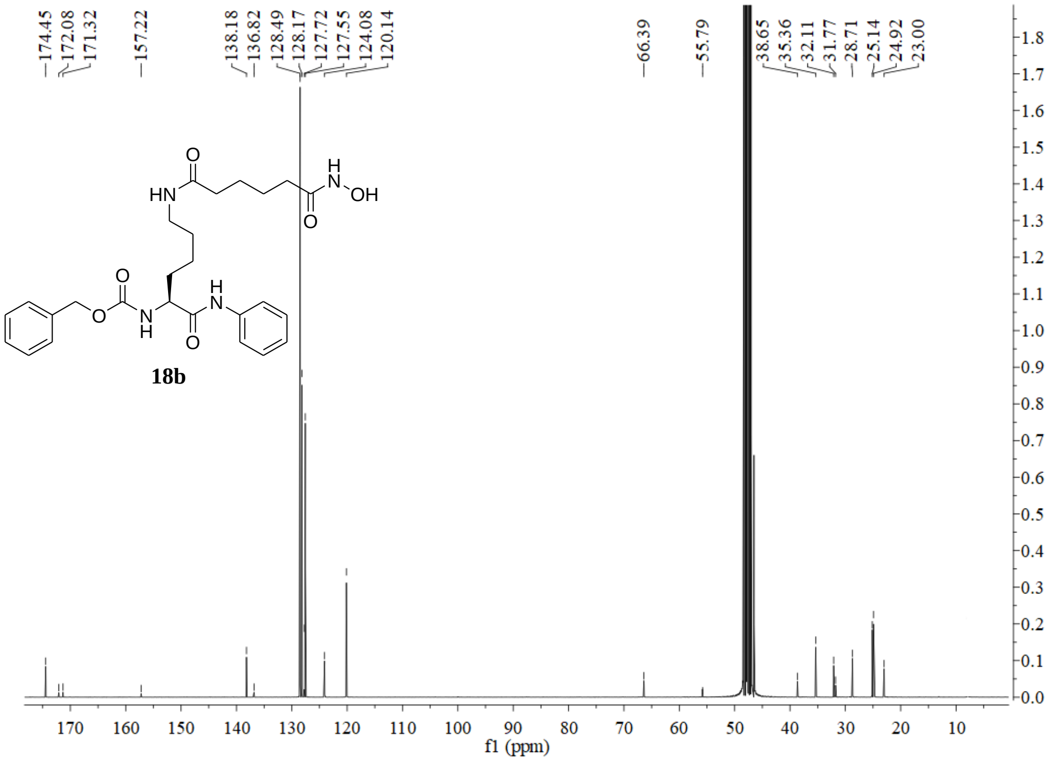
174.45
172.08
171.32
157.22
138.18
136.82
128.49
128.17
127.72
127.55
124.08
120.14
66.39
55.79
38.65
35.36
32.11
31.77
28.71
25.14
24.92
23.00
HN
O
H
N
OH
O
O
O
N
H
H
N
O
18b
f1 (ppm)

CD$_3$OD

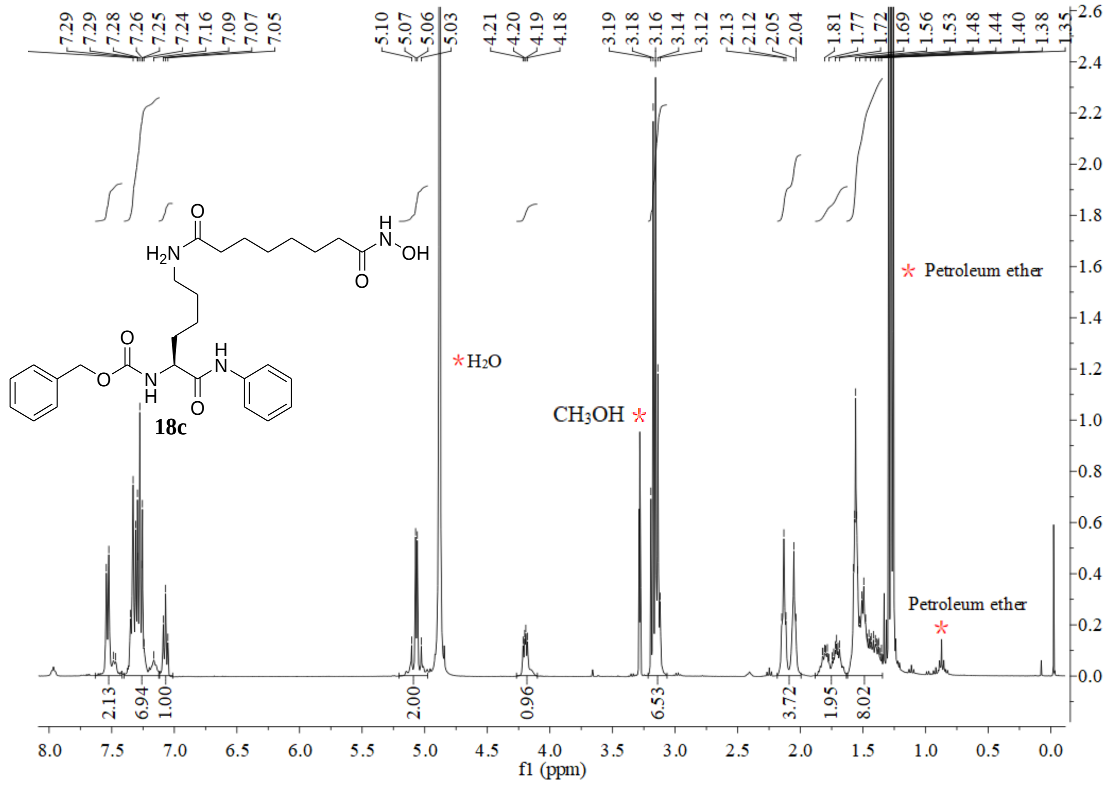

7.29 7.29 7.28 7.26 7.25 7.24 7.16 7.09 7.07 7.05
5.10 5.07 5.06 5.03
4.21 4.20 4.19 4.18
3.19 3.18 3.16 3.14 3.12
2.13 2.12 2.05 2.04
1.81 1.77 1.72 1.69 1.56 1.53 1.48 1.44 1.40 1.38 1.35
H2O
CH3OH
Petroleum ether
Petroleum ether
18c
2.13 6.94 1.00 2.00 0.96 6.53 3.72 1.95 8.02
f1 (ppm)

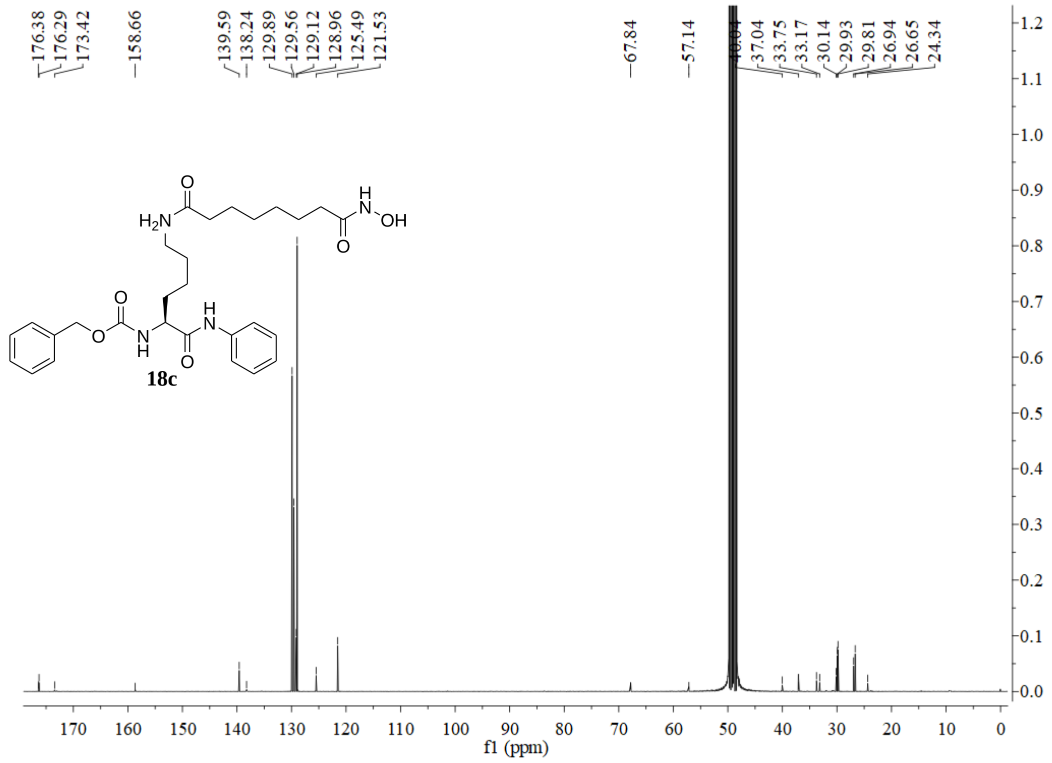

176.38 176.29 173.42 158.66 139.59 138.24 129.89 129.56 129.12 128.96 125.49 121.53
67.84 57.14 37.04 33.75 33.17 30.14 29.93 29.81 26.94 26.65 24.34
18c
f1 (ppm)
